# An engineered nano-liposome-human ACE2 decoy neutralizes SARS-CoV-2 Spike protein-induced inflammation in both murine and human macrophages

**DOI:** 10.7150/thno.66831

**Published:** 2022-03-06

**Authors:** Sandro Satta, Zhaojie Meng, Rebecca Hernandez, Susana Cavallero, Tong Zhou, Tzung K. Hsiai, Changcheng Zhou

**Affiliations:** 1 Division of Cardiology, Department of Medicine, David Geffen School of Medicine, University of California, Los Angeles, CA.; 2 Division of Biomedical Sciences, School of Medicine, University of California, Riverside, CA.; 3 Department of Physiology and Cell Biology, Reno School of Medicine, University of Nevada, Reno, NV.; 4 Department of Bioengineering, Henry Samueli School of Engineering & Applied Science, University of California, Los Angeles, CA.

**Keywords:** SARS-CoV-2, Liposome-Human ACE2, Spike Protein, IKKβ/NF-κB signaling, myeloid-specific IKKβ knockout

## Abstract

**Rationale:** Macrophages are the frontline immune cells in response to severe acute respiratory syndrome coronavirus 2 (SARS-CoV-2) infection. Angiotensin-converting enzyme 2 (ACE2) serves as the binding receptor to SARS-CoV-2 Spike glycoprotein for fusion and internalization into the human host cells. However, the mechanisms underlying SARS-CoV-2-elicited macrophage inflammatory responses remain elusive. Neutralizing SARS-CoV-2 by human ACE2 (hACE2) decoys has been proposed as a therapeutic approach to ameliorate SARS-CoV-2-stimulated inflammation. This study aims to investigate whether an engineered decoy receptor can abrogate SARS-CoV-2-induced macrophage inflammation.

**Methods:** hACE2 was biotinylated to the surface of nano-liposomes (d = 100 nm) to generate Liposome-human ACE2 complex (Lipo-hACE2). Lentivirus expressing Spike protein (D614G) was also created as a pseudo-SARS-CoV-2 (Lenti-Spike). Liposome-hACE2 was used as a decoy receptor or competitive inhibitor to inhibit SARS-CoV-2 or Lenti-Spike-induced macrophage inflammation *in vitro* and *in vivo*.

**Results:** Both SARS-CoV-2 and Lenti-Spike stimulated strong inflammatory responses by inducing the expression of key cytokine and chemokines, including IL-1β, IL-6, TNFα, CCL-2, and CXCL-10, in murine and human macrophages *in vitro*, whereas Lipo-hACE2 decoy abolished these effects in macrophages. Furthermore, intravenous injection of Lenti-Spike led to increased macrophage and tissue inflammation in wild type mice, which was also abolished by Lipo-hACE2 treatment. Mechanistically, Spike protein stimulated macrophage inflammation by activating canonical NF-κB signaling. RNA sequencing analysis revealed that Lenti-Spike induced over 2,000 differentially expressed genes (DEGs) in murine macrophages, but deficiency of IκB kinase β (IKKβ), a key regulator for NF-κB activation, abrogated Lenti-Spike-elicited macrophage inflammatory responses.

**Conclusions:** We demonstrated that the engineered Lipo-hACE2 acts as a molecular decoy to neutralize SARS-CoV-2 or Spike protein-induced inflammation in both murine and human macrophages, and activation of the canonical IKKβ/NF-κB signaling is essential for SARS-CoV-2-elicited macrophage inflammatory responses.

## Introduction

The novel coronavirus disease 2019 (COVID-19) outbreak caused by the severe acute respiratory syndrome coronavirus 2 (SARS-CoV-2) is continuing to evolve globally as the pandemic persists 1, 2. At the early stage, SARS-CoV-2 infection occurred primarily in the nasopharynx and lung epithelium in which resident macrophages are present for 90-95% of the cell population 3. Herein, the overreactive immune system releases inflammatory cytokines also known as “cytokine storm”, which can lead to multiple organ failure and ultimately death in COVID-19 patients 4-7. However, the mechanisms whereby SARS-CoV-2 induces the resident macrophage inflammation remain unknown.

It has been recognized that SARS-CoV-2 cellular entry is achieved by the homotrimeric Spike protein-mediated virus-receptor engagement through the receptor-binding domain (RBD), followed by virus-host membrane fusion 7, 8. This fusion is due to the interaction of the SARS-CoV-2 Spike protein with human angiotensin-converting enzyme 2 (hACE2), a key enzyme in the renin-angiotensin-aldosterone system (RAAS) that regulates the conversion of angiotensin II (Ang II) into angiotensin (1-7) 7, 9, 10. Non-activated macrophages, as with other immune cells, express low levels of hACE2 receptor 11, suggesting a potential hACE2-independent mechanism underlying viral particle uptake. In addition, SARS-CoV-2 Spike protein does not bind to the murine ACE2 ortholog, and others have demonstrated that there was no difference in viral load uptake by the macrophages isolated from wild-type (WT) versus hACE2 transgenic mice 12. For these reasons, human hACE2-independent mechanism is implicated to prime macrophage inflammatory responses.

Macrophages are the key frontline immune cells contributing to the pathogenesis of chronic diseases 13, 14. During cytokine storm, dysregulated macrophages release inflammatory cytokines and chemokines, including interleukin (IL)-1, IL-6, IL-12, and tumor necrosis factor (TNF)-α 5, 7, 11, 15. Many of those proinflammatory genes are regulated by the transcription factor NF-κB, a master regulator of the innate and adaptive immune responses 16-19. Indeed, NF-κB activation has been implicated in SARS-CoV-2-induced severe inflammation via pattern recognition receptors in the MyD88 pathway, leading to increased cytokine induction and release 2, 20.

To this end, we sought to block the interaction of SARS-CoV-2 Spike protein and host hACE2 receptors and to also neutralize the impact of SARS-CoV-2 Spike protein on macrophage activation. In addition to using live SARS-CoV-2 virus, we also cloned and inserted the Spike sequence into the lentiviruses (Lenti-Spike) as pseudo-SARS-CoV-2, and we biotinylated the surface of nano-liposomes (d = 100 nm) with human hACE2 (Lipo-hACE2) as a decoy. We demonstrated that SARS-CoV-2 or Lenti-Spike-induced inflammatory responses were abrogated by Lipo-hACE2 in both murine and human macrophages. Furthermore, RNA sequence analysis revealed that Lenti-Spike infection resulted in over 2,300 differentially expressed genes (DEGs) that were enriched in key pathways including “NF-κB and TNF-α signaling pathways”, “cytokine-cytokine receptor interaction”, and “Toll-like receptor signaling pathways” in control macrophages. However, deficiency of IκB kinase β (IKKβ), a key regulator for NF-κB activation, abolished Spike protein-elicited macrophage inflammatory responses. Overall, our engineered Lipo-hACE2 inhibits SARS-CoV-2-induced macrophage inflammatory responses in both murine and human macrophages, and the canonical IKKβ/NF-κB signaling was implicated in SARS-CoV-2-mediated inflammatory responses independent of hACE2 in macrophages.

## Materials and Methods

### Generation of nano-liposomes conjugated with biotinylated hACE2 proteins

Liposomes incorporating rhodamine were purchased from Encapsula Nano Sciences (Brentwood, TN, USA). The total lipid concentration in Immunosome-Biotinyl Cap was 22.45 mM. 1% mol of the lipid in liposomes contains a Biotinyl Cap group, exposing half of them to the surface of the liposomes, equal to 0.11 mM of reactive conjugable lipid. At UCLA Cardiovascular Engineering Laboratory, liposomes were reconstituted in 2mL of phosphate buffered saline (PBS) 1X at a concentration of 0.22 uM. Liposomes-Biotinyl cap were poured in a conical tube, vortexed gently, and mixed with Neutravidin Protein (Thermo Scientific, US) while vortexing at the ratio of 1:10 fold-molar liposomes:neutravidin, respectively. The solution was incubated at room temperature for 1 h during shaking (110 rpm). Next, the solution was placed at 4 °C overnight. The following day the unbound neutravidin from the prep was removed by dialysis using the Float-A-Lyzer dialysis cassette with 300K MWCO (Spectrum Labs). Liposomes/Neutravidin solution was dialyzed in 1 liter of 1X PBS at pH 7.4 for 8 h while stirring at 4 °C. After 8 h, PBS was replaced with fresh PBS for a further 8 h dialysis. After this step, Liposomes-Biotinyl Cap/Neutravidin were separated from unbound Neutravidin. The following day the liposomes-Biotinyl Cap/Neutravidin solution was poured in a conical tube and vortexed gently. During vortexing Biotinylated human-ACE2 (Acrobiosystem) was added to the Liposomes-Biotinyl Cap/Neutravidin solution at the ratio of 1:2 fold-molar Liposomes-Biotinyl Cap/Neutravidin:Biotinylated human-ACE2, His, Avitag, respectively. The solution was incubated at room temperature for 1h during shaking (110 rpm). After 1 h incubation, the solution was placed at 4 °C overnight. The Following day, the unbound neutravidin from the prep was removed by dialysis using Float-A-Lyzer dialysis cassette with 300K MWCO.

Liposomes/Neutravidin/Biotinylated-Spike hACE2 solution was dialyzed in 1 liter of 1X PBS at pH 7.4 while stirring at 4 °C. After 8 h the 1X PBS was replaced with fresh 1X PBS for a further 8 h dialysis. After this step, Liposomes/Neutravidin/Biotinylated-hACE2 preparation was separated from the unbound hACE2. Next, the purified immunoliposome solution was ready to be used. We did not observe the significant changes with the size of liposome (d = 100 nm) by using electron microscope (data not shown). Stock concentration of 0.22 uM Liposomes/hACE2 was diluted for the experiments.

### Liposomes hACE2 complex validation through ELISA

We validated the correct complex liposome-hACE2 by using a Human ACE2 ELISA Kit. The liposome-hACE2 complex was incubated in the ACE2 ELISA plate and the fluorescence from the rhodamine incorporated in the liposomes was measured at λEx 546 nm and λEm 568 nm. Next anti-hACE2 antibodies were added to the wells and the absorbance was measured at 450 nm OD according to manufacture. The double signal (fluorescence and absorbance) from the same wells confirmed the successful construction of the liposome-hACE2 complex.

### Generation of Lentivirus for expressing Spike-mutation (D614G) protein

Expression of cloned DNA (625 ng) was mixed with packaging plasmids pVSV-G and pCMV delta R8.2 at the ratio of 8:1 by mass. Viafect transfection reagent (Promega) was incubated with OPTIMEM (Gibco) (1:12.5, v/v) for 20-30 min at room temperature, prior to adding to the DNA mix at 31 ml per well. HEK293T cells were plated to 70-90% confluence prior to transfection. After 16 h, the medium was replaced with fresh medium. After 48 h, the virus was harvested and filtered in 45 μM in a 15 mL tube. Lenti-X concentrator (Takara) was mixed with the medium containing the virus in the ratio of 1:3 media:Lenti-X, respectively. The mixture was left overnight at 4 °C, and 45 min centrifugation was performed at 1500 xg. The supernatant was removed by aspiration and the pellet was resuspended in 400 μL of 1X PBS (Gibco).

### SARS-CoV-2 infection experiments

SARS-CoV-2, B.1.526, was acquired from the Biodefense and Emerging Infections (BEI) Resources of the National Institute of Allergy and Infectious Diseases (NIAID) via UCLA Virology Program. SARS-CoV-2 was passaged once in Vero-E6 cells (ATCC), and viral stocks were aliquoted and stored at -80 °C. Virus titer was determined by plaque assay using Vero E6 Cells. 2.5x10^5^ pfu/mL of SARS-CoV2 was used for the experiments. Studies involving live SARS-CoV-2 virus were approved by the University of California, Los Angeles Institutional Biosafety Committee (IBC) and were performed in the UCLA Biosafety Level 3 (BSL3) high-containment facility.

### AlamarBlue assays for inhibitory effects

AlamarBlue (AB) assays were used to elucidate the inhibitory effect on Cytopathic effect (CPE) induction caused by SARS-CoV-2 infection. Briefly, Vero E6 cells (infected and non-infected) were incubated with Dulbecco's Modified Eagle Medium (DMEM) without FBS or supplements, and AB solution (5% [v/v] solution of AB dye). Following 3 h incubation, AB absorbance was quantified at 600 nm using a Fisher AccuSkan FC microplate reader. Twelve technical replicates per experiment at concentration of 25 nM was carried out (n = 5) for cytopathic effect evaluation of SARS-CoV-2 in the presence of free-hACE2 protein or Lipo-hACE2. The reading was performed in triplicate (the median values obtained from 3 different wells was calculated). The relative cell viability rate (%) was calculated by comparing infected and non-infected wells [(mean fluorescence of treated-SARS-CoV-2 infected wells/mean fluorescence of treated non-infected wells) × 100]. Each assay was carried out in triplicate. To score SARS-CoV-2 induced CPE, we performed microscopy to image the infected cells in the presence or absence of Lipo-hACE2 or free hACE2 treatment.

### Animals

C57BL/6J wild-type (WT) mice were purchased from The Jackson Laboratory (Bar Harbor, ME). Myeloid-specific IKKβ knockout (IKKβ^ΔMye^) mice on C57BL/6J background (The Jackson Laboratory, Bar Harbor, ME) were generated by crossing mice carrying loxP-flanked IKKβ alleles (IKKβ^F/F^) with LysM-Cre transgenic mice, as previously described 21, 22. All animals were housed in a pathogen-free environment with a 12 h light-dark cycle under an approved protocol. Eight-week-old male WT mice were intravenously (IV) injected with 100 µL of Lenti-spike (1.0x10^6^ pfu/mL) or control lentivirus followed by IV injection of 100 µL control liposome or Lipo-hACE2 (4 nM) 1 h post infection. On the day of euthanasia, mice were fasted for 6 h following the dark cycle (feeding cycle), and blood and tissues were collected as described previously 19, 22.

### Macrophage isolation and treatment

Peritoneal Macrophages were isolated from WT, IKKβ^F/F^, and IKKβ^ΔMye^ mice as previously described 21-23. For lentivirus infection, macrophages were infected with lentivirus expressing SARS-CoV-2 Spike protein (Lenti-Spike) (2.3x10^7^ pfu/mL) or control lentivirus for 1 h followed by treatment with 25 nM Lipo-hACE2 or control liposome for another 23 h. In the UCLA BSL3 Laboratory, macrophages were infected with SARS-CoV-2 (2.5×10^5^ pfu/mL) or inactivated SARS-CoV-2 (control virus) for 1 h followed by treatment with 25 nM Lipo-hACE2 or control liposome for 23 h.

### RNA Isolation and Quantitative Real-Time PCR Analysis

Total RNA was isolated from mouse tissues or cells using TRIzol Reagent following the manufacture's protocol (Thermo Fisher Scientific, Waltham, MA) as previously described 24. Total RNA was reverse-transcribed using SuperScript III reverse transcriptase according to the manufacturer's instructions (Thermo Fisher Scientific, Waltham, MA) 24. Quantitative real-time PCR was performed using gene-specific primers and SYBR Green Supermix (Bio-Rad, Hercules, CA) using a CFX Real-Time PCR Instrument (Bio-Rad, Hercules, CA) as per the manufacturer-supplied protocol. For each biological sample, two technical replicate cycle threshold (Ct) values were collected and averaged. The mean Ct values were normalized to glyceraldehyde-3-phosphate dehydrogenase (GAPDH), and the relative mRNA expression levels were calculated using the comparative ΔΔCt method 24, 25. The relative gene expression was presented as mean fold change over control samples. All oligonucleotides were purchased from Sigma-Aldrich and the sequences of primer sets used in this study are listed in **Supplemental Table 1**.

### RNA sequencing and data analysis

Peritoneal macrophages were isolated from 8-week-old male IKKβ^F/F^ and IKKβ^ΔMye^ mice as previously described 22, 23. Macrophages were seeded to the cell culture plates for 4 h and then treated with Lenti-Spike or control virus for 24 h. Total RNA was extracted, and RNA integrity was confirmed using a dual Agilent 2100 Bioanalyzer (Agilent Technologies Inc., Santa Clara, CA). The creation of cDNA libraries and sequencing were performed using the Illumina standard operation pipeline as previously described 22, 26-28. For data analysis, we applied the *Salmon* tool 29 to quantify the mRNA expression from the raw sequencing data, using the *Ensembl* 30 mouse gene annotation (GRCm38). Transcript per million reads (*TPM*) was used as the unit of mouse gene expression level. We then used the *edgeR* algorithm 31 to compare the groupwise transcriptomic pattern. We also applied the *TMM* algorithm implemented in the *edgeR* package to perform reads count normalization and effective library size estimation. Group-wise differential expression was estimated by the likelihood ratio test included in the *edgeR* package. The genes with a false discovery rate (*FDR*) < 1% and fold change (*FC*) >3 was deemed differentially expressed. We further performed gene ontology analysis upon the differentially expressed genes using the definition from Kyoto Encyclopedia of Genes and Genomes (KEGG) 32 and Gene Ontology (GO) 33 projects. For each KEGG pathway or GO Biological Process term, we computed a geneset score, using the Functional Analysis of Individual Microarray Expression (*FAIME*) algorithm 34. Briefly, FAIME computes geneset scores using rank-weighted gene expression data of individual samples, which converts each sample's genome-wide gene expression profile into molecular mechanisms 34. A higher geneset score indicates higher overall expression of a given KEGG pathway. All RNAseq datasets have been deposited in the Gene Expression Omnibus (GSE182264).

### Immunofluorescence staining

Immunofluorescence staining was performed on sections of lung and heart aortic roots that were freshly embedded in OCT 19. The slides were fixed in ice-cold acetone for 15 min and permeabilized with PBS + 0.1% Triton X-100 (PBST) for 15 min and blocked with PBST containing 5% BSA (MilliporeSigma, St. Louis, MO) for 1 h at room temperature. The sections were then incubated with antibodies against macrophage marker CD68 (1:100; Bio-Rad Laboratories) and interleukin 6 (IL-6, 1:100; Bio-Rad Laboratories) at 4 °C overnight. The slides were rinsed with PBS 1X and incubated with the corresponding secondary antibodies at 1:500 (Life Technologies, Carlsbad, CA). The nuclei were stained by mounting the slides with 4', 6-diamidino-2-phenylindole (DAPI) medium (Vector Laboratories). Images were acquired under a fluorescence microscope (Nikon).

### Statistical Analysis

All data were presented as the mean ± SEM except for the high-throughput sequencing. Individual pairwise comparisons were analyzed by two-sample, two-tailed Student's *t* test unless otherwise noted, with *P* < 0.05 to be statistically significant. One-way ANOVA with Dunnett's test was used to assess the difference among more than 2 groups. Two-way ANOVA was used when multiple comparisons were made, followed by a Bonferroni multiple comparisons test.

## Results

### An engineered Lipo-hACE2 decoy inhibits SARS-CoV-2-induced cell death

**Figure 1:**
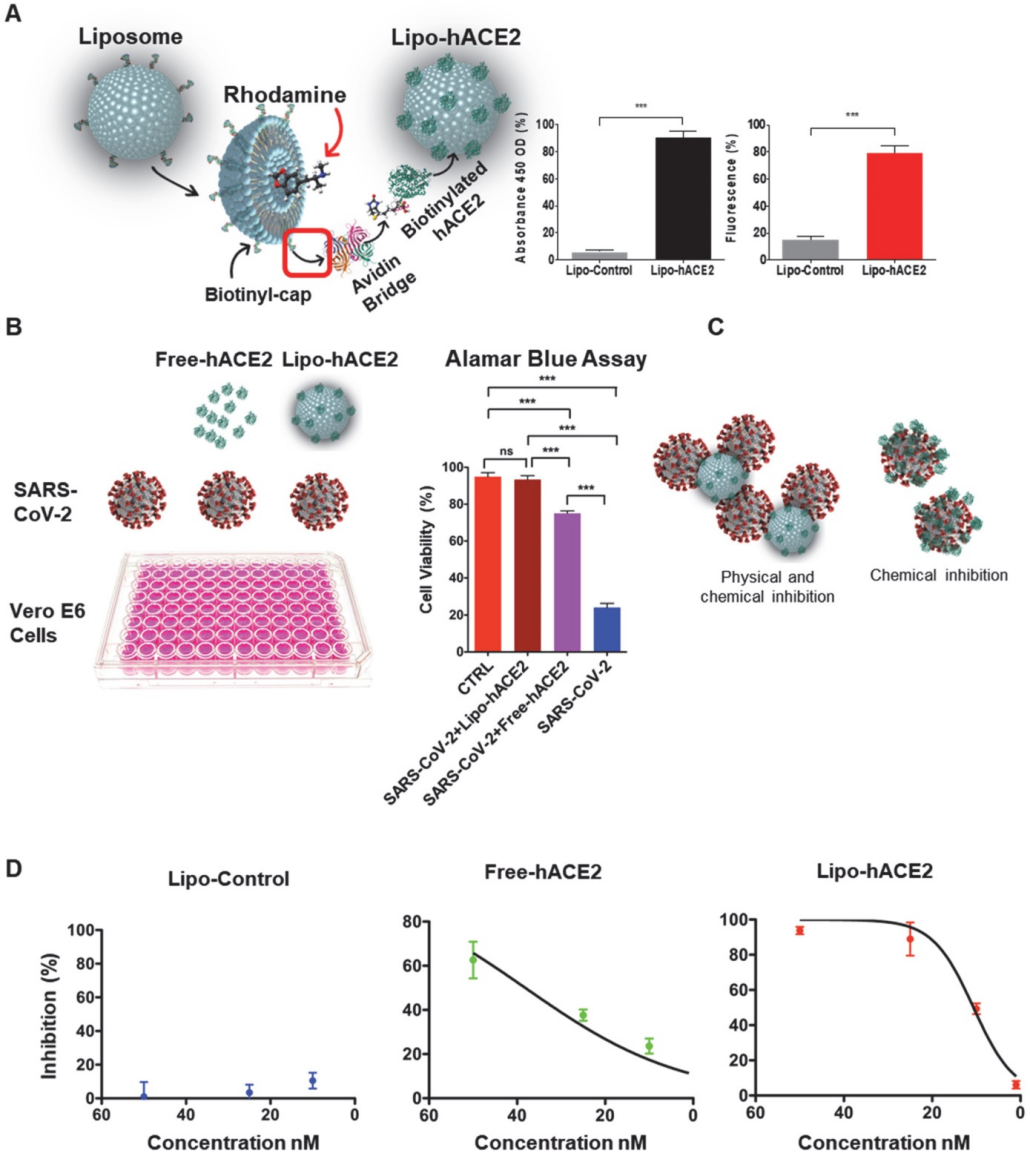
** Generation of liposome-hACE2 complex that prevents SARS-CoV-2-induced cell death. (A)** Liposomes coated with human-ACE2 protein (Lipo-hACE2) were created via biotin-neutravidin bridge. Double signal from hACE2 absorbance (450nm OD) and rhodamine incorporated (λ^Ex^ 546 nm, λ^Em^ 568 nm) in 100nm lipoparticles confirmed the correct formation of the lipo-hACE2 decoy results are displayed as a percentage (n = 12; ***P < 0.001). **(B)** Vero E6 cells were infected with SARS-CoV-2 (2.5x10^5^ pfu/mL) alone or in combination with free-hACE2 protein (25 nM) or liposome-hACE2 (25 nM). Cell viability was measured after 48 h via AlamarBlue assay (n = 5; ***P < 0.001). **(C)** Schematic representation on how lipo-hACE2 inhibits SARS-CoV-2. Despite similar chemical affinity between free-hACE2 protein and Lipo-hACE2 for the SARS-CoV-2 spike proteins, Lipo-hACE2 also offers physical inhibition due to the liposomes size (100 nm) which may potentially enhance therapeutic effects. **(D)** Dose-response (5 to 50 nM) QPCR analysis was carried out to assess the lipo-hACE2 efficiency in terms of SARS-CoV-2 inhibition. Lipo-Control was used as negative control and free-hACE2 protein was used to compare inhibition efficiency.

We engineered the liposome surface conjugated hACE2 (Lipo-hACE2) for targeting SARS-CoV-2 Spike protein. We validated that rhodamine encapsulated liposomes were biotinylated with hACE2 at an excitation wavelength of 546 nm and emission of 568 nm. Both the fluorescence emission and absorbance signals were measured at 450 nm OD to confirm the conjugation of liposomes with hACE2 (Figure 1A).

Next, Vero E6 cells were infected with SARS-CoV-2 B.1.526 at a multiplicity of infection (MOI) of 0.05, along with lipo-hACE2 or free-hACE2. Both free-hACE2 and Lipo-hACE2 inhibited SARS-CoV-2-induced cell death, whereas Lipo-hACE2 treatment significantly improved cell viability in SARS-CoV-2-infected cells to a greater extent than free-hACE2 proteins at the same concentrations (Figure 1B and C). Notably, Lipo-hACE2 inhibited SARS-CoV-2-mediated Vero E6 cell death at 25 nM, comparable to the uninfected cells. Furthermore, we also evaluated the efficiency of Lipo-hACE2 on inhibiting SARS-CoV-2 replication as compared to free hACE2 protein. We also included liposome without hACE2 conjugation as a negative control. QPCR analysis showed dose-dependent inhibitory responses for both Lipo-hACE2 and free hACE2 (Figure 1D). However, Lipo-hACE2 showed stronger inhibitory responses with IC_50_ = 10.68 nM as compared with free hACE2 (IC_50_ = 38.19 nM).

### Lipo-hACE2 abrogates Lenti-Spike or SARS-CoV-2-induced murine macrophage inflammation

**Figure 2:**
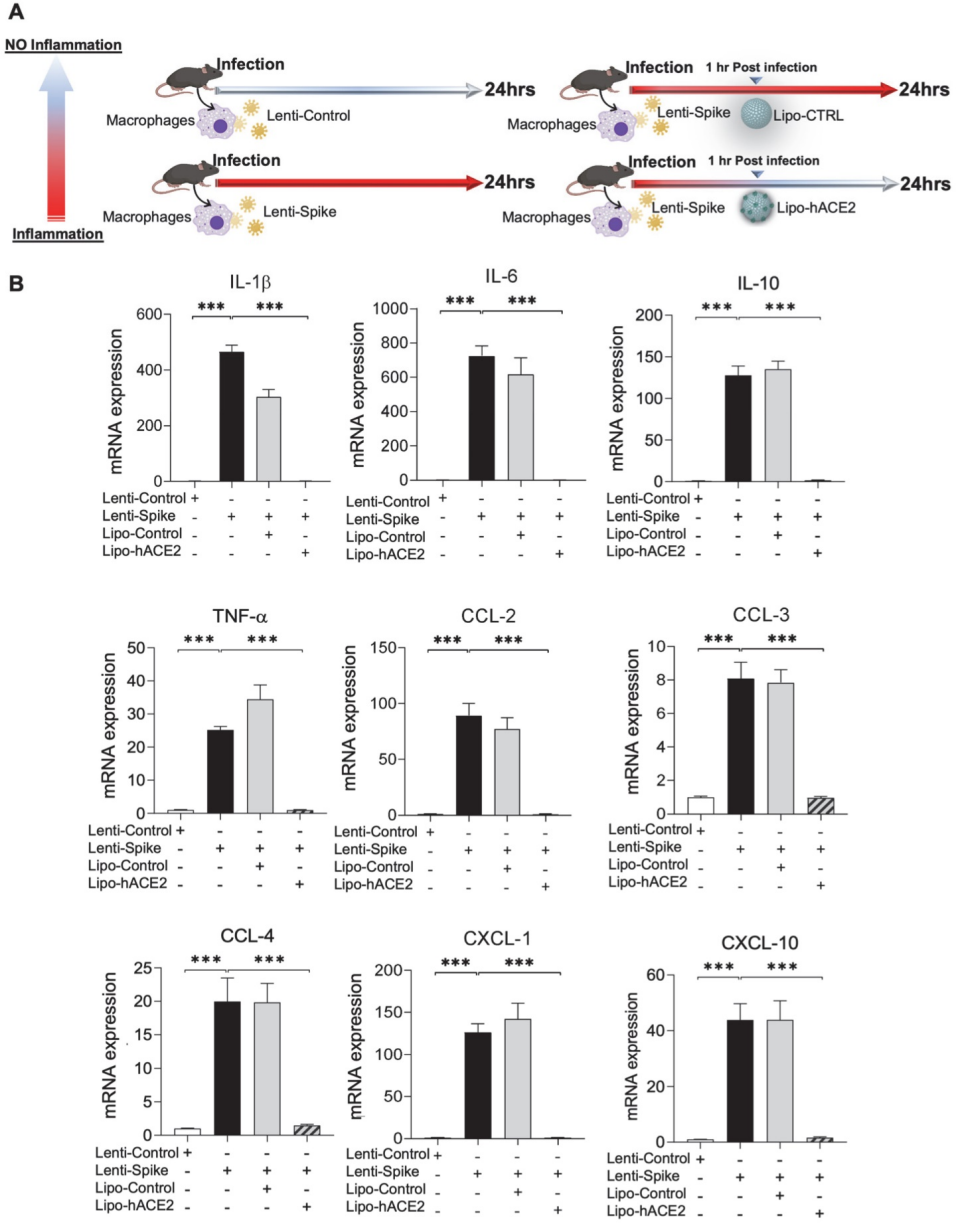
** SARS-CoV-2 Spike protein-induced inflammatory responses are inhibited by Liposome-hACE2 complex in murine macrophages. (A)** Peritoneal macrophages were isolated from eight-week-old C57BL6/J wild-type (WT) mice. The cells were infected with Lentivirus expressing SARS-CoV-2 Spike protein (Lenti-Spike) (2.3x10^7^ pfu/mL) or control lentivirus for 1 h followed by treatment with 25 nM Lipo-hACE2 or control liposome for another 23 h. **(B)** Total RNA was extracted, and the expressions of inflammatory cytokines and chemokines were analyzed by QPCR (n = 5; ***P < 0.001).

**Figure 3:**
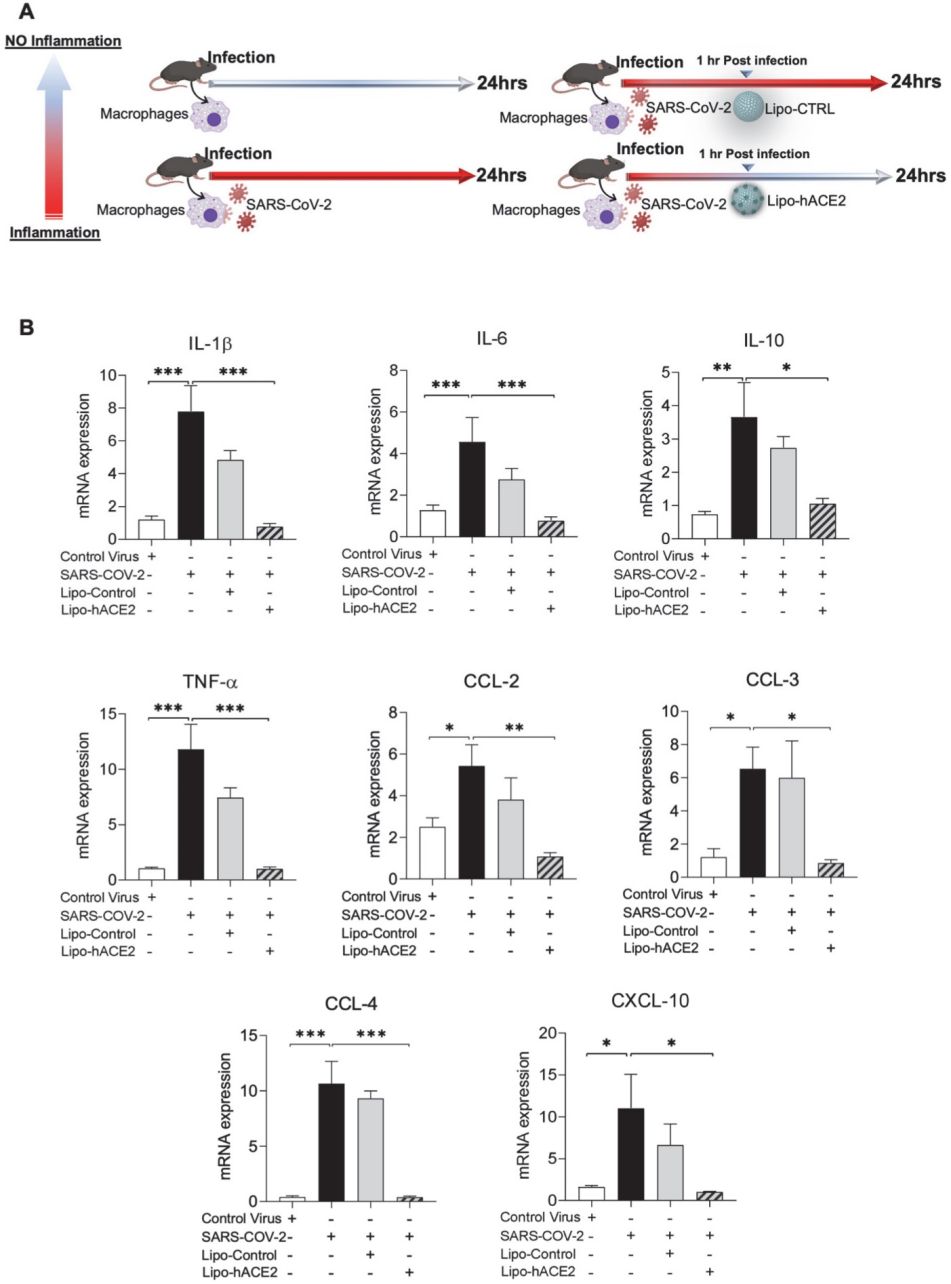
** Liposome-hACE2 complex ameliorates SARS-CoV-2-induced inflammatory responses in murine macrophages. (A)** Peritoneal macrophages were isolated from eight-week-old C57BL6/J wild-type (WT) mice. The cells were infected with SARS-CoV-2 (2.5x10^5^ pfu/mL) or inactivated SARS-CoV-2 (control virus) for 1 h followed by treatment with 25 nM Lipo-hACE2 or control liposome for 23 h. **(B)** Total RNA was extracted and the expressions of inflammatory cytokines and chemokines were analyzed by QPCR (n = 5; *P < 0.05, **P < 0.01 and ***P < 0.001).

To demonstrate Spike protein-induced macrophage inflammatory responses, we isolated peritoneal macrophages from WT mice (Figure 2A). Lenti-Spike infection significantly increased the expression of inflammatory cytokines, including IL-1β, IL-6, and TNF-α, and chemokines, including CCL-2, CCL-3, CCL-4, and CXCL-10, whereas Lipo-hACE2 decoy significantly inhibited all these effects (Figure 2B). Interestingly, Lenti-Spike treatment also led to increased expression of IL-10 in murine macrophages (Figure 2B). IL-10 is considered as an anti-inflammatory cytokine but has also been found to be upregulated in COVID-19 patients 35-37. Similarly, SARS-CoV-2-induced cytokine and chemokine expression were abolished by Lipo-hACE2 treatment in the macrophages (Figures 3).

### Lipo-hACE2 abolishes Lenti-Spike-induced macrophage and tissue inflammation *in vivo*

**Figure 4:**
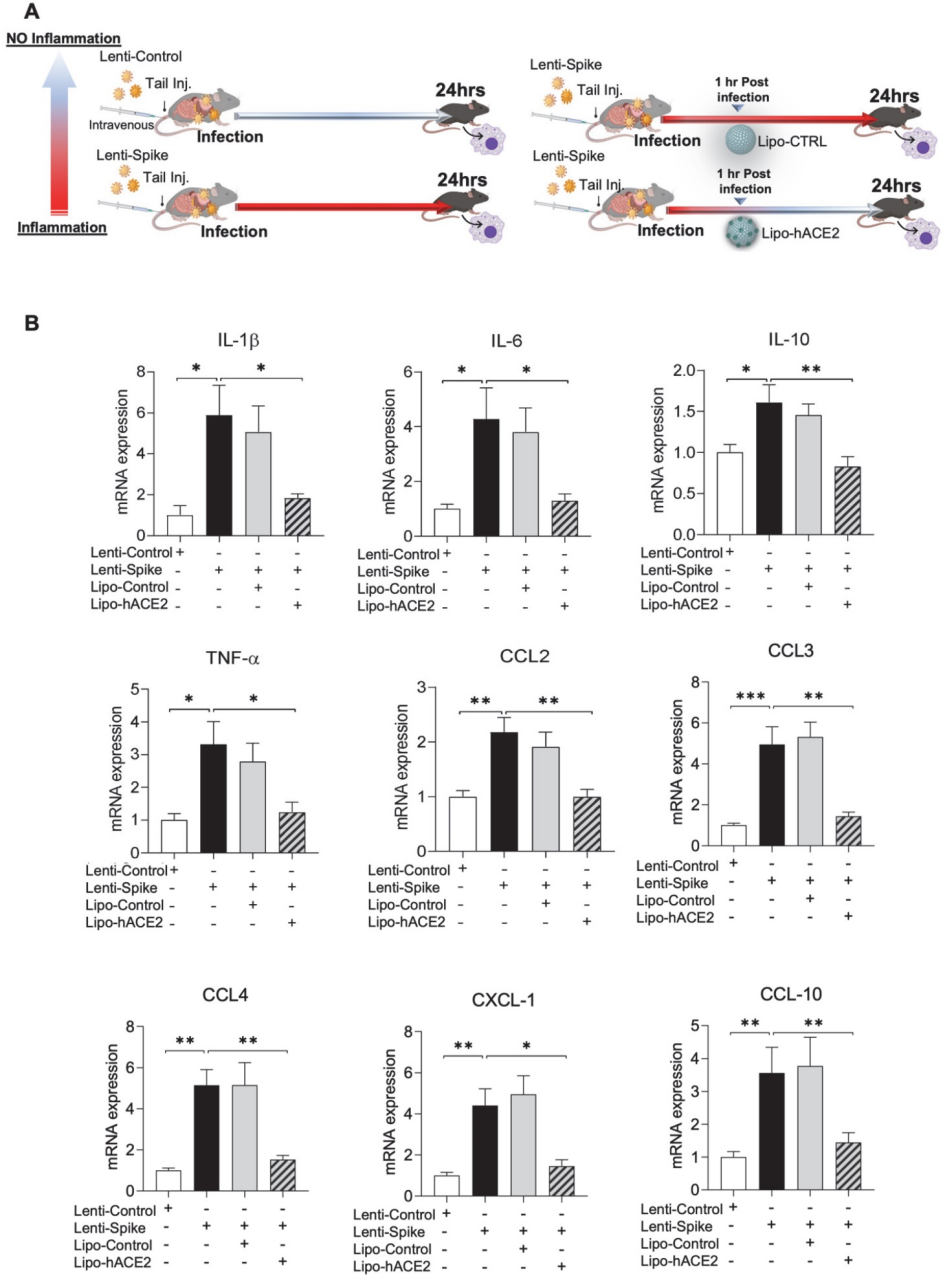
** SARS-CoV-2 Spike protein-elicited macrophage inflammatory responses are attenuated by Liposome-hACE2 complex *in vivo*. (A)** Eight-week-old male WT mice were injected IV with 100 µL of Lenti-spike (1.0x10^6^ pfu/mL) or control lentivirus followed by IV injection of 100 µL control liposome or Lipo-hACE2 (4 nM) 1 h later. After 24 h, mice were euthanized, and peritoneal macrophages were isolated for analysis. **(B)** Peritoneal macrophages were then lysated and total RNA was extracted. The expression of inflammatory cytokines and chemokines were analyzed by QPCR (n = 6; *P < 0.05, **P < 0.01 and ***P < 0.001).

**Figure 5:**
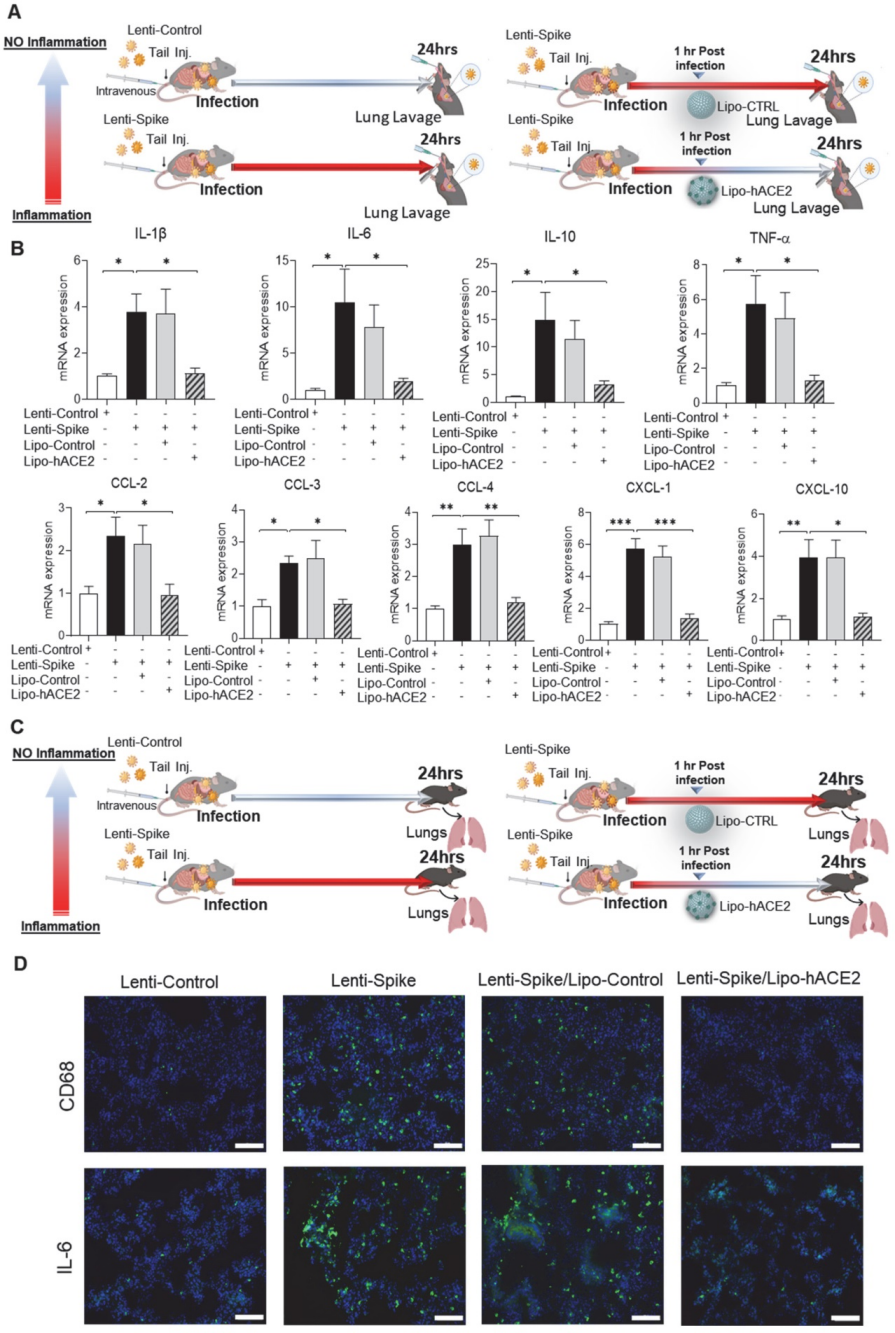
** Liposome-hACE2 complex ameliorates SARS-CoV-2 Spike protein-induced lung inflammation *in vivo*. (A)** Eight-week-old male WT mice were injected IV with 100 µL of Lenti-spike (1.0x10^6^ pfu/mL) or control lentivirus followed by IV injection of 100 µL control liposome or Lipo-hACE2 (4 nM) 1 h later. After 24 h, mice were euthanized, and bronchoalveolar lavage fluid was collected for analysis. **(B)** Total RNA was extracted from bronchoalveolar lavage fluid, and the expression of inflammatory cytokines and chemokines were analyzed by QPCR (n = 6; *P < 0.05, **P < 0.01 and ***P < 0.001). **(C)** WT mice were injected IV with Lenti-spike or control lentivirus followed by IV injection of 100 µL control liposome or Lipo-hACE2 1 h later. After 24 h, mice were euthanized, and lung was collected for immunostaining. **(D)** Representative images of immunofluorescence staining of macrophage marker CD68 (top) and IL-6 (bottom) in the lung of WT mice. The nuclei were visualized with DAPI (blue) (scale bar, 100 µm).

The efficacy of Lipo-hACE2 decoy was assessed in WT mice (Figure 4A). Tail-vein injection of Lenti-Spike increased cytokine expression, including IL-1β, IL-6, TNF-α, and CCL-2 in peritoneal macrophages, whereas Lipo-hACE2 decoy abolished Lenti-Spike-induced inflammatory effects (Figure 4B). Furthermore, Lenti-Spike significantly increased the cytokine expressions in bronchoalveolar lavage fluid containing resident macrophages, which was also abrogated by Lipo-hACE2 treatment (Figure 5).

**Figure 6:**
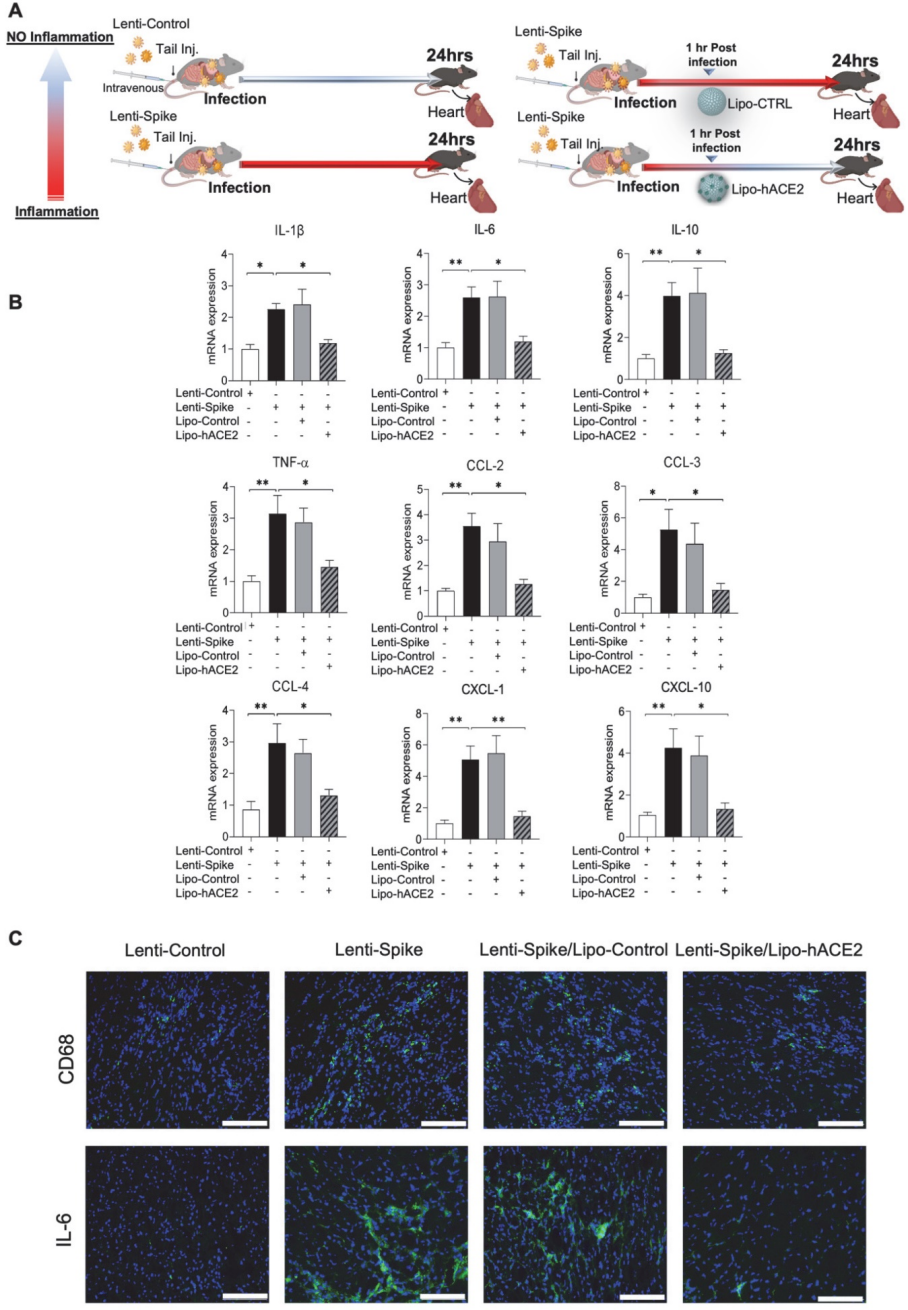
** SARS-CoV-2 Spike protein-induced cardiac inflammation is attenuated by Liposome-hACE2 complex *in vivo*. (A)** Eight-week-old male WT mice were injected IV with 100 µL of Lenti-spike (1.0x10^6^ pfu/mL) or control lentivirus followed by IV injection of 100 µL control liposome or Lipo-hACE2 (4 nM) 1 h later. After 24 h, mice were euthanized, and hearts were collected for analysis. **(B)** Total RNA was extracted from the heart, and the expression of inflammatory cytokines and chemokines were analyzed by QPCR (n = 6; *P < 0.01, **P < 0.01 and ***P < 0.001). **(C)** Representative images of immunofluorescence staining of macrophage marker CD68 (top) and IL-6 (bottom) in the heart of WT mice. The nuclei were visualized with DAPI (blue) (scale bar, 100 µm).

As a corollary, Lenti-Spike showed a positive immunostaining for macrophage marker CD68 and proinflammatory cytokine IL-6 in the lung alveoli (Figure 5C), which was blocked by Lipo-hACE2 (Figure 5D). In addition to lung inflammation or injury, studies have demonstrated that approximately 15% of COVID-19 patients with pre-existing conditions may develop acute cardiac arrhythmia and myocarditis, and macrophages may also play an important role in this process 15, 38-43. We found that Lenti-Spike also increased the myocardial inflammatory gene expressions, which were blocked by Lipo-hACE2 (Figure 6). Immunofluorescence staining revealed prominent CD68 for macrophage infiltration and IL-6 immunostaining, which were mitigated by Lipo-hACE2 (Figure 6C). Taken together, Lipo-hACE2 decoy effectively inhibited Lenti-Spike-elicited macrophage, pulmonary, and myocardial inflammation *in vivo*.

### Lipo-hACE2 inhibits Lenti-Spike or SARS-CoV-2-elicited inflammation in human macrophages

**Figure 7:**
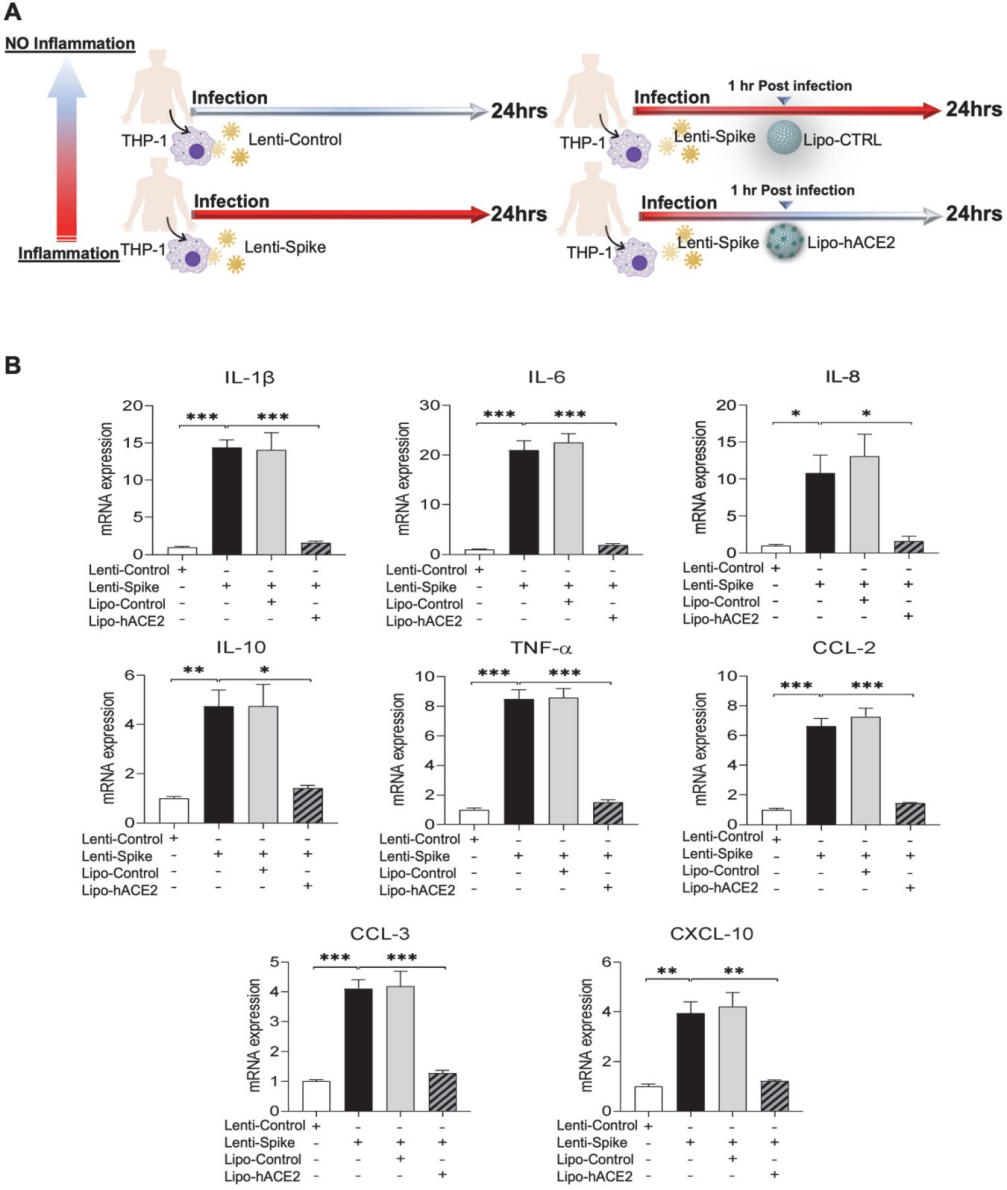
** Liposome-hACE2 suppresses SARS-CoV-2 Spike protein-induced inflammatory responses in human THP-1 macrophages. (A)** Human THP‐1 monocytes were differentiated into macrophages by exposure to 100 ng/mL of phorbol‐12‐myristate‐13‐acetate (PMA) for 48 h. The differentiated macrophages were infected with Lentivirus expressing Spike protein (Lenti-Spike) (2.3x10^7^ pfu/mL) or control lentivirus for 1 h followed by treatment with 25 nM Lipo-hACE2 or control liposome for another 23 h. **(B)** Total RNA was extracted, and the expressions of inflammatory cytokines and chemokines were analyzed by QPCR (n = 3; *P < 0.05, **P < 0.01 and ***P < 0.001).

**Figure 8:**
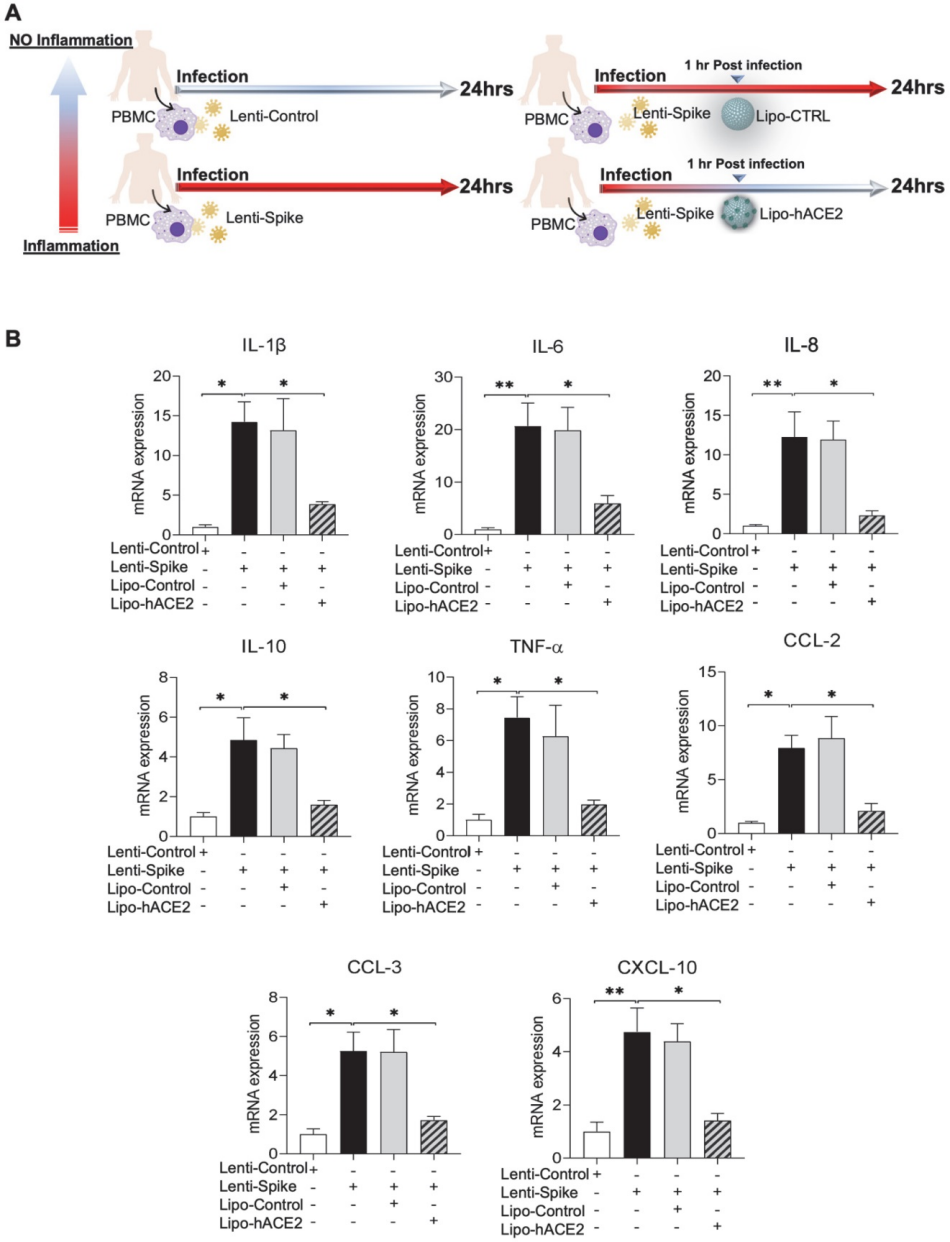

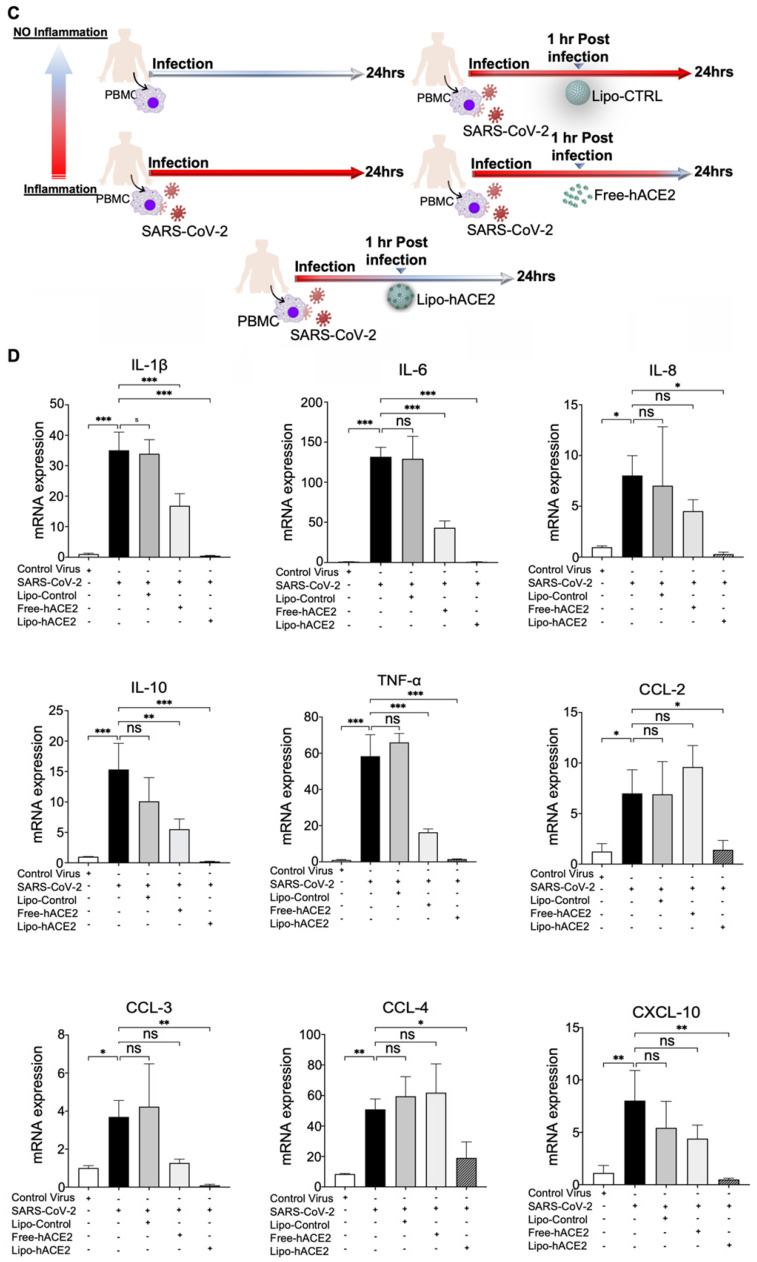
** SARS-CoV-2 and Spike protein-induced inflammatory responses are blocked by Liposome-hACE2 complex in human peripheral blood mononuclear cells. (A)** Human peripheral blood mononuclear cells (PBMC) were infected with Lentivirus expressing Spike protein (Lenti-Spike) (2.3x10^7^ pfu/mL) or control lentivirus for 1 h followed by treatment with 25 nM Lipo-hACE2 or control liposome for another 23 h. **(B)** Total RNA was extracted, and the expressions of inflammatory cytokines and chemokines were analyzed by QPCR (n = 3, *P < 0.05 and **P < 0.01). **(C)** Human PBMC were infected with SARS-CoV-2 (2.5x10^5^ pfu/mL) or inactivated SARS-CoV-2 (control virus) for 1 h followed by treatment with control liposome, 25 nM Lipo-hACE2, or 25 nM free-hACE2 for another 23 h. **(D)** Total RNA was extracted, and the expressions of inflammatory cytokines and chemokines were analyzed by QPCR (n = 3; *P < 0.05, **P < 0.01 and ***P < 0.001).

To confirm the findings obtained from murine macrophages and mouse models, differentiated human THP-1 macrophages were infected with Lenti-spike or SARS-CoV-2, followed by Lipo-hACE2 treatment (Figure 7A). Lenti-Spike significantly increased the THP-1 inflammatory genes, which were abolished by Lipo-hACE2 (Figure 7B). These findings were recapitulated in the human peripheral blood mononuclear cells (PBMC) (Figure 8C), in which SARS-CoV-2-induced inflammatory genes were significantly mitigated by Lipo-hACE2 (Figure 8D). Further, PBMC infected with SARS-CoV-2 were also treated with free-hACE2 and the anti-inflammatory effects of free-hACE2 were also evaluated. We found that Lipo-hACE2 had better inhibitory effects on SARS-CoV-2-stimulated expression of pro-inflammatory genes including IL-1β, IL-6, and TNFα as compared with free-ACE2 (Figure 8D). In addition, free-hACE2 was not able to significantly inhibit SARS-CoV-2-induced expression of several key genes including CCL2, CCL4, and CXCL-10, but Lipo-hACE2 efficiently inhibited their gene expression. Collectively, these findings support Lipo-hACE2 as a better decoy to abrogate Spike protein- or SARS-CoV-2-initiated macrophage inflammatory responses in human macrophages.

### SARS-CoV-2 spike protein induces inflammation through IKKβ signaling pathway in macrophages

**Figure 9:**
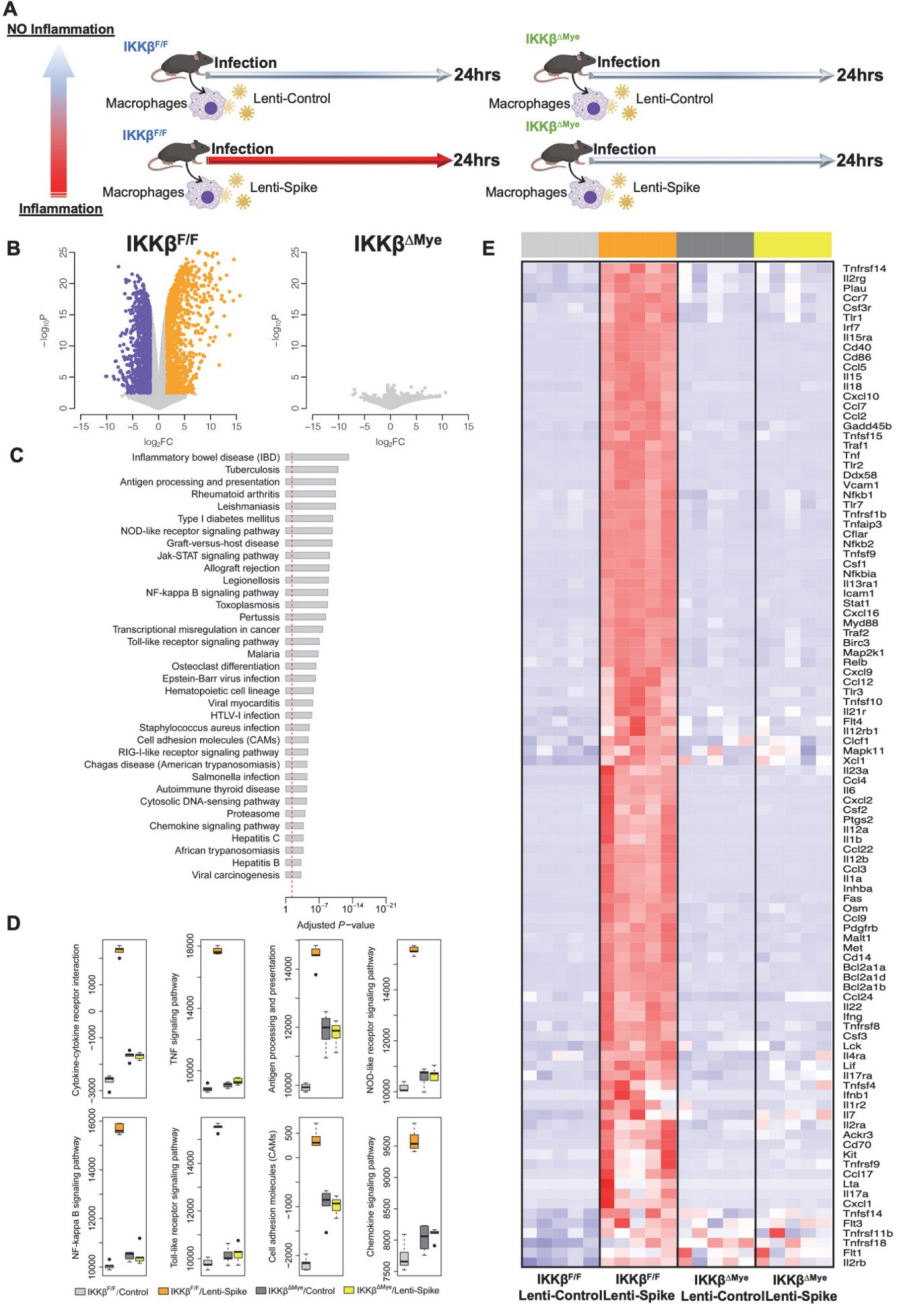
** SARS-CoV-2 Spike protein induces macrophage inflammatory responses through IKKβ signaling *in vitro*. (A)** Peritoneal macrophages were isolated from eight-week-old male IKKβ^F/F^ and IKKβ^ΔMye^ mice. Macrophages were infected with Lentivirus expressing SARS-CoV-2 Spike protein (Lenti-Spike) (2.3x10^7^ pfu/mL) or control lentivirus for 24 h. Total RNA was isolated for RNAseq analysis (n = 5). **(B)** Volcano plot of differential expression between Lenti-Spike and control treatment in macrophages of IKKβ^F/F^ and IKKβ^ΔMye^ mice. Colored dots represent the enriched (orange dots) or depleted (blue dots) differentially expressed genes (DEGs) with a false discovery rate (*FDR*) of < 1% and a fold change (*FC*) >3 as a cut-off threshold. **(C)** KEGG pathways significantly associated with the DEGs in control macrophages after Lenti-Spike infection. The *P*-values were computed by *Fisher*'s exact test. The vertical dash line indicates the significance level of *α* = 0.01. The y-axis displays the KEGG pathways while the x-axis displays the *P*-values. **(D)** Geneset scores of the prioritized KEGG pathways. The geneset score was calculated using the FAIME algorithm. **(E)** Heatmap representation of DEGs involved in the pathways of “cytokine-cytokine receptor interaction”, “TNF signaling pathway”, “Antigen processing and presentation”, “NOB-like receptor signaling pathway”, “NF-kappa B signaling pathway”, and “Toll-like receptor signaling pathway”, “Cell adhesion molecules”, and “Chemokine signaling pathway”. Each row shows one individual gene and each column a biological replicate of mouse. Red represents relatively increased gene expression while blue denotes downregulation.

To elucidate IKKβ/NF-κB signaling as the molecular mechanism underlying Spike protein-mediated macrophage inflammation 15, peritoneal macrophages were isolated from myeloid-specific IKKβ-deficient (IKKβ^ΔMye^) and control IKKβ-flox (IKKβ^F/F^) mice, and infected control lentivirus or lentivirus expressing Spike protein (Figure 9A) 22. RNA-Seq analysis revealed 2,362 differentially expressed genes (DEGs) from the macrophages of IKKβ^F/F^ mice with a false discovery rate (*FDR*) of < 1% and a fold change (*FC*) >3 as a cut-off threshold (Figure 9B and Supplemental Table 2). No DEGs were identified in the IKKβ-deficient macrophages using the same threshold (Figure 9B). KEGG analysis further showed that these DEGs were enriched in immune responses as “cytokine-cytokine receptor interaction”, “NF-κB, TNF-α”, and “Toll-like receptor signaling pathways” (Figure 9C). By using the *FAIME* algorithm 22, we corroborated the higher geneset scores of these pathways in the Lenti-Spike-infected macrophages as compared with the cells infected with control virus in macrophages of IKKβ^F/F^ mice (Figure 9D). IKKβ deletion resulted in reduced geneset scores of the same pathways in the macrophages of IKKβ^ΔMye^ mice (Figure 9D). Consistent with the *FAIME* analysis, the DEGs associated with these pathways in macrophages of IKKβ^F/F^ mice were upregulated by Lenti-Spike treatment, whereas deficiency in IKKβ abolished the Spike-induced gene expression (Figure 9E).

In addition to KEGG analysis, we further performed gene ontology analysis using the definition from the GO 33. Consistent with KEGG analysis, the DEGs were enriched in several inflammation-associated biological processes including immune system process and inflammatory response (Supplemental Figure 1A). FAIME analysis also demonstrated higher geneset scores of those GO terms in Lenti-Spike-infected macrophages from IKKβ^F/F^LDLR^-/-^ mice compared with the controls, but deficiency of IKKβ abolished Lenti-Spike-elevated geneset scores in the macrophages of IKKβ^ΔMye^ LDLR^-/-^ mice (Supplemental Figure 1B). For example, IKKβ ablation suppressed the Lenti-Spike-induced geneset scores of immune system process, immune response, and inflammatory response in the IKKβ-deficient macrophages (Supplemental Figure 1B). Further, DEGs associated with these three pathways were upregulated in Lenti-Spike-infected macrophages of IKKβ^F/F^ mice, and IKKβ deficiency abolished Spike protein-stimulate gene expression. Collectively, IKKβ/NF-κB signaling is implicated in the SARS-CoV-2 Spike-mediated macrophage inflammatory responses.

## Discussion

SARS-CoV-2 variants were reported to evade COVID-19 vaccines and to escape neutralization by antibodies in recovered COVID-19 patients 44, 45 and there remains a clinical challenge to identify therapeutic targets for SARS-CoV-2-induced inflammation in the macrophages, which are the frontline immune cells in the respiratory system. Accumulating evidence suggest that the primary target for SARS-CoV-2 is the respiratory tract 46, 47 where the virus is still free to mutate and replicate even after receiving vaccination 48. To this end, the novelty of our studies resides in engineering liposomes conjugated with human-ACE2, Lipo-hACE2 as a decoy (d = 100 nm) to inhibit cytokine and chemokine production in macrophages. In addition to SARS-CoV-2, we cloned and inserted the Spike sequence into the lentiviruses as pseudo-SARS-CoV-2. We then used Lipo-hACE2 to target macrophage-mediated inflammation following SARS-CoV-2 or Lenti-Spike infection and found significantly decreased inflammatory response in both murine and human macrophages. SARS-CoV-2 or Lenti-Spike induced a host of macrophage cytokine expressions, including IL-1β, IL-6, TNFα, CCL-2, -3, -4, and CXCL-10, whereas Lipo-hACE2 decoy nearly abolished these effects in macrophages isolated from WT mice, comparable to what was observed in human THP1 and PBMC.

SARS-CoV-2 is well-recognized to promote the dysregulated immune responses in patients with severe COVID-19 49. As the first line of defense, macrophages contribute to SARS-CoV-2-induced inflammation 5, 7, 15, 50. SARS-CoV-2 uptake is normally mediated by the interaction between hACE2, and SARS-CoV-2 spike protein. However, previous studies found no significant difference in viral load uptake between macrophages isolated from WT and hACE2 transgenic mice 7, 15. Further, knockdown of hACE2 did not decrease the uptake of the virus by the macrophages 12. While these studies suggest that SARS-CoV-2 can hijack macrophages and induce inflammatory responses in an hACE2-independent manner, there remains a paucity of studies to elucidate the mechanisms underlying SARS-CoV-2-induced macrophage inflammation. While the Spike protein does not bind to the murine ACE2, we demonstrated that SARS-CoV-2 induced the expression of cytokines and chemokines in both murine and human macrophages, indicating hACE2-independent mechanisms, possibly through phagocytosis or mechanical softness and membrane deformability of macrophages 12. Collectively, our results suggest that SARS-CoV-2 may infect macrophages through both hACE2-dependent and -independent mechanisms. In the presence of hACE2, Lipo-hACE2 may block the interaction between SARS-CoV-2 spike protein and hACE2 receptors. In the absence of hACE2, SARS-CoV-2 can still bind to hACE2 on the surface of the liposomes which prevents the virus from priming macrophage-mediated inflammation. In addition, previous studies have also suggested that macrophages can uptake similar liposomal-based nanoparticles through phagocytosis 51, so it is also plausible that Lipo-hACE2 (with or without SARS-CoV-2 binding) can be internalized by macrophages through phagocytosis, leading to inhibitory effects on SARS-CoV-2 viral particle replication, which contributes to decreased inflammatory responses against SARS-CoV-2-induced inflammatory responses. Future studies are required to further investigate the detailed mechanisms through which Lipo-hACE2 strongly inhibit SARS-CoV-2-induced macrophage inflammation.

Neutralizing SARS-COV-2 by hACE2 decoys has been proposed as a therapeutic target to ameliorate SARS-CoV-2-stimulated inflammation 11, 46, 51-59. For example, ACE2 nanodecoys derived from human lung spheroid cells have been shown to neutralize SARS-CoV-2 and protect non-human primates from SARS-CoV-2-induced lung injury 60. We found that our engineered Lipo-hACE2 inhibited Lenti-Spike-induced inflammatory gene expressions in peritoneal macrophages and bronchoalveolar lavage fluid in mice. Furthermore, exposure to Spike protein led to increased macrophage infiltration, elevated IL-6 protein levels, and upregulation of key inflammatory gene expression in the lungs and myocardium, which were abrogated by the Lipo-hACE2 decoy. We also found that Lipo-hACE2 particles engendered neutralizing effects against SARS-CoV-2-induced cell death or macrophage inflammation to a greater extent than the previously described free hACE2 proteins 61 at a given concentration, potentially due to the size of nano-liposomes (d = 100 nm) which may be implicated in the physical inhibition of the viral binding to the hACE2. In addition to directly neutralizing SARS-CoV-2, Lipo-hACE2 particles may also inhibit SARS-CoV-2-induced inflammatory cytokines by absorbing those cytokines, leading to a protective effect for alveolar epithelial cells which was also observed in another study by Wang *et al.* 62.

We further assessed the potential molecular mechanisms underpinning SARS-CoV-2-mediated macrophage inflammation. The severity of COVID-19 is associated with the SARS-CoV-2 load, and the activation of host inflammatory responses. For this reason, mitigating the inflammatory response is critical to develop anti-SARS-CoV-2 therapies 63. NF-κB signaling has been implicated as a key pathway underlying COVID-19-associated inflammation 15, and we recently revealed the important role of IKKβ, a central inflammatory coordinator through activating NF-κB, in the regulation of other viral protein (e.g. HIV Tat)-induced macrophage inflammation and vascular disease 22. In the present study, we found that Spike protein induced macrophage inflammatory responses in an IKKβ-dependent manner. Exposure to Spike protein led to > 2,000 DEGs in control macrophages associated with key inflammatory pathways, including “NF-κB and TNF-α signaling” and “Toll-like receptor (TLR) signaling”. In contrast, deficiency in IKKβ nearly abrogated Spike protein-associated geneset scores and DEG-associated key inflammatory pathways. These results implicate IKKβ as a key molecular switch essential for SARS-CoV-2-induced macrophage inflammatory responses. Furthermore, our results showed that the deficiency of IKKβ led to the inhibition of Lenti-Spike-stimulated TLR signaling activation. TLR signaling is responsible for promoting many inflammatory stimuli-mediated activation of canonical NF-κB pathway 64. Our results suggest that IKKβ may act as a positive regulator of TLR signaling, thus indicating a potential therapeutic target for inflammation diseases.

Whilst SARS-CoV-2 infects primarily the respiratory system, it has a tropism for the cardiovascular system, leading to arrhythmias and myocarditis 42, 65. While the underlying mechanism is not fully understood, non-resident immune cells, including macrophages, have been reported to mediate cardiac inflammatory responses 66-68. In our study, Spike protein promoted macrophage infiltration and IL-6 expression in the myocardium, which was attenuated by Lipo-hACE2 decoy. Further investigation is warranted to elucidate the distinct immune cell types underlying SARS-CoV-2-induced arrhythmias and myocarditis, and the implementation of Lipo-hACE2 decoy as a countermeasure for clinical translation.

In summary, we established that engineered Lipo-hACE2 as a decoy efficiently abrogated SARS-CoV-2 or Spike protein-mediated macrophage inflammatory responses, and IKKβ/NF-κB signaling pathway is implicated in SARS-CoV-2-stimulated inflammation. We demonstrated that SARS-CoV-2 induced the expression of cytokines and chemokines in both murine and human macrophages, suggesting a non-hACE2 mechanism to activate IKKβ/NF-κB signaling. Taken together, engineering Lipo-hACE2 offers a therapeutic target to mitigate by myeloid-derived immune cells from priming cytokine storm amid severe SARS-CoV-2 infection.

## Supplementary Material

Supplementary figure and tables.
