# Supplementary material for: An engineered nano-liposome-human ACE2 decoy neutralizes SARS-CoV-2 Spike protein-induced inflammation in both murine and human macrophages
Source: Theranostics. 2022 Mar 6;12(6):2639–57. doi: 10.7150/thno.66831 (PMC8965482; doi:10.7150/thno.66831)
Supplement: Supplementary file 1 — Supplementary figure and tables. [file thnov12p2639s1.pdf]

Supplemental Material for

An engineered nano-liposome-human ACE2 decoy neutralizes SARS-CoV-2 Spike protein-induced inflammation in both murine and human macrophages

Sandro Satta[1], Zhaojie Meng[2], Rebecca Hernandaz[2], Susana Cavallero[1],Tong Zhou[3], Tzung K. Hsiai[1,4], and Changcheng Zhou[2]

[1]Department of Medicine, David Geffen School of Medicine, University of California, Los Angeles, CA
[2]Division of Biomedical Sciences, School of Medicine, University of California, Riverside, CA; [3]Department of Physiology and Cell Biology, Reno School of Medicine, University of Nevada, Reno, NV
[4]Department of Bioengineering, School of Engineering & Applied Science, University of California, Los Angeles, CA

This PDF file includes:

Supplemental Figure 1
Supplemental Table 1-2

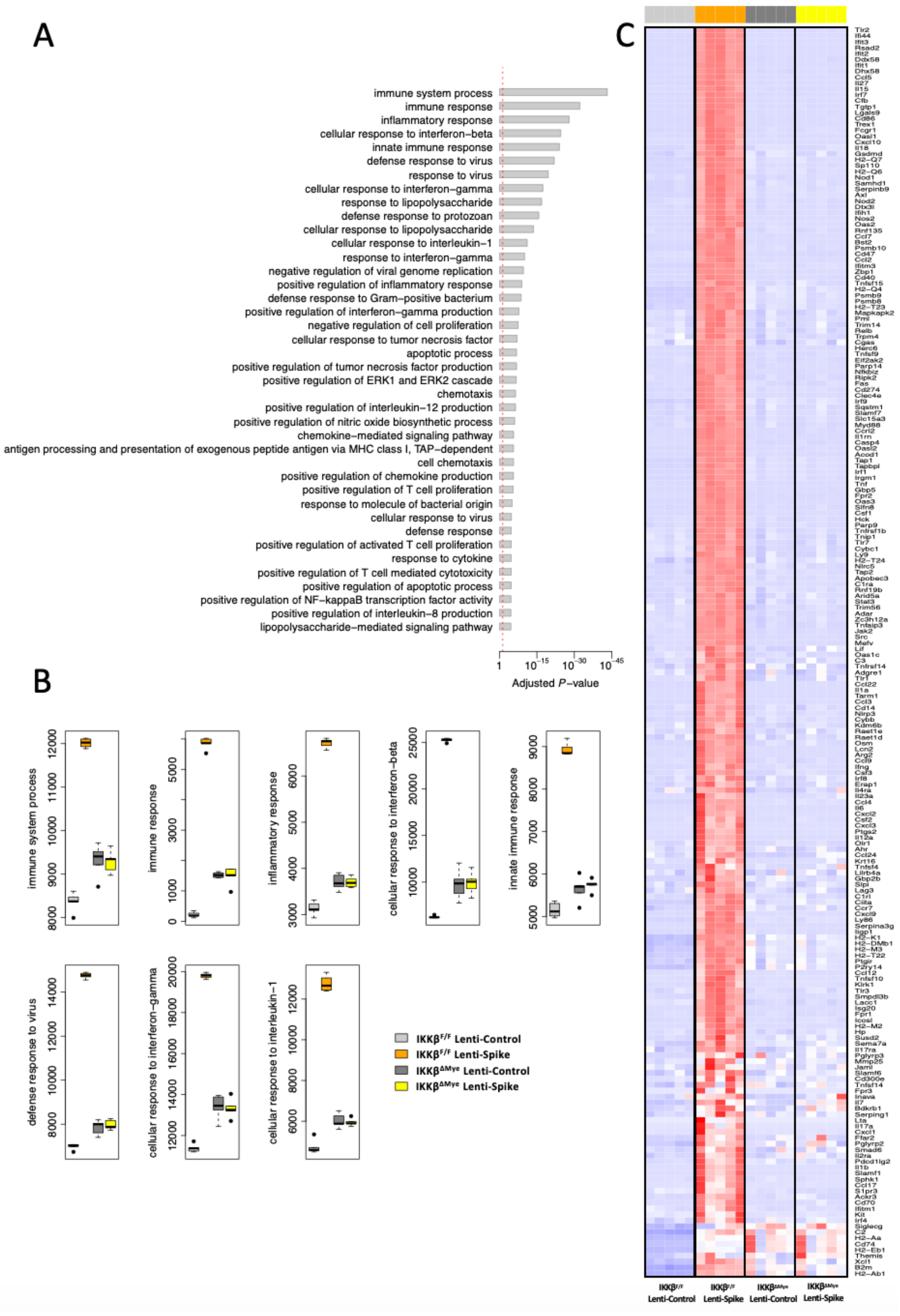
A
immune system process
immune response
inflammatory response
cellular response to interferon−beta
innate immune response
defense response to virus
response to virus
cellular response to interferon−gamma
response to lipopolysaccharide
defense response to protozoan
cellular response to lipopolysaccharide
cellular response to interleukin−1
response to interferon−gamma
negative regulation of viral genome replication
positive regulation of inflammatory response
defense response to Gram−positive bacterium
positive regulation of interferon−gamma production
negative regulation of cell proliferation
cellular response to tumor necrosis factor
apoptotic process
positive regulation of tumor necrosis factor production
positive regulation of ERK1 and ERK2 cascade
chemotaxis
positive regulation of interleukin−12 production
positive regulation of nitric oxide biosynthetic process
chemokine−mediated signaling pathway
antigen processing and presentation of exogenous peptide antigen via MHC class I, TAP−dependent
cell chemotaxis
positive regulation of chemokine production
positive regulation of T cell proliferation
response to molecule of bacterial origin
cellular response to virus
defense response
positive regulation of activated T cell proliferation
response to cytokine
positive regulation of T cell mediated cytotoxicity
positive regulation of apoptotic process
positive regulation of NF−kappaB transcription factor activity
positive regulation of interleukin−8 production
lipopolysaccharide−mediated signaling pathway
1
10−15
10−30
10−45
Adjusted P−value
B
immune system process
immune response
inflammatory response
cellular response to interferon−beta
innate immune response
defense response to virus
cellular response to interferon−gamma
cellular response to interleukin−1
IKKβF/F Lenti-Control
IKKβF/F Lenti-Spike
IKKβΔMye Lenti-Control
IKKβΔMye Lenti-Spike
C
IKKβF/F Lenti-Control
IKKβF/F Lenti-Spike
IKKβΔMye Lenti-Control
IKKβΔMye Lenti-Spike

Supplemental Figure 1. Gene Ontology (GO) Biological Process analysis of RNA-Seq results from macrophages infected with control lentivirus or lentivirus expressing SARS-CoV-2 Spike protein.
Peritoneal macrophages were isolated from eight-week-old male $IKK\beta^{F/F}$ and $IKK\beta^{\Delta Mye}$ mice. Macrophages were infected with Lentivirus expressing SARS-CoV-2 Spike protein (Lenti-Spike) ($2.3x10^7$ pfu/mL) or control lentivirus for 24 hr. Total RNA was isolated for RNAseq analysis (n=5). (A) Gene Ontology (GO) Biological Process terms significantly associated with the DEGs in control macrophages after Lenti-Spike infection. The *P*-values were computed by *Fisher*'s exact test. The vertical dash line indicates the significance level of α=0.01. The y-axis displays the GO Biological Process terms while the x-axis displays the *P*-values. (B) Geneset scores of the prioritized GO terms. The geneset score was calculated using the FAIME algorithm. (C) Heatmap representation of DEGs involved in the biological processes of "immune system process", "immune response", and "inflammatory response" shown in panel A and B. Each row shows one individual gene and each column a biological replicate of mouse. Red represents relatively increased gene expression while blue denotes downregulation.

Supplemental Table 1. Primer sequences for QPCR

**Mouse Primers**	F<br>R	**Human Primers**	F<br>R
CCL-2	5'- TTAAAAACCTGGATCGGAACCAA -3'<br>5'- GCATTAGCTTCAGATTTACGGGT -3'	CCL-2	5'-TGTCCCAAAGAAGCTGTGATCT-3'<br>5'-GGAATCCTGAACCCACTTCTG-3'
CCL-3	5'-TTCTCTGTACCATGACACTCTGC-3'<br>5'-CGTGGAATCTTCCGGCTGTAG-3'	CCL-3	5'-CGCCTGCTGCTTCAGCTACACCTCCCGGCAGA-3'<br>5'-TGGACCCCTCAGGCACTCAGCTCCAGGTCGCT-3'
CCL-4	5'-TTCCTGCTGTTTCTCTTACACCT-3'<br>5'-CTGTCTGCCTCTTTTGGTCAG-3'	CXCL-10	5'- GTGGCATTCAAGGAGTACCTC -3'<br>5'- TGATGGCCTTCGATTCTGGATT -3'
CXCL-1	5'- CCGAAGTCATAGCCACACTCAA -3'<br>5'- GCAGTCTGTCTTCTTTCTCCGTTAC-3'	IL-1β	5'- CGCCAATGACTCAGAGGAAGA -3'<br>5'- AGGGCGTCATTCAGGATGAA -3'
CXCL-10	5'- CCAAGTGCTGCCGTCATTTTC -3'<br>5'- GGCTCGCAGGGATGATTTCAA -3'	IL-6	5'- GGTACATCCTCGACGGCATCT -3'<br>5'- GTGCCTCTTTGCTGCTTTCAC -3'
IL-1β	5'-GCAACTGTTCCTGAACTCAACT-3'<br>5'-ATCTTTTGGGGTCCGTCAACT-3'	IL-8	5'- TTTTGCCAAGGAGTGCTAAAGA -3'<br>5'- AACCCTCTGCACCCAGTTTTC -3'
IL-6	5'-CCGAGGAAGACGTGGACACCTTC-3'<br>5'-CCTCAGCCATGACCTGCCTTGTAG-3'	IL-10	5'- TCAAGGCGCATGTGAACTCC -3'<br>5'- GATGTCAAACTCACTCATGGCT -3'
IL-10	5'- GCTCTTACTGACTGGCATGAG -3'<br>5'- CGCAGCTCTAGGAGCATGTG -3'	TNFα	5'- CCTCTCTCTAATCAGCCCTCTG -3'<br>5'- GAGGACCTGGGAGTAGATGAG -3'
TNFα	5'- CCCATATACCTGGGAGGAGTCTTC -3'<br>5'- CATTCCCTTCACAGAGCAATGAC -3'	GAPDH	5'-GGCCTCCAAGGAGTAAGACC-3'<br>5'-AGGGGAGATTCAGTGTGGTG-3'
GAPDH	5'-AACTTTGGCATTGTGGAAGG-3'<br>5'-GGATGCAGGGATGATGTTCT-3'		

Supplemental Table 2. Detailed RNAseq data of macrophage DEGs induced by Lenti-Spike

Gene	log2 (FC)	P-value	FDR	FC	Gene	log2 (FC)	P-value	FDR	FC
**Nos2**	15.66551162	8.75E-19	6.97E-17	51974.32958	**Ccl12**	8.415747679	9.01E-13	1.49E-11	341.5013991
**Ifi44**	14.5174660	1.37E-24	2.97E-21	23452.69362	**Slco4a1**	8.31434401	1.32E-13	2.58E-12	318.32221
**Il1b**	14.4098103	5.40E-18	3.25E-16	21766.32752	**Alpk2**	8.308008346	1.60E-13	3.08E-12	316.9273478
**Slamf1**	13.7048968	1.74E-12	2.68E-11	13353.1905	**Thbs4**	8.283955283	2.59E-08	1.73E-07	311.6872398
**Serpina3f**	13.6655515	1.93E-19	2.04E-17	12993.94174	**Il27**	8.219949036	2.45E-20	3.40E-18	298.1612652
**Il12b**	13.11365637	4.59E-22	1.51E-19	8863.472852	**Gm4951**	8.207123877	7.90E-18	4.47E-16	295.5224408
**Cxcl9**	12.6948834	8.09E-18	4.57E-16	6630.415081	**Ch25h**	8.066520773	1.31E-15	4.21E-14	268.0801843
**Lad1**	12.6475602	1.60E-15	4.98E-14	6415.099901	**Carlr**	8.06002624	4.40E-08	2.82E-07	266.8760887
**Il6**	12.2073161	8.70E-19	6.96E-17	4728.9891	**Olfr433**	8.003990266	9.93E-12	1.30E-10	256.7090355
**Il12a**	12.1396058	3.29E-18	2.16E-16	4512.170344	**Tnf**	7.96398126	1.35E-18	9.99E-17	249.6877548
**Gbp5**	11.89864057	1.84E-21	4.39E-19	3818.103687	**Ccl17**	7.883820313	1.96E-09	1.61E-08	236.1926585
**Rnf225**	11.81063296	1.43E-21	3.52E-19	3592.152692	**H2-M2**	7.866148632	6.20E-17	2.78E-15	233.3171638
**Lta**	11.68842859	1.13E-07	6.71E-07	3300.407826	**Gbp3**	7.853774263	7.18E-26	7.25E-22	231.3245
**Ms4a4c**	11.54378672	5.05E-24	8.42E-21	2985.561806	**Irf7**	7.801842202	1.26E-21	3.17E-19	223.1457009
**Ifit3b**	11.31436526	1.09E-19	1.25E-17	2546.615195	**Calhm6**	7.798991259	5.02E-23	3.40E-20	222.7051729
**Ifi206**	11.28326021	1.67E-25	7.25E-22	2492.296868	**Il23a**	7.796402648	3.13E-07	1.73E-06	222.3059339
**Igf2bp1**	11.2180605	8.46E-14	1.74E-12	2382.169736	**AA467197**	7.792717605	6.34E-23	4.04E-20	221.7388276
**Tarm1**	11.0339453	1.85E-20	2.72E-18	2096.75895	**Pla1a**	7.785847588	8.53E-13	1.42E-11	220.6854322
**Il22**	10.8703029	3.69E-12	5.31E-11	1871.919854	**Six1**	7.732198437	4.24E-07	2.29E-06	212.6295705
**Gbp10**	10.5521997	1.76E-11	2.17E-10	1501.511392	**Oas1g**	7.64070379	5.40E-16	1.92E-14	199.5634616
**Acod1**	10.5495915	1.33E-25	7.25E-22	1498.799352	**Gbp2**	7.638380113	1.29E-25	7.25E-22	199.2422935
**Cxcl10**	10.52171108	6.26E-20	7.71E-18	1470.112735	**Rsad2**	7.624599548	1.06E-22	5.25E-20	197.3481997
**Edn1**	10.4105287	4.88E-16	1.75E-14	1361.073044	**Car2**	7.603815233	3.74E-14	8.27E-13	194.5254671
**Ifit3**	10.3927760	4.64E-25	1.26E-21	1344.427306	**Cmpk2**	7.595044939	7.30E-20	8.94E-18	193.3465134
**Apol9b**	10.3137642	3.55E-18	2.28E-16	1272.77722	**Gm16181**	7.49796545	3.96E-13	7.10E-12	180.7642347
**Ifit2**	10.3022166	3.26E-23	2.52E-20	1262.630343	**Gpr84**	7.40862336	3.84E-17	1.83E-15	169.9095767
**Tgtp2**	10.2966990	3.56E-25	1.10E-21	1257.810641	**Rasgrp1**	7.364448543	5.36E-19	4.54E-17	164.7858486
**Iigp1**	10.2130742	1.68E-21	4.06E-19	1186.975336	**Zbp1**	7.310833578	1.67E-22	7.48E-20	158.7742977
**Ptgs2**	10.1393583	1.30E-18	9.77E-17	1127.849117	**Il1r2**	7.307944314	1.23E-05	5.21E-05	158.4566409
**Ccl5**	10.1318043	7.99E-23	4.33E-20	1121.959046	**H2-Q6**	7.294877549	3.14E-20	4.21E-18	157.0279485
**Trim30c**	10.11866613	1.62E-21	3.95E-19	1111.788103	**Apobec4**	7.275531894	3.17E-06	1.48E-05	154.936355
**Apol9a**	10.0473177	9.47E-16	3.12E-14	1058.142178	**Serpine1**	7.268500156	4.24E-18	2.63E-16	154.183028
**Klri1**	9.98993682	4.65E-12	6.56E-11	1016.882189	**Clec4e**	7.236072375	8.92E-21	1.53E-18	150.7560797
**Ifi213**	9.96413835	4.74E-22	1.51E-19	998.8597765	**Cd69**	7.212439793	1.23E-20	2.02E-18	148.3066823
**Hcar2**	9.91469801	5.18E-21	9.60E-19	965.2093053	**Syt7**	7.186161286	4.35E-13	7.72E-12	145.6297489
**Gbp4**	9.89808753	9.39E-17	4.02E-15	954.1600898	**Misp**	7.176229692	2.40E-09	1.93E-08	144.6306683
**Ifi205**	9.80870599	2.74E-22	1.06E-19	896.839492	**Klri2**	7.153874539	5.48E-14	1.18E-12	142.4068307
**Cxcl2**	9.80473335	1.05E-18	8.10E-17	894.3733288	**Rtp4**	7.125061107	1.77E-23	1.95E-20	139.5909022
**Heatr9**	9.68665334	7.04E-11	7.70E-10	824.0872858	**Klrk1**	7.112704755	7.82E-13	1.32E-11	138.4004434
**Lcn2**	9.64905443	1.36E-21	3.38E-19	803.3614605	**Oas3**	7.083486602	6.54E-22	1.89E-19	135.6256838
**Inhba**	9.63565715	1.91E-20	2.74E-18	795.4663861	**Slfn4**	7.082152375	3.35E-18	2.18E-16	135.5003131
**Ifit1bl1**	9.59135343	5.93E-15	1.57E-13	771.4096788	**H2-Q7**	7.040944953	1.67E-19	1.81E-17	131.684794
**Ccl22**	9.55067831	2.87E-19	2.77E-17	749.9644052	**Usp18**	7.008747689	8.67E-22	2.35E-19	128.7784776
**Ifit1**	9.52489981	3.01E-25	1.09E-21	736.6828313	**Isg20**	6.986440264	4.46E-19	3.96E-17	126.8025777
**Lhx2**	9.47638749	4.00E-15	1.10E-13	712.3228652	**Il1a**	6.97799173	1.86E-20	2.72E-18	126.0621821
**Cish**	9.42563912	1.40E-14	3.43E-13	687.7017129	**Gbp9**	6.825402985	4.95E-22	1.55E-19	113.4099166
**Ifi208**	9.30799159	5.95E-18	3.51E-16	633.8473368	**Ppm1n**	6.82454363	8.45E-06	3.67E-05	113.342383
**Cxcl3**	9.21735841	1.73E-15	5.35E-14	595.2526873	**Aqp3**	6.789865174	1.31E-05	5.49E-05	110.6504244
**Serpina3g**	9.20755761	5.55E-18	3.31E-16	591.2226039	**Gm19410**	6.721668936	6.38E-06	2.82E-05	105.5416721
**Cd40**	9.10006325	1.52E-25	7.25E-22	548.7720714	**Cd300ld5**	6.717792334	0.00067180	0.002146	105.2584564
**Gbp6**	9.07251845	8.00E-22	2.25E-19	538.3939734	**Gm15056**	6.710253422	4.84E-14	1.06E-12	104.7098551
**Fpr2**	9.07005092	1.14E-23	1.55E-20	537.4739118	**Slc7a2**	6.688659644	2.94E-21	6.01E-19	103.1542637
**Slfn1**	9.002112369	6.06E-24	9.38E-21	512.7502105	**Arg2**	6.686795515	5.37E-16	1.91E-14	103.0210626
**Ccl2**	8.97235106	2.70E-21	5.73E-19	502.2810615	**Zfp811**	6.653187701	6.22E-09	4.62E-08	100.6489074
**Ifng**	8.95381838	3.09E-13	5.64E-12	495.870084	**Pilra**	6.644819488	2.52E-21	5.69E-19	100.0667931
**Isg15**	8.84437315	4.44E-22	1.48E-19	459.6440169	**Oasl1**	6.643292764	1.37E-20	2.15E-18	99.96095392
**Gbp2b**	8.83656201	4.80E-09	3.65E-08	457.1621048	**Xaf1**	6.629586055	3.83E-20	5.07E-18	99.01574546
**Cst7**	8.81215277	1.68E-18	1.21E-16	449.4923566	**Vnn3**	6.608785099	3.02E-24	5.96E-21	97.59836669
**Upp1**	8.7833227	4.97E-19	4.27E-17	440.5990885	**Ly6i**	6.599261826	1.41E-09	1.19E-08	96.95623863
**Ccl7**	8.747109116	2.45E-20	3.40E-18	429.677111	**Ccdc17**	6.587566555	0.0026304	0.007650	96.17343578
**Magee2**	8.60659970	4.14E-09	3.19E-08	389.8025455	**Csf2**	6.560418876	4.97E-08	3.15E-07	94.38062865
**Bpifc**	8.59502083	3.09E-09	2.44E-08	386.6865658	**Gbp7**	6.494998565	4.69E-22	1.51E-19	90.19643864
**Ptx3**	8.59354653	9.35E-18	5.17E-16	386.2916093	**Tdgf1**	6.489519154	0.0002003	0.000699	89.85451884
**Tgtp1**	8.58068683	1.89E-20	2.72E-18	382.863638	**Batf2**	6.48799499	4.76E-20	6.21E-18	89.75964034
**Cfb**	8.535199	4.49E-18	2.77E-16	370.9804	**Htra4**	6.458503	8.74E-11	9.38E-10	87.94335

Gene	log2 (FC)	P-value	FDR	FC	Gene	log2 (FC)	P-value	FDR	FC
**Ptges**	6.453591	1.91E-23	1.95E-20	87.64444	**Fabp3**	5.601709	2.52E-19	2.51E-17	48.56042
**Slco3a1**	6.423665	2.24E-23	2.12E-20	85.84515	**Axl**	5.597385	2.59E-21	5.73E-19	48.41511
**Phf11a**	6.4203	1.78E-18	1.26E-16	85.64516	**Olr1**	5.568756	2.15E-14	5.04E-13	47.46381
**Tmem132e**	6.415422	7.08E-08	4.36E-07	85.35607	**Dnase1l3**	5.567539	6.53E-06	2.88E-05	47.4238
**Vcam1**	6.396946	2.72E-20	3.67E-18	84.26993	**Lrrc75a**	5.563949	6.44E-21	1.16E-18	47.30593
**Cd70**	6.390425	2.32E-05	9.38E-05	83.8899	**Clmp**	5.547616	2.80E-23	2.34E-20	46.77338
**Gm49358**	6.386785	0.002775	0.008043	83.67849	**Il15ra**	5.545094	1.68E-17	8.80E-16	46.69168
**Oas2**	6.375523	1.87E-20	2.72E-18	83.02785	**Scimp**	5.533417	1.84E-16	7.29E-15	46.31531
**Gm21970**	6.37203	2.12E-05	8.59E-05	82.82702	**Icosl**	5.526041	5.61E-16	1.98E-14	46.07913
**Ubd**	6.331584	1.95E-07	1.11E-06	80.53723	**Pdgfrb**	5.503552	2.31E-17	1.16E-15	45.3664
**Socs2**	6.329971	5.64E-17	2.60E-15	80.44722	**Tlr2**	5.479812	1.95E-22	8.12E-20	44.62598
**Traf1**	6.328392	1.56E-20	2.39E-18	80.35925	**Shisa3**	5.41736	1.22E-08	8.62E-08	42.73541
**Ifi47**	6.320666	7.38E-23	4.25E-20	79.93007	**Col27a1**	5.415562	5.08E-09	3.85E-08	42.68217
**Adm2**	6.305767	0.000242	0.000834	79.10886	**Oasl2**	5.40988	3.16E-22	1.12E-19	42.51441
**Ccnd2**	6.292293	1.02E-19	1.20E-17	78.37347	**Rasgef1c**	5.400552	0.000612	0.001965	42.2404
**Vmn2r124**	6.264535	0.000346	0.00116	76.87994	**Insrr**	5.383284	0.000489	0.001596	41.73785
**Ackr3**	6.235202	1.67E-07	9.66E-07	75.33258	**Cdh6**	5.375343	0.000586	0.00189	41.50874
**Trex1**	6.222661	3.12E-22	1.12E-19	74.68055	**Icam1**	5.370573	1.15E-24	2.77E-21	41.37172
**Lysmd2**	6.216524	1.47E-11	1.85E-10	74.36354	**AC167036.2**	5.357362	2.33E-06	1.11E-05	40.9946
**Olfr1271**	6.213477	0.001227	0.003766	74.20667	**Adipoq**	5.351212	0.001687	0.005069	40.82021
**Cxcl1**	6.189198	4.12E-11	4.76E-10	72.9683	**Ifi203**	5.35064	1.44E-23	1.74E-20	40.80403
**Ffar2**	6.166026	9.57E-07	4.83E-06	71.80569	**Gem**	5.346858	4.10E-18	2.55E-16	40.69721
**BC147527**	6.156982	1.19E-09	1.02E-08	71.35695	**Aqp9**	5.346454	1.36E-18	1.00E-16	40.68582
**Nts**	6.137769	7.25E-05	0.000271	70.41298	**Mlkl**	5.343907	6.03E-23	3.96E-20	40.61405
**Tcp10b**	6.096772	4.42E-09	3.38E-08	68.44018	**Oas1h**	5.329016	0.000437	0.001436	40.19702
**Ppfia3**	6.091513	2.01E-08	1.37E-07	68.19117	**Ovol1**	5.325581	2.96E-05	0.000118	40.10142
**Socs3**	6.08981	1.31E-20	2.10E-18	68.11072	**Eva1b**	5.309353	2.46E-19	2.47E-17	39.65285
**Clic5**	6.070755	3.32E-16	1.24E-14	67.21704	**Sprr2e**	5.292757	6.88E-07	3.57E-06	39.19933
**Dmwd**	6.06996	1.11E-09	9.59E-09	67.18001	**Ifi209**	5.284765	6.68E-24	9.66E-21	38.98278
**Olfr56**	6.047102	2.67E-07	1.50E-06	66.12397	**Mfsd7a**	5.277284	4.78E-20	6.21E-18	38.78115
**Majin**	6.044892	2.60E-08	1.73E-07	66.02277	**Pcp4**	5.276604	0.000571	0.001844	38.76289
**Olfr1396**	6.038738	5.26E-05	0.000201	65.74176	**Gm12216**	5.251225	8.87E-14	1.81E-12	38.08695
**Nt5c1a**	5.996998	4.30E-07	2.32E-06	63.86696	**Fam3b**	5.25059	3.59E-06	1.66E-05	38.07019
**Tnfsf10**	5.980176	3.69E-14	8.19E-13	63.12658	**Serpina11**	5.241335	0.000959	0.002994	37.82676
**Tnfsf4**	5.970484	0.000132	0.000473	62.70395	**H1f10**	5.238097	1.19E-08	8.39E-08	37.74195
**Fpr1**	5.96631	3.50E-11	4.09E-10	62.52276	**AC168977.2**	5.227561	0.002089	0.006184	37.46733
**Fpr3**	5.966045	0.000984	0.003067	62.51128	**Hpcal4**	5.216129	4.25E-05	0.000165	37.17161
**Ms4a4d**	5.964656	8.81E-08	5.35E-07	62.45115	**Cd86**	5.210641	2.14E-20	3.05E-18	37.03046
**Pilrb1**	5.956161	4.34E-15	1.19E-13	62.08451	**Il12rb1**	5.209766	9.72E-11	1.03E-09	37.00802
**Hdc**	5.928589	2.26E-17	1.14E-15	60.90924	**Tmem200b**	5.208213	2.04E-07	1.16E-06	36.96821
**Hsh2d**	5.926037	1.73E-09	1.43E-08	60.80158	**Slc43a3**	5.205447	1.03E-22	5.20E-20	36.8974
**Cd38**	5.920672	4.51E-23	3.26E-20	60.57588	**Enpp4**	5.194016	1.62E-19	1.77E-17	36.6062
**Timp1**	5.878346	2.71E-17	1.34E-15	58.82451	**Nags**	5.189073	1.79E-05	7.35E-05	36.48098
**Ifi211**	5.841317	1.13E-22	5.46E-20	57.33394	**Krt16**	5.182884	7.77E-06	3.39E-05	36.32482
**Marcksl1**	5.828724	1.23E-20	2.02E-18	56.83564	**Csprs**	5.176638	1.01E-11	1.32E-10	36.16791
**Adora2a**	5.811782	1.90E-22	8.10E-20	56.17209	**Bst2**	5.168324	2.72E-20	3.67E-18	35.96007
**Ifi214**	5.788302	1.26E-06	6.26E-06	55.26528	**Dll1**	5.147888	2.45E-06	1.16E-05	35.45428
**Socs1**	5.779803	7.98E-19	6.44E-17	54.94067	**Ddx60**	5.127376	5.12E-20	6.57E-18	34.95376
**Igtp**	5.776101	2.47E-20	3.40E-18	54.79989	**Irf1**	5.124116	1.44E-23	1.74E-20	34.87488
**Slc2a6**	5.764966	3.67E-23	2.75E-20	54.37856	**AC147806.1**	5.117218	2.83E-05	0.000113	34.70853
**Dnmt3c**	5.751821	0.000256	0.000876	53.88533	**Cd300e**	5.114967	5.59E-05	0.000212	34.6544
**Zmynd15**	5.75108	3.94E-21	7.63E-19	53.85768	**Tnfaip8l3**	5.090515	3.29E-07	1.81E-06	34.07201
**Ifnb1**	5.748372	0.000484	0.001582	53.75666	**Vcan**	5.08908	6.34E-16	2.20E-14	34.03813
**Phf11d**	5.745317	2.13E-21	4.92E-19	53.64295	**Stfa3**	5.088203	3.28E-06	1.53E-05	34.01744
**Cp**	5.738447	8.05E-16	2.70E-14	53.38812	**Ngf**	5.071262	0.00067	0.002143	33.62033
**Csf1**	5.725313	4.75E-24	8.42E-21	52.90428	**Ehd1**	5.062577	4.98E-23	3.40E-20	33.41854
**Hamp**	5.719301	8.94E-07	4.54E-06	52.6843	**Ier3**	5.059249	1.67E-16	6.68E-15	33.34155
**Slc1a2**	5.705352	1.15E-13	2.28E-12	52.17734	**Rtn2**	5.05312	3.17E-07	1.75E-06	33.20021
**Htr7**	5.701923	0.002556	0.007451	52.05348	**Bmp10**	5.03655	0.002316	0.006796	32.82106
**Sprr2d**	5.687071	0.000322	0.001085	51.52038	**Hspa1b**	5.024091	2.39E-14	5.53E-13	32.53884
**Pax4**	5.681776	0.002405	0.007044	51.33163	**Tnfrsf9**	5.020615	3.02E-08	2.00E-07	32.46053
**Serpinb2**	5.659746	4.79E-18	2.93E-16	50.55374	**Irgm2**	5.01367	3.12E-23	2.51E-20	32.30466
**Phf11b**	5.625006	8.65E-21	1.50E-18	49.35094	**Adm**	5.012232	3.39E-07	1.86E-06	32.27247
**Mefv**	5.619963	6.22E-22	1.82E-19	49.17875	**Oas1d**	4.985886	0.002893	0.00836	31.68847
**Plaat3**	5.618669761	5.85E-19	4.86E-17	49.13468022	**Susd2**	4.92894081	1.27E-08	8.91E-08	30.46204327

Gene	log2 (FC)	P-value	FDR	FC	Gene	log2 (FC)	P-value	FDR	FC
**Flrt3**	4.926825	8.62E-15	2.20E-13	30.41741	**Acp5**	4.442567	8.04E-19	6.46E-17	21.74432
**Casp4**	4.921014	2.93E-22	1.10E-19	30.29512	**Gm5431**	4.437675	1.19E-21	3.05E-19	21.67072
**Oas1a**	4.915572	2.65E-21	5.73E-19	30.18108	**Nt5c3**	4.436083	1.07E-21	2.79E-19	21.64682
**Ildr1**	4.914618	0.002122	0.00627	30.16112	**Ralgds**	4.431521	6.65E-21	1.18E-18	21.57848
**Mroh8**	4.906959	5.90E-05	0.000223	30.00142	**Pmepa1**	4.429184	1.34E-11	1.70E-10	21.54355
**Tnfsf15**	4.897792	1.73E-16	6.88E-15	29.81139	**Krt23**	4.42475	0.000482	0.001576	21.47744
**Ifit1bl2**	4.894427	1.20E-09	1.03E-08	29.74194	**Nlrp3**	4.407843	1.52E-19	1.69E-17	21.22721
**Il1rn**	4.888813	2.58E-21	5.73E-19	29.62642	**Ms4a6d**	4.399427	3.20E-21	6.37E-19	21.10374
**Tnfsf9**	4.867666	2.48E-20	3.40E-18	29.19534	**Slc6a4**	4.389618	4.41E-08	2.82E-07	20.96075
**Sh2d6**	4.857247	3.31E-09	2.60E-08	28.98525	**P3h2**	4.387998	9.32E-06	4.02E-05	20.93722
**Bcl2a1a**	4.853819	2.28E-19	2.32E-17	28.91646	**Pglyrp3**	4.382341	0.003014	0.008679	20.85528
**Nfkbie**	4.850644	6.78E-21	1.20E-18	28.8529	**Slamf9**	4.381453	1.15E-15	3.75E-14	20.84246
**Mycl**	4.844858	1.47E-12	2.32E-11	28.73741	**Acsl1**	4.371656	1.94E-23	1.95E-20	20.7014
**Sema7a**	4.843425	1.41E-06	6.96E-06	28.70887	**Jaml**	4.371482	3.47E-07	1.90E-06	20.69889
**Ube2l6**	4.83682	7.25E-23	4.25E-20	28.57775	**Ppp1r26**	4.370098	1.71E-06	8.30E-06	20.67904
**Gzma**	4.836158	7.52E-05	0.00028	28.56463	**Ddx58**	4.36175	9.73E-22	2.57E-19	20.55974
**Ms4a6c**	4.833435	1.19E-22	5.59E-20	28.51078	**Col5a3**	4.346911	3.34E-08	2.19E-07	20.34936
**Nfkbiz**	4.83108	1.27E-20	2.06E-18	28.46426	**Ccl3**	4.328243	2.98E-18	1.96E-16	20.08773
**Trim30b**	4.821151	4.36E-11	5.01E-10	28.26903	**Pgf**	4.327279	4.07E-06	1.86E-05	20.07431
**Upb1**	4.808564	0.002912	0.008406	28.02348	**Uba7**	4.302395	1.22E-20	2.02E-18	19.73103
**Kcng3**	4.798931	0.000241	0.00083	27.83699	**Sdk1**	4.301229	0.000143	0.000509	19.7151
**Trim72**	4.788021	5.88E-07	3.10E-06	27.62728	**Ntng2**	4.29275	5.49E-17	2.54E-15	19.59956
**Cyp26c1**	4.783693	0.000233	0.000805	27.54451	**Nfkbia**	4.288429	9.44E-23	4.87E-20	19.54095
**Il17a**	4.779417	0.003244	0.009296	27.463	**Vmn2r11**	4.286281	6.49E-11	7.14E-10	19.51188
**Prom1**	4.765254	5.31E-05	0.000202	27.19471	**Ly6e**	4.275161	4.11E-21	7.89E-19	19.36207
**Siglece**	4.763651	4.42E-08	2.83E-07	27.16452	**Tpbg**	4.274737	1.66E-11	2.06E-10	19.35638
**Pde11a**	4.735867	2.32E-06	1.10E-05	26.64636	**Lgals9**	4.273509	1.08E-20	1.84E-18	19.33991
**Ccdc116**	4.71122	0.001544	0.004669	26.19501	**Lif**	4.270572	2.59E-07	1.45E-06	19.30058
**Gvin1**	4.702752	7.02E-23	4.25E-20	26.04171	**Il4i1**	4.259929	8.23E-13	1.38E-11	19.15872
**1110032F04Rik**	4.69647	1.48E-06	7.27E-06	25.92856	**Ugt3a1**	4.254757	0.000112	0.000407	19.09015
**Herc6**	4.695086	2.52E-23	2.19E-20	25.90369	**Apol6**	4.251477	0.000389	0.001289	19.0468
**Gm7609**	4.687282	2.13E-09	1.73E-08	25.76395	**Nrg1**	4.248551	1.95E-07	1.11E-06	19.00821
**Fcgr1**	4.683566	2.01E-19	2.10E-17	25.69768	**Kazn**	4.226963	4.17E-10	3.93E-09	18.7259
**Dbx2**	4.668752	0.001665	0.005008	25.43516	**Parp14**	4.226752	8.28E-23	4.38E-20	18.72316
**H2-Oa**	4.664403	1.53E-07	8.90E-07	25.3586	**H2-Q4**	4.223932	6.78E-22	1.94E-19	18.6866
**Cxcl16**	4.66234	8.54E-22	2.35E-19	25.32235	**Nod2**	4.221651	1.57E-16	6.35E-15	18.65708
**Tap1**	4.662139	2.95E-22	1.10E-19	25.31883	**Wnk2**	4.218831	1.56E-12	2.44E-11	18.62064
**Has1**	4.646571	0.003315	0.009485	25.04708	**Il18bp**	4.202815	8.64E-13	1.44E-11	18.41507
**Pcsk1**	4.64394	0.002023	0.005996	25.00145	**Stx1b**	4.200408	0.000371	0.001235	18.38437
**Ikzf4**	4.637772	1.47E-05	6.15E-05	24.89478	**Ifi27l2a**	4.191924	1.84E-14	4.37E-13	18.27658
**Dennd3**	4.636299	1.36E-16	5.57E-15	24.86939	**Csf3**	4.186335	7.56E-09	5.51E-08	18.20591
**Hspa1a**	4.63469	8.46E-20	1.01E-17	24.84167	**Hbegf**	4.173504	1.43E-12	2.26E-11	18.04471
**Calcr**	4.623328	0.003241	0.009289	24.64679	**Stat2**	4.166717	1.16E-20	1.95E-18	17.96002
**Pilrb2**	4.59494	1.80E-14	4.30E-13	24.16655	**Gbp8**	4.163929	1.65E-07	9.55E-07	17.92535
**AC147806.2**	4.590423	4.70E-15	1.28E-13	24.09101	**Ifih1**	4.15528	2.73E-21	5.73E-19	17.81821
**Sh3bp5**	4.58582	2.71E-22	1.06E-19	24.01427	**1700019D03Rik**	4.145369	3.77E-07	2.06E-06	17.69622
**Xcl1**	4.583528	2.46E-06	1.17E-05	23.97614	**Crabp2**	4.141899	0.000156	0.000552	17.6537
**Gm4070**	4.570508	5.70E-21	1.05E-18	23.76075	**Trp53i11**	4.139924	3.93E-11	4.55E-10	17.62956
**Ifi204**	4.54248	2.50E-23	2.19E-20	23.30359	**Ifitm3**	4.138278	1.39E-20	2.17E-18	17.60945
**Gm12185**	4.536853	1.57E-15	4.91E-14	23.21287	**Klrg2**	4.137176	1.49E-05	6.19E-05	17.596
**Mndal**	4.533856	1.69E-22	7.48E-20	23.1647	**Hap1**	4.137115	1.15E-05	4.90E-05	17.59526
**Nudt17**	4.530359	5.46E-05	0.000208	23.10862	**Bcl2a1d**	4.136293	1.08E-17	5.87E-16	17.58524
**Gjb2**	4.522067	7.14E-05	0.000267	22.97618	**F830016B08Rik**	4.129492	1.54E-08	1.07E-07	17.50254
**Gm4841**	4.520479	4.42E-11	5.08E-10	22.9509	**C1rl**	4.124344	1.44E-12	2.27E-11	17.4402
**Il15**	4.500102	1.72E-20	2.56E-18	22.62901	**A1cf**	4.115462	0.000225	0.000779	17.33316
**Ifi35**	4.49833	1.71E-20	2.56E-18	22.60123	**Nmi**	4.113921	2.67E-21	5.73E-19	17.31465
**C1ra**	4.489856	3.65E-18	2.33E-16	22.46887	**Dhx58**	4.111235	5.94E-22	1.79E-19	17.28244
**Nlrc5**	4.470041	3.45E-22	1.21E-19	22.16239	**Smpdl3b**	4.109513	1.93E-13	3.66E-12	17.26183
**Timd4**	4.458092	2.27E-07	1.28E-06	21.97958	**Elfn1**	4.103988	2.51E-05	0.000101	17.19584
**Nfkb2**	4.455903	7.45E-23	4.25E-20	21.94626	**Mmp13**	4.098059	8.01E-12	1.06E-10	17.12532
**Fam171b**	4.448979	2.06E-09	1.68E-08	21.84118	**Ly6a**	4.092089	8.46E-13	1.42E-11	17.0546
**Irgm1**	4.448686	2.37E-22	9.70E-20	21.83674	**Ereg**	4.091333	4.52E-05	0.000174	17.04566
**Myrfl**	4.447945	0.002804	0.008121	21.82553	**Stat1**	4.074393	7.85E-23	4.33E-20	16.84669
**Slfn8**	4.445646	1.39E-22	6.40E-20	21.79079	**Casp7**	4.071544	1.89E-21	4.46E-19	16.81346
**Krt4**	4.44455	0.00246	0.007193	21.77424	**Gm19684**	4.070791	5.66E-10	5.16E-09	16.80467

Gene	log2 (FC)	P-value	FDR	FC	Gene	log2 (FC)	P-value	FDR	FC
**Fbxo39**	4.067046466	0.00072850	0.002311	16.761117	**Chic1**	3.720074282	4.75E-05	0.000182408	13.178134
**Helz2**	4.062026437	4.39E-21	8.27E-19	16.702896	**Ciita**	3.71779574	7.01E-17	3.13E-15	13.157338
**Car6**	4.054728421	3.09E-16	1.16E-14	16.618617	**Adgb**	3.714091108	4.19E-06	1.92E-05	13.123595
**Xkr8**	4.052076469	2.72E-16	1.03E-14	16.588096	**Fas**	3.714085849	1.95E-19	2.05E-17	13.123547
**Tnfrsf8**	4.027314262	1.65E-07	9.56E-07	16.305810	**Penk**	3.708549369	7.85E-09	5.70E-08	13.073281
**Filip1l**	4.023075275	1.51E-20	2.33E-18	16.257970	**Ccl9**	3.704428729	1.53E-18	1.11E-16	13.035994
**Trim30a**	4.016400088	1.86E-22	8.07E-20	16.182920	**A530032D15Rik**	3.699744335	1.33E-13	2.60E-12	12.993735
**Flt4**	4.014301858	1.02E-08	7.27E-08	16.159401	**Cd274**	3.692662359	3.08E-21	6.18E-19	12.930107
**Orm1**	4.013340287	7.90E-13	1.33E-11	16.148634	**Osbpl3**	3.665796105	1.35E-18	9.99E-17	12.691547
**Src**	4.009850397	1.92E-19	2.04E-17	16.109618	**Pdcd1lg2**	3.664511279	1.91E-09	1.57E-08	12.680249
**Cdc42ep2**	4.001748232	1.89E-17	9.76E-16	16.019400	**Glrp1**	3.663616728	4.32E-16	1.56E-14	12.672389
**Gm45799**	3.998775269	8.61E-05	0.000318	15.98642306	**Cdh17**	3.659770086	0.002567779	0.007481477	12.63864668
**Coch**	3.998048063	2.55E-06	1.20E-05	15.978366	**Sphk1**	3.65458525	2.00E-11	2.45E-10	12.593306
**F10**	3.976663739	8.64E-22	2.35E-19	15.743274	**Maff**	3.653046507	4.13E-16	1.51E-14	12.579882
**Met**	3.964576828	2.73E-19	2.66E-17	15.6119282	**Car3**	3.652125812	0.002248073	0.00661397	12.57185656
**Plpp1**	3.959123317	1.02E-13	2.05E-12	15.553025	**Wars**	3.645160918	3.62E-22	1.25E-19	12.511309
**Pappa2**	3.947216315	0.000430285	0.001415547	15.42518958	**Plekha4**	3.643434513	5.52E-07	2.93E-06	12.49634698
**Tmprss2**	3.943809261	0.00222304	0.00654	15.388804	**Gm8113**	3.642639636	4.52E-19	4.00E-17	12.489463
**Pamr1**	3.935303472	0.00013143	0.000472	15.298342	**Slc28a2**	3.633328431	3.63E-21	7.09E-19	12.409115
**Rnf213**	3.934505521	5.32E-20	6.75E-18	15.289883	**Lpar1**	3.631681386	3.24E-11	3.81E-10	12.394957
**Psme2**	3.928857103	2.21E-19	2.27E-17	15.230137	**Krt13**	3.629946692	0.0012617	0.0038629	12.38006
**Tet1**	3.91923581	2.04E-06	9.81E-06	15.128906	**Lgals3bp**	3.629212462	3.81E-19	3.48E-17	12.373763
**Trim30d**	3.915761392	4.37E-22	1.48E-19	15.092515	**Slfn9**	3.62536816	3.83E-18	2.41E-16	12.340835
**Tapbpl**	3.913267816	2.74E-22	1.06E-19	15.066452	**Sass6**	3.624858437	5.08E-13	8.88E-12	12.336476
**Dlgap3**	3.902905508	0.00030019	0.001016	14.958623	**Psmb9**	3.624462657	9.92E-20	1.18E-17	12.333092
**Fam167a**	3.896294694	5.74E-05	0.000217	14.890235	**Samd9l**	3.616765772	9.06E-22	2.43E-19	12.267469
**Cnn3**	3.883901761	3.09E-15	8.79E-14	14.762874	**Gipc3**	3.606841129	2.11E-05	8.59E-05	12.183368
**Gzmb**	3.882073108	9.37E-05	0.000343	14.744174	**Rnd3**	3.602821956	1.17E-18	8.95E-17	12.149474
**Neb**	3.881219278	5.18E-07	2.76E-06	14.735450	**Zc3h12c**	3.598885297	1.85E-19	1.99E-17	12.116367
**Bdkrb1**	3.867750753	3.49E-05	0.000137333	14.598525	**Tmem178**	3.596808839	9.88E-17	4.19E-15	12.098940
**Hao1**	3.864944243	0.0030389	0.008741	14.570154	**Zdhhc15**	3.593979327	1.74E-10	1.76E-09	12.075234
**Ndufa4l2**	3.853241346	0.00133148	0.004061	14.452441	**Eif2ak2**	3.587943884	2.95E-19	2.81E-17	12.024824
**Mov10**	3.849726934	3.83E-19	3.48E-17	14.417278	**Gm20075**	3.586875725	0.0001205	0.0004359	12.015924
**Gfi1**	3.845013766	7.44E-05	0.000277	14.370255	**Slc25a37**	3.578940244	1.68E-12	2.61E-11	11.950012
**Slfn2**	3.842384564	1.16E-20	1.95E-18	14.344090	**Samd11**	3.556120779	1.27E-05	5.35E-05	11.762483
**Mki67**	3.841945871	6.10E-14	1.30E-12	14.339729	**Gm49342**	3.548535485	2.92E-17	1.43E-15	11.700801
**Rnd1**	3.837491688	5.69E-14	1.22E-12	14.295524	**Jak2**	3.545451419	6.15E-22	1.82E-19	11.675815
**Gm36079**	3.833463557	0.00072094	0.002290	14.255666	**Agrn**	3.539707845	6.07E-18	3.57E-16	11.629424
**Ccl4**	3.832175118	2.37E-16	9.10E-15	14.242940	**Adgrf1**	3.523800988	0.0002604	0.0008901	11.501905
**Psmb10**	3.814163234	3.68E-20	4.90E-18	14.066224	**Col9a3**	3.520239637	1.57E-06	7.66E-06	11.473547
**Ccne1**	3.810639729	1.16E-17	6.24E-16	14.031912	**Nr4a3**	3.510915643	2.40E-07	1.35E-06	11.399634
**Ms4a4a**	3.810273954	7.84E-12	1.04E-10	14.028355	**Ddit3**	3.509784494	1.66E-20	2.52E-18	11.390699
**1600014C10Rik**	3.809261189	3.91E-17	1.85E-15	14.018510	**Tgm2**	3.508643638	5.52E-22	1.69E-19	11.381695
**Gpr85**	3.803627253	5.70E-12	7.84E-11	13.963873	**Tspan33**	3.500314742	0.0005310	0.0017230	11.316176
**Mybpc2**	3.802373652	0.00253568	0.007397	13.951744	**Pde4b**	3.494170804	1.19E-17	6.40E-16	11.268087
**Ifitm1**	3.793925991	2.26E-06	1.08E-05	13.870289	**Tnfsf14**	3.48592842	0.0004397	0.0014439	11.203894
**Daxx**	3.791032452	2.83E-21	5.85E-19	13.842498	**St3gal5**	3.470870149	7.72E-20	9.36E-18	11.087561
**Slc39a2**	3.789129062	3.96E-08	2.56E-07	13.824247	**Tlr3**	3.465465073	2.00E-14	4.72E-13	11.046099
**Cybb**	3.778723676	1.35E-20	2.13E-18	13.724899	**Asb11**	3.465067324	5.70E-05	0.0002161	11.043054
**Sod2**	3.774772681	7.69E-21	1.34E-18	13.687363	**Frzb**	3.464102744	0.0008278	0.0026091	11.035673
**Tjp1**	3.768649114	5.94E-14	1.27E-12	13.629390	**Mmp14**	3.463891418	4.41E-19	3.94E-17	11.034056
**Cflar**	3.767838419	2.73E-21	5.73E-19	13.621733	**Rnf144a**	3.463318129	2.09E-08	1.42E-07	11.029673
**AC167036.1**	3.763482977	8.81E-06	3.81E-05	13.580672	**Lrrc8c**	3.463050365	1.88E-19	2.01E-17	11.027626
**Aoah**	3.761563991	3.11E-19	2.91E-17	13.562619	**Mmp25**	3.457173485	0.0002329	0.0008038	10.982796
**Hpx**	3.752630026	0.00121325	0.003725	13.47889	**Il17rd**	3.454050215	1.31E-07	7.74E-07	10.959045
**Fst**	3.74820949	0.00333933	0.009548	13.437655	**Sp110**	3.431430907	1.60E-18	1.16E-16	10.788563
**Itgal**	3.747132222	2.63E-19	2.59E-17	13.4276	**Ak4**	3.429877305	1.89E-15	5.71E-14	10.776952
**Dnmt3l**	3.741277555	0.00019763	0.000690	13.37324	**Serpinb9**	3.426733202	6.36E-18	3.68E-16	10.753491
**Phf11c**	3.73986376	5.10E-13	8.91E-12	13.360145	**Krt5**	3.426687458	0.0011391	0.0035144	10.753150
**Akap12**	3.737472624	1.54E-08	1.07E-07	13.338020	**Psmb8**	3.425998443	1.96E-18	1.36E-16	10.748015
**Gch1**	3.730389851	2.46E-19	2.47E-17	13.272698	**Ass1**	3.425739063	1.49E-17	7.84E-16	10.746083
**Depp1**	3.729432439	3.02E-07	1.67E-06	13.263893	**Gng4**	3.425728958	1.37E-08	9.61E-08	10.746008
**Serpine2**	3.726646241	2.03E-08	1.38E-07	13.238302	**Slfn3**	3.420838042	4.81E-16	1.73E-14	10.709639
**Ablim2**	3.725182118	6.42E-13	1.10E-11	13.224874	**Rcn1**	3.404722998	1.12E-16	4.70E-15	10.590677
**Kit**	3.721790687	3.83E-07	2.09E-06	13.19382	**Znfx1**	3.400200595	7.50E-20	9.14E-18	10.557531

Gene	log2 (FC)	P-value	FDR	FC	Gene	log2 (FC)	P-value	FDR	FC
**Car13**	3.399518686	4.39E-21	8.27E-19	10.55254214	**Lag3**	3.076630784	0.000264	0.000903301	8.436419205
**Pnp2**	3.399517766	4.65E-12	6.56E-11	10.55253541	**Svep1**	3.074605984	3.18E-07	1.75E-06	8.424587128
**Batf**	3.398822429	9.86E-19	7.66E-17	10.54745062	**Scd4**	3.06194212	4.53E-07	2.43E-06	8.350960375
**Dusp4**	3.397182616	2.56E-19	2.55E-17	10.53546887	**Osm**	3.048734275	3.63E-16	1.35E-14	8.274856393
**Dtx3l**	3.392082185	1.70E-20	2.56E-18	10.49828807	**Epop**	3.047098732	3.68E-06	1.70E-05	8.265480737
**Ppp1r15a**	3.388017725	5.23E-20	6.67E-18	10.46875319	**Rgs1**	3.032783937	2.56E-17	1.28E-15	8.183874023
**Hrc**	3.386639057	2.91E-07	1.61E-06	10.45875382	**Slc31a2**	3.03073984	6.76E-19	5.55E-17	8.172286829
**Procr**	3.378526358	1.20E-17	6.45E-16	10.40010622	**Tap2**	3.030266562	1.03E-17	5.66E-16	8.169606338
**Csrnp1**	3.377675727	3.91E-18	2.45E-16	10.39397599	**Naa25**	3.029652839	1.37E-19	1.54E-17	8.166131723
**Pnp**	3.371726631	2.30E-18	1.57E-16	10.35120365	**Sp100**	3.02688459	5.76E-20	7.21E-18	8.150477541
**Camk2n1**	3.358935671	3.61E-09	2.80E-08	10.25983533	**Htr1b**	3.020151114	0.003483	0.009928086	8.112525534
**Trim21**	3.35735223	9.89E-20	1.18E-17	10.24858075	**Milr1**	3.019193991	1.74E-18	1.24E-16	8.107145251
**S1pr3**	3.338392212	2.13E-07	1.21E-06	10.11477422	**Adamts14**	3.018508178	1.95E-09	1.60E-08	8.103292277
**Pde10a**	3.337971501	0.000101513	0.000370807	10.11182504	**Psme1**	3.015091673	1.28E-19	1.45E-17	8.084125243
**Jsrp1**	3.336619878	0.00114	0.003518	10.10235597	**Stxbp6**	3.010480724	8.52E-08	5.19E-07	8.058329087
**Il2ra**	3.331941445	1.75E-07	1.01E-06	10.06964868	**Col23a1**	3.006639383	3.47E-09	2.71E-08	8.036901406
**Slamf6**	3.329930133	3.40E-09	2.66E-08	10.05562001	**AC133103.1**	3.006561546	0.001646512	0.004959084	8.036467802
**Bcl2l14**	3.319596695	0.001876767	0.005599055	9.983853016	**Rab11fip1**	3.000852498	4.83E-19	4.19E-17	8.004728652
**Rab32**	3.315529449	2.75E-21	5.73E-19	9.955746179	**Nfkbib**	2.990455438	4.82E-19	4.19E-17	7.947248397
**Epsti1**	3.31225465	1.47E-18	1.07E-16	9.93317308	**Dusp28**	2.989287931	5.27E-14	1.14E-12	7.940819658
**Prdx5**	3.304613799	8.64E-18	4.84E-16	9.880703738	**Tent4a**	2.989011161	5.84E-18	3.46E-16	7.939296416
**Clcf1**	3.292925082	8.38E-06	3.64E-05	9.800973686	**Tent5c**	2.978960603	2.20E-19	2.27E-17	7.884179393
**Il21r**	3.291286628	6.61E-11	7.26E-10	9.789849138	**Intu**	2.97857321	7.84E-16	2.65E-14	7.882062613
**Parp12**	3.271341824	1.24E-20	2.02E-18	9.655438793	**Ogfr**	2.959222876	1.64E-20	2.50E-18	7.777049258
**Slc6a9**	3.259934905	9.14E-10	8.03E-09	9.579397403	**Shfl**	2.95680014	7.26E-11	7.90E-10	7.764000117
**Foxq1**	3.250487344	1.00E-05	4.30E-05	9.516871186	**Oas1c**	2.954821789	4.24E-13	7.54E-12	7.753360729
**Ccr7**	3.250095206	2.63E-09	2.10E-08	9.514284764	**Spint1**	2.954742816	1.77E-07	1.02E-06	7.752936324
**Exoc3l4**	3.247333235	4.41E-13	7.82E-12	9.496087542	**Parp10**	2.953608381	1.42E-16	5.80E-15	7.746842351
**Ell2**	3.245902625	6.79E-18	3.85E-16	9.486675667	**Soat2**	2.950514829	6.88E-16	2.36E-14	7.730248697
**Gm29094**	3.24319346	4.06E-13	7.26E-12	9.468877827	**Cth**	2.937426225	7.76E-06	3.38E-05	7.660434506
**Flt3**	3.231415304	0.00198	0.005905	9.391888649	**Acy1**	2.935323013	1.92E-11	2.35E-10	7.649274987
**Prrg4**	3.229967346	4.63E-07	2.49E-06	9.382467227	**Tor3a**	2.933637929	6.05E-20	7.53E-18	7.640345765
**Sult6b1**	3.22539687	3.15E-06	1.47E-05	9.352790488	**Bcl2a1b**	2.917062068	5.38E-17	2.49E-15	7.553064297
**Hp**	3.194765763	1.38E-12	2.19E-11	9.156306543	**Tmeff1**	2.902319475	0.000378	0.001258278	7.47627416
**Ermap**	3.181136634	0.00118	0.003650	9.07021428	**Olfm1**	2.898053164	5.75E-11	6.42E-10	7.454198119
**Nupr1**	3.18021593	1.49E-17	7.83E-16	9.064427664	**Boc**	2.894161425	0.000403	0.001332549	7.43411716
**Rhof**	3.179658537	1.69E-06	8.20E-06	9.060926253	**Mitd1**	2.890809721	6.09E-18	3.57E-16	7.416866087
**Gsap**	3.178698342	1.32E-16	5.41E-15	9.054897701	**Rfx5**	2.88882807	2.10E-10	2.08E-09	7.406685452
**Zfp945**	3.167546262	8.09E-17	3.52E-15	8.985172879	**Setdb2**	2.886471724	8.16E-18	4.59E-16	7.394598028
**C130026I21Rik**	3.164103927	2.25E-14	5.23E-13	8.963759409	**Spata13**	2.885641184	6.14E-09	4.57E-08	7.390342284
**Saa3**	3.156970214	4.87E-10	4.51E-09	8.919545592	**Micall2**	2.884533748	6.94E-15	1.81E-13	7.384671515
**H2-Ab1**	3.154803446	1.45E-18	1.06E-16	8.906159482	**Trim12a**	2.876892506	3.77E-19	3.48E-17	7.345661961
**Zup1**	3.153014132	4.01E-18	2.50E-16	8.895120392	**Zswim5**	2.874934099	9.15E-05	0.000336218	7.335697248
**Olfr898**	3.142275202	0.00072	0.002296	8.829153966	**Mpp2**	2.870695083	0.000559	0.001808448	7.314174682
**Apobec3**	3.142252554	1.01E-19	1.19E-17	8.82901537	**Adgre4**	2.869857362	0.000887	0.00278456	7.309928838
**Camp**	3.138909701	0.00052	0.001712	8.808581434	**Glipr2**	2.867077108	1.03E-15	3.37E-14	7.295855258
**Adora2b**	3.137727089	3.99E-12	5.71E-11	8.801363786	**Parp9**	2.863441883	6.20E-19	5.13E-17	7.277494698
**Rtn4r**	3.136120747	3.92E-08	2.54E-07	8.791569522	**Ets2**	2.853993566	4.87E-19	4.20E-17	7.229989569
**Ly6c2**	3.129222523	6.07E-09	4.53E-08	8.749633109	**Tpst1**	2.851059134	1.61E-16	6.46E-15	7.215298765
**Tapbp**	3.125992164	4.94E-20	6.38E-18	8.7300636	**Tbx21**	2.84934109	0.000955	0.002984675	7.206711489
**Ctla2b**	3.123535939	4.49E-05	0.000173	8.715213091	**Plau**	2.844183934	2.63E-19	2.59E-17	7.180995875
**Tagap**	3.122744064	3.25E-15	9.24E-14	8.710430742	**Ece1**	2.843721918	3.27E-16	1.22E-14	7.178696567
**Ccrl2**	3.113081313	2.67E-18	1.79E-16	8.652285754	**Tor1aip2**	2.841347887	1.10E-19	1.26E-17	7.166893353
**Srgn**	3.11003761	1.70E-18	1.22E-16	8.634050971	**Creb5**	2.841308335	3.59E-18	2.30E-16	7.166696875
**Nod1**	3.105600646	4.03E-16	1.48E-14	8.607538008	**Gpr31b**	2.836753538	0.000287	0.000976897	7.144106254
**Ripk2**	3.104471089	1.07E-18	8.19E-17	8.600801381	**Tspo**	2.833973173	7.09E-16	2.43E-14	7.130351377
**Parp11**	3.103112313	2.66E-17	1.32E-15	8.592704685	**Trim34a**	2.827754876	1.23E-18	9.29E-17	7.099684311
**Themis**	3.094566378	0.00259	0.007546	8.541955477	**Cdkn1a**	2.812440825	2.91E-19	2.80E-17	7.024720501
**Pou3f1**	3.093159487	7.16E-15	1.86E-13	8.533629573	**Alox12**	2.811744659	0.002849	0.008238426	7.021331575
**Ggct**	3.092449957	2.66E-14	6.08E-13	8.529433689	**Tnfaip3**	2.800438555	4.05E-20	5.33E-18	6.966521891
**Atp13a4**	3.092350074	0.00245	0.007191	8.528843183	**Gimap9**	2.799793011	1.47E-09	1.24E-08	6.96340537
**Tmem67**	3.087840693	3.79E-18	2.40E-16	8.502226498	**Igsf8**	2.79961813	1.32E-18	9.85E-17	6.962561326
**Rnf180**	3.080813723	1.59E-11	1.98E-10	8.460915185	**Phykpl**	2.796040629	5.45E-11	6.11E-10	6.945317412
**Sod3**	3.079160669	3.01E-05	0.000119	8.451226142	**Gm49355**	2.793875368	0.000266	0.000909306	6.93490139
**Rph3a**	3.078459461	4.72E-06	2.14E-05	8.447119499	**Sp140**	2.792704843	2.48E-18	1.68E-16	6.929277063

Gene	log2 (FC)	P-value	FDR	FC	Gene	log2 (FC)	P-value	FDR	FC
**Gpr18**	2.78867	0.000144	0.000513	6.909923	**Serpina3n**	2.531282	8.38E-07	4.28E-06	5.780852
**Batf3**	2.787876	6.64E-11	7.29E-10	6.906121	**Rmi2**	2.529634	0.000261	0.00089	5.77425
**Rnf114**	2.784547	4.99E-19	4.27E-17	6.890206	**Slc7a3**	2.529055	0.001976	0.005867	5.771934
**H2-T22**	2.783437	1.95E-16	7.62E-15	6.884906	**Idnk**	2.526847	2.16E-17	1.10E-15	5.763106
**Fhod3**	2.778741	0.000135	0.000484	6.862534	**Traf2**	2.523074	4.66E-18	2.87E-16	5.748056
**Lmo4**	2.772137	1.24E-18	9.36E-17	6.83119	**Mtmr7**	2.517201	2.58E-07	1.45E-06	5.724705
**Lilrb4a**	2.766279	0.001106	0.00342	6.803508	**Antxr1**	2.51119	0.000159	0.000562	5.700901
**Pglyrp2**	2.760143	4.15E-05	0.000161	6.774633	**Foxp4**	2.50858	1.59E-14	3.85E-13	5.690597
**Trib3**	2.754096	1.02E-18	7.89E-17	6.746298	**Gm21748**	2.507925	7.81E-07	4.02E-06	5.688015
**Dab2**	2.75358	6.70E-12	9.02E-11	6.743885	**Il13ra1**	2.505686	1.62E-19	1.77E-17	5.679195
**Inava**	2.746486	0.000194	0.00068	6.710807	**Nckap1**	2.497994	1.59E-12	2.48E-11	5.648995
**Hck**	2.742574	1.03E-19	1.20E-17	6.692634	**Aldh1l2**	2.49026	9.18E-07	4.65E-06	5.618794
**Lck**	2.728942	7.08E-10	6.33E-09	6.629691	**Slc15a3**	2.489407	2.08E-19	2.17E-17	5.61547
**Abtb2**	2.715335	2.08E-12	3.14E-11	6.567457	**Pdzrn3**	2.486564	0.0002	0.000698	5.604416
**Ccl24**	2.713251	9.26E-05	0.00034	6.557978	**Tmem243**	2.48453	9.72E-14	1.96E-12	5.596518
**Smad6**	2.702464	1.65E-05	6.81E-05	6.509129	**Wdr20rt**	2.482496	0.001312	0.004004	5.588637
**Denr**	2.700272	9.91E-18	5.47E-16	6.499244	**Aftph**	2.479273	1.57E-19	1.74E-17	5.576163
**Phyhd1**	2.699705	7.31E-15	1.90E-13	6.496691	**Gpr141**	2.477512	3.06E-17	1.49E-15	5.569362
**Rac3**	2.696119	1.72E-05	7.07E-05	6.480562	**Notch1**	2.474247	1.05E-14	2.64E-13	5.556773
**Stx2**	2.693418	4.12E-18	2.56E-16	6.468439	**Myd88**	2.469193	3.52E-18	2.27E-16	5.53734
**Hrh1**	2.687837	0.001176	0.003617	6.443467	**Cysltr2**	2.466257	3.57E-07	1.95E-06	5.526083
**Slc7a8**	2.685471	1.48E-16	5.99E-15	6.432909	**Nrp2**	2.464542	9.21E-19	7.21E-17	5.519518
**Lox**	2.67991	4.60E-12	6.51E-11	6.408159	**Dck**	2.463823	4.05E-16	1.48E-14	5.516766
**Hook2**	2.679531	1.94E-16	7.58E-15	6.406477	**Cyb5r1**	2.457315	1.91E-18	1.33E-16	5.491935
**Il18**	2.667587	8.79E-16	2.92E-14	6.353658	**Ifrd1**	2.455198	5.10E-17	2.37E-15	5.483884
**Ms4a14**	2.666705	3.58E-11	4.17E-10	6.349772	**Adamts7**	2.443978	1.65E-05	6.79E-05	5.4414
**Ms4a7**	2.666617	1.38E-16	5.64E-15	6.349385	**Txlnb**	2.442825	0.000701	0.002233	5.437054
**Gimap6**	2.66642	1.51E-10	1.54E-09	6.348517	**Gm21188**	2.439707	8.07E-09	5.85E-08	5.425317
**Malt1**	2.666406	3.94E-19	3.56E-17	6.348459	**Ccdc86**	2.432708	2.64E-17	1.31E-15	5.399059
**Ms4a4b**	2.666264	9.71E-07	4.90E-06	6.34783	**Schip1**	2.426202	7.52E-09	5.49E-08	5.374767
**Zcwpw1**	2.662605	6.52E-11	7.17E-10	6.331751	**Rbpj**	2.42429	1.03E-16	4.36E-15	5.367646
**Slpi**	2.656165	1.37E-09	1.16E-08	6.30355	**Lap3**	2.422071	2.45E-18	1.66E-16	5.359397
**Rnf135**	2.655173	2.21E-15	6.53E-14	6.299221	**Gtpbp2**	2.421255	2.03E-17	1.04E-15	5.356367
**C2**	2.653327	5.03E-10	4.64E-09	6.291164	**H2-K1**	2.420983	8.50E-18	4.77E-16	5.355358
**Cd83**	2.65267	1.11E-14	2.78E-13	6.288298	**Mapk11**	2.42044	8.32E-06	3.61E-05	5.353344
**Six5**	2.639744	0.00045	0.001476	6.232212	**Zc3h12a**	2.419061	3.30E-18	2.16E-16	5.348228
**Eng**	2.637213	1.07E-16	4.51E-15	6.221287	**Adar**	2.417731	1.03E-19	1.20E-17	5.343299
**Tnfrsf18**	2.633437	0.002648	0.007698	6.205026	**Lcp2**	2.410384	5.44E-18	3.26E-16	5.316159
**Irf9**	2.632663	5.46E-19	4.59E-17	6.201695	**Msr1**	2.40346	7.45E-19	6.05E-17	5.290706
**C3**	2.631005	1.33E-13	2.60E-12	6.194574	**Nxph1**	2.396506	0.001154	0.003554	5.265266
**Pml**	2.622507	7.31E-17	3.23E-15	6.158191	**Stat5a**	2.394383	3.04E-11	3.59E-10	5.257524
**Psmd10**	2.613908	6.18E-18	3.60E-16	6.121595	**Slc25a22**	2.389956	2.07E-13	3.90E-12	5.241413
**Spred1**	2.613895	2.78E-18	1.86E-16	6.121542	**Rab11fip4**	2.386929	4.79E-08	3.05E-07	5.230429
**Agtrap**	2.611912	6.10E-20	7.56E-18	6.113133	**Sppl2a**	2.386697	1.06E-19	1.22E-17	5.229588
**Usb1**	2.611204	7.85E-17	3.43E-15	6.110133	**Kdm6b**	2.383948	6.76E-13	1.15E-11	5.219633
**Skil**	2.610672	1.90E-18	1.33E-16	6.107882	**Tnfrsf1b**	2.37876	1.63E-18	1.17E-16	5.200894
**Nkg7**	2.609906	0.000763	0.002415	6.104641	**Olfr1033**	2.377145	2.86E-12	4.21E-11	5.195075
**Il2rb**	2.609231	1.62E-06	7.88E-06	6.101782	**Slfn5**	2.37415	4.80E-19	4.19E-17	5.184303
**Zdhhc2**	2.602279	0.000251	0.000862	6.07245	**Stx11**	2.373853	2.95E-17	1.44E-15	5.183237
**Ankmy1**	2.591459	0.001816	0.005434	6.027078	**Creb3l1**	2.373852	7.63E-10	6.77E-09	5.183233
**Tnfrsf11b**	2.588954	0.001037	0.00322	6.016625	**Aida**	2.367351	2.03E-16	7.91E-15	5.159928
**Hid1**	2.581447	3.69E-06	1.71E-05	5.985399	**Samhd1**	2.36283	8.82E-17	3.81E-15	5.143782
**Trim46**	2.581382	0.000203	0.000706	5.985129	**Fanca**	2.360993	1.72E-07	9.91E-07	5.137237
**Slamf8**	2.570498	1.81E-17	9.38E-16	5.940146	**Cebpb**	2.359987	5.09E-19	4.35E-17	5.133658
**Fam102b**	2.569612	2.25E-19	2.30E-17	5.936499	**Casp1**	2.359037	2.94E-19	2.81E-17	5.130279
**Gpr132**	2.5665	1.72E-15	5.31E-14	5.923707	**Ms4a6b**	2.356907	7.00E-08	4.31E-07	5.12271
**Stc2**	2.564422	1.11E-05	4.72E-05	5.91518	**Irf4**	2.352494	1.86E-08	1.27E-07	5.107063
**Gm6377**	2.561579	2.20E-12	3.31E-11	5.903534	**Abca1**	2.349817	1.04E-16	4.38E-15	5.097595
**Clcn7**	2.559215	2.05E-16	7.98E-15	5.893867	**Snn**	2.344561	5.43E-11	6.09E-10	5.079058
**Slc31a1**	2.557487	1.25E-19	1.42E-17	5.886815	**Msantd3**	2.340192	9.88E-08	5.95E-07	5.063701
**Dcbld2**	2.555898	3.07E-19	2.90E-17	5.880332	**Atp10a**	2.33768	8.04E-16	2.70E-14	5.05489
**H2-M3**	2.555343	3.29E-15	9.31E-14	5.878071	**Pstpip2**	2.337346	8.97E-12	1.18E-10	5.05372
**Adamts4**	2.543961	0.003221	0.009237	5.831878	**Birc3**	2.336931	1.19E-18	9.09E-17	5.052266
**Pcgf5**	2.53954	6.44E-18	3.70E-16	5.814037	**Pla2g4a**	2.336565	2.77E-17	1.37E-15	5.050984
**Ghitm**	2.535646	7.70E-19	6.23E-17	5.798364	**Arhgef3**	2.331985	1.77E-17	9.23E-16	5.034977

Gene	log2 (FC)	P-value	FDR	FC	Gene	log2 (FC)	P-value	FDR	FC
**Apol7c**	2.330958	5.44E-08	3.43E-07	5.031394	**Tnfrsf14**	2.184537	1.74E-13	3.33E-12	4.545809
**Eif6**	2.325225	9.06E-18	5.05E-16	5.01144	**Rbl1**	2.180847	6.63E-16	2.28E-14	4.534196
**Whamm**	2.324203	1.50E-14	3.67E-13	5.007889	**Rnaset2b**	2.166717	1.72E-15	5.31E-14	4.490006
**Plac8**	2.322575	6.55E-09	4.83E-08	5.002244	**Mylk2**	2.16213	3.56E-05	0.00014	4.475752
**Bid**	2.320574	5.72E-18	3.40E-16	4.995309	**Jag1**	2.158545	5.83E-13	1.01E-11	4.464644
**E2f5**	2.318266	8.52E-15	2.18E-13	4.987326	**Gde1**	2.154025	1.40E-16	5.69E-15	4.450676
**Dgat2**	2.318228	2.70E-05	0.000108	4.987191	**Zfp507**	2.15282	1.60E-08	1.11E-07	4.446963
**Crim1**	2.316199	6.88E-14	1.44E-12	4.980185	**Cd302**	2.152499	7.70E-16	2.61E-14	4.445973
**Cd74**	2.312505	2.27E-18	1.55E-16	4.967448	**Rhoh**	2.15005	2.03E-13	3.83E-12	4.438431
**Prss35**	2.312446	3.31E-06	1.54E-05	4.967244	**Myo16**	2.149356	0.001234	0.003785	4.436298
**Psma5**	2.309815	1.92E-16	7.53E-15	4.958194	**Selenow**	2.144596	1.19E-14	2.97E-13	4.421684
**H2-Aa**	2.305364	7.36E-16	2.51E-14	4.942922	**Rnf31**	2.143925	8.09E-16	2.72E-14	4.419628
**Ptgir**	2.30086	5.92E-16	2.07E-14	4.927516	**Relb**	2.140942	1.37E-15	4.34E-14	4.4105
**Spry1**	2.299792	7.88E-06	3.43E-05	4.923868	**Nfkb1**	2.140717	3.72E-18	2.37E-16	4.409813
**Ccn2**	2.298388	6.86E-05	0.000257	4.919079	**Lrp11**	2.140312	4.10E-14	9.04E-13	4.408575
**Timeless**	2.298306	3.79E-08	2.46E-07	4.9188	**Trmt10a**	2.132296	4.43E-10	4.14E-09	4.384146
**H2-DMb1**	2.29619	7.67E-15	1.98E-13	4.911589	**Tnip3**	2.130311	1.51E-12	2.37E-11	4.378119
**Pdpn**	2.292275	3.73E-18	2.37E-16	4.898281	**Myo19**	2.12797	0.000155	0.000549	4.371019
**Sh3tc1**	2.290423	8.92E-19	7.03E-17	4.891994	**Arrdc4**	2.126931	9.25E-18	5.13E-16	4.367875
**Riox2**	2.284458	4.69E-18	2.88E-16	4.87181	**Azi2**	2.126245	4.92E-18	2.99E-16	4.365798
**Sco1**	2.283785	1.83E-15	5.57E-14	4.869537	**B230307C23Rik**	2.122016	4.87E-12	6.84E-11	4.353018
**Hk2**	2.282025	3.35E-17	1.61E-15	4.863603	**Kpna3**	2.121162	5.38E-19	4.54E-17	4.350443
**Eef1e1**	2.280849	1.62E-14	3.92E-13	4.859637	**Olfr853**	2.119784	4.53E-05	0.000174	4.34629
**Abracl**	2.278871	6.41E-15	1.68E-13	4.85298	**Btg1**	2.116861	6.71E-18	3.83E-16	4.337492
**Junb**	2.278463	1.33E-18	9.93E-17	4.851608	**Rnf19b**	2.114319	6.07E-17	2.76E-15	4.329856
**Raet1e**	2.273812	1.58E-08	1.09E-07	4.835994	**Hrh2**	2.111783	1.16E-05	4.92E-05	4.322251
**Arhgap28**	2.273546	2.08E-06	1.00E-05	4.835101	**Gimap8**	2.109563	1.89E-06	9.15E-06	4.315604
**Dram1**	2.271119	4.68E-19	4.11E-17	4.826975	**Serping1**	2.107907	3.08E-07	1.71E-06	4.310654
**Pim1**	2.260872	8.87E-19	7.02E-17	4.792812	**Rab3ip**	2.106207	1.87E-15	5.67E-14	4.305579
**Snx10**	2.256449	3.02E-18	1.99E-16	4.778139	**Hivep3**	2.10369	2.08E-12	3.15E-11	4.298073
**Scarf1**	2.255381	1.99E-10	1.99E-09	4.774603	**Tma16**	2.09793	2.72E-11	3.27E-10	4.280947
**Il7**	2.249142	4.22E-06	1.93E-05	4.754	**Palld**	2.097851	1.75E-10	1.77E-09	4.280714
**Parp8**	2.245954	1.13E-16	4.71E-15	4.743508	**Asap3**	2.095056	5.07E-05	0.000194	4.272426
**Nampt**	2.238312	1.04E-18	7.99E-17	4.718447	**Stip1**	2.094847	1.10E-15	3.58E-14	4.27181
**Gm13889**	2.237642	0.003157	0.009061	4.716257	**Coro2a**	2.089747	2.68E-16	1.02E-14	4.256735
**Gmppb**	2.231839	1.88E-14	4.45E-13	4.697323	**Slamf7**	2.087133	3.46E-18	2.25E-16	4.24903
**Fbn1**	2.231075	3.71E-08	2.41E-07	4.694836	**Slc25a33**	2.085859	4.64E-13	8.19E-12	4.245278
**Tnc**	2.229198	0.00269	0.007814	4.688732	**Gm49339**	2.085853	4.21E-16	1.54E-14	4.24526
**Larp1b**	2.228592	1.26E-15	4.05E-14	4.686763	**Gm8797**	2.08507	5.80E-07	3.06E-06	4.242957
**Cacybp**	2.226884	1.47E-17	7.76E-16	4.68122	**Rab20**	2.07156	1.86E-17	9.61E-16	4.20341
**Rnf24**	2.222447	4.15E-12	5.91E-11	4.666844	**Lacc1**	2.071245	1.02E-14	2.58E-13	4.202491
**5-Mar**	2.219865	3.67E-16	1.36E-14	4.658499	**Itga2**	2.071075	0.001815	0.00543	4.201996
**Tcea1**	2.218504	8.85E-19	7.02E-17	4.654107	**Erap1**	2.070297	1.49E-18	1.09E-16	4.199732
**N4bp1**	2.215744	2.85E-17	1.40E-15	4.645212	**Tmtc2**	2.068341	2.94E-05	0.000117	4.194041
**Mgat4a**	2.214784	1.12E-15	3.65E-14	4.64212	**Gpr171**	2.063867	0.000467	0.001529	4.181054
**Ttc39b**	2.214587	9.02E-16	2.99E-14	4.641488	**Raet1d**	2.062185	3.24E-09	2.55E-08	4.176183
**Dnah17**	2.214514	3.21E-05	0.000127	4.641252	**Dnajb3**	2.06181	1.21E-05	5.11E-05	4.175097
**Plekhf1**	2.212813	0.000358	0.001195	4.635784	**Ppp4r2**	2.058313	7.40E-17	3.25E-15	4.164989
**Tmbim4**	2.212417	1.77E-15	5.43E-14	4.634511	**Zfp658**	2.056857	3.52E-10	3.36E-09	4.160789
**Mllt6**	2.207405	8.40E-16	2.81E-14	4.618438	**Ly86**	2.053491	8.08E-14	1.67E-12	4.151093
**Fndc3a**	2.205795	1.25E-18	9.42E-17	4.613287	**Map2k1**	2.050706	2.40E-18	1.63E-16	4.143088
**Stxbp3**	2.20171	2.02E-17	1.04E-15	4.600242	**Pttg1**	2.050335	2.26E-14	5.25E-13	4.14202
**Cd47**	2.200775	6.18E-17	2.78E-15	4.597262	**Tank**	2.045134	4.32E-17	2.04E-15	4.127117
**Trip13**	2.198986	1.84E-09	1.52E-08	4.591566	**Rab12**	2.044389	2.09E-17	1.07E-15	4.124985
**Rmdn3**	2.198589	2.53E-16	9.64E-15	4.590303	**Usp25**	2.04426	6.22E-18	3.61E-16	4.124616
**Cmtr1**	2.196432	2.23E-18	1.54E-16	4.583444	**Gm10053**	2.042866	7.88E-16	2.66E-14	4.120632
**Bend6**	2.195209	0.00256	0.00746	4.579559	**Gfpt2**	2.040502	1.15E-08	8.15E-08	4.113888
**H2-T24**	2.194696	1.03E-13	2.05E-12	4.577933	**Rnf14**	2.038164	5.16E-15	1.38E-13	4.107225
**Sqstm1**	2.193999	2.26E-17	1.14E-15	4.575721	**Tagln2**	2.034746	2.03E-18	1.40E-16	4.097506
**Tlr7**	2.193185	5.61E-18	3.34E-16	4.573141	**Ppfibp2**	2.026448	2.80E-17	1.38E-15	4.074006
**Pkp4**	2.189336	2.52E-13	4.67E-12	4.560954	**Snai1**	2.024158	0.001551	0.004689	4.067543
**Flt1**	2.18917	4.71E-06	2.14E-05	4.56043	**F3**	2.021869	3.08E-07	1.71E-06	4.061094
**Ppa1**	2.188551	2.64E-15	7.67E-14	4.558473	**Dcp2**	2.021514	6.18E-18	3.60E-16	4.060096
**Cd14**	2.188218	3.75E-16	1.39E-14	4.557421	**Igf2bp2**	2.021115	2.20E-13	4.12E-12	4.058974
**Pvr**	2.186194	2.37E-16	9.10E-15	4.551032	**Bcl3**	2.018912	1.21E-11	1.55E-10	4.052781

Gene	log2 (FC)	P-value	FDR	FC	Gene	log2 (FC)	P-value	FDR	FC
**Clic4**	2.018433	9.26E-18	5.13E-16	4.051434	**Arl4a**	1.872401	2.04E-14	4.79E-13	3.661414
**Psma3**	2.016071	1.14E-10	1.19E-09	4.044808	**Csf3r**	1.871905	3.24E-16	1.22E-14	3.660155
**Slc6a12**	2.013932	6.19E-09	4.60E-08	4.038815	**Btg3**	1.871809	5.43E-13	9.42E-12	3.659913
**Nrap**	2.007729	0.000183	0.000641	4.021486	**Grap**	1.870895	7.20E-10	6.43E-09	3.657593
**Gdf15**	2.003895	5.07E-16	1.81E-14	4.010813	**Slc11a2**	1.869082	6.51E-18	3.74E-16	3.653
**P4ha1**	2.00372	9.23E-17	3.96E-15	4.010327	**Ran**	1.868757	1.13E-16	4.71E-15	3.652178
**Cox6a2**	2.00104	2.23E-11	2.71E-10	4.002886	**Rnaseh2b**	1.866796	2.43E-15	7.12E-14	3.647217
**Hspa4l**	1.992988	4.33E-17	2.04E-15	3.980605	**Snx20**	1.865994	3.80E-16	1.40E-14	3.64519
**Cbwd1**	1.990279	5.74E-14	1.23E-12	3.97314	**Trim56**	1.865799	9.73E-15	2.47E-13	3.644698
**Smim3**	1.987829	5.07E-14	1.10E-12	3.966397	**Pnpt1**	1.857911	1.97E-15	5.90E-14	3.624825
**Zbtb5**	1.986338	7.22E-15	1.88E-13	3.9623	**Zhx2**	1.856185	7.42E-11	8.07E-10	3.62049
**Leprotl1**	1.981917	1.82E-18	1.28E-16	3.950176	**Nfe2l1**	1.854616	1.58E-16	6.40E-15	3.616555
**H2-T23**	1.981888	3.47E-15	9.73E-14	3.950098	**Zeb1**	1.851889	2.25E-06	1.07E-05	3.609726
**Acpp**	1.976945	2.03E-10	2.03E-09	3.936586	**Pgs1**	1.838461	4.13E-12	5.89E-11	3.576284
**Tlr1**	1.976362	9.17E-15	2.33E-13	3.934995	**Trim12c**	1.835129	3.87E-17	1.84E-15	3.568034
**Ccdc25**	1.97339	1.77E-15	5.43E-14	3.926897	**Asb13**	1.833157	1.25E-14	3.10E-13	3.56316
**Arid5a**	1.970093	7.98E-14	1.65E-12	3.917934	**Tmem11**	1.830894	3.07E-14	6.94E-13	3.557575
**Tmed1**	1.970012	8.72E-14	1.79E-12	3.917714	**Cept1**	1.830101	1.13E-17	6.14E-16	3.55562
**Msi2**	1.967092	3.44E-15	9.66E-14	3.909793	**Mrpl27**	1.829054	4.31E-15	1.18E-13	3.55304
**Phgdh**	1.965652	1.33E-17	7.04E-16	3.905892	**Gadd45b**	1.825414	5.73E-15	1.52E-13	3.544087
**Stard3**	1.964712	1.18E-15	3.82E-14	3.903347	**Wdr43**	1.825373	5.26E-15	1.41E-13	3.543987
**Cgas**	1.963616	9.73E-11	1.03E-09	3.900383	**Ly9**	1.825256	4.30E-16	1.56E-14	3.543699
**Mmp3**	1.96322	0.003184	0.009135	3.899313	**Rnaset2a**	1.821686	5.56E-16	1.97E-14	3.53494
**Bmp1**	1.962292	6.27E-08	3.91E-07	3.896805	**Trafd1**	1.819699	3.25E-17	1.57E-15	3.530075
**Fmnl2**	1.960192	1.84E-11	2.27E-10	3.891137	**Itga5**	1.819542	6.06E-17	2.76E-15	3.529691
**Cycs**	1.958575	2.11E-14	4.95E-13	3.886778	**Parp3**	1.819541	7.99E-14	1.65E-12	3.529688
**Tm4sf1**	1.957281	2.77E-06	1.30E-05	3.883295	**Rhobtb1**	1.815951	0.001494	0.004529	3.520917
**Il4ra**	1.955578	4.24E-13	7.54E-12	3.878712	**Cenpq**	1.815415	3.88E-07	2.11E-06	3.519608
**Ctu1**	1.955474	1.39E-14	3.42E-13	3.878435	**Vasp**	1.81158	1.28E-16	5.28E-15	3.510265
**Rassf4**	1.953013	1.21E-16	5.00E-15	3.871824	**H2-Eb1**	1.810904	3.33E-13	6.05E-12	3.508621
**Acyp2**	1.949652	1.98E-09	1.62E-08	3.862814	**Ddx39**	1.810897	3.84E-14	8.48E-13	3.508603
**Siglecg**	1.949382	0.000795	0.002512	3.86209	**Synpo2**	1.810677	0.000328	0.001102	3.508069
**Trim14**	1.946222	1.38E-14	3.40E-13	3.853641	**Ebi3**	1.807209	6.60E-14	1.39E-12	3.499645
**Gpc1**	1.945514	1.57E-13	3.03E-12	3.85175	**Ppfia1**	1.804681	6.03E-14	1.29E-12	3.493518
**Cacnb3**	1.941879	1.97E-05	8.04E-05	3.842058	**Dennd4a**	1.804478	9.36E-14	1.90E-12	3.493027
**Ttc9c**	1.939319	1.51E-16	6.14E-15	3.835246	**Dok7**	1.801867	1.75E-08	1.20E-07	3.486713
**Perm1**	1.935575	4.40E-10	4.11E-09	3.825307	**Mapkapk2**	1.801237	7.98E-17	3.48E-15	3.485189
**Dusp16**	1.935196	6.10E-16	2.13E-14	3.824302	**Trim6**	1.800141	5.56E-06	2.49E-05	3.482542
**Ptpn2**	1.934142	6.12E-17	2.77E-15	3.821507	**Srgap1**	1.799472	9.84E-06	4.23E-05	3.480928
**Ahr**	1.93339	2.48E-09	1.99E-08	3.819515	**St3gal1**	1.796651	1.91E-15	5.76E-14	3.474129
**Lilr4b**	1.931818	1.22E-16	5.03E-15	3.815356	**Zc3hav1l**	1.796414	0.000388	0.001288	3.473556
**Tnip1**	1.926627	2.91E-18	1.93E-16	3.801653	**Mllt11**	1.79397	1.12E-10	1.17E-09	3.467679
**1110038F14Rik**	1.926368	2.47E-13	4.59E-12	3.80097	**Rhbdf2**	1.793871	1.97E-14	4.65E-13	3.467441
**Prpf38a**	1.921554	1.81E-15	5.51E-14	3.788308	**Focad**	1.792528	9.17E-15	2.33E-13	3.464215
**Tpx2**	1.920408	1.15E-10	1.20E-09	3.785302	**Atxn7l1**	1.792155	1.77E-15	5.43E-14	3.463318
**Ly75**	1.914619	7.32E-16	2.50E-14	3.770143	**Arel1**	1.791515	1.86E-15	5.67E-14	3.461783
**Insl6**	1.914086	3.07E-11	3.63E-10	3.768749	**Tmem202**	1.791505	6.37E-08	3.96E-07	3.461759
**Pim3**	1.910738	1.40E-13	2.71E-12	3.760014	**Coq10b**	1.790335	2.03E-14	4.78E-13	3.458953
**Sntb1**	1.91003	2.32E-10	2.29E-09	3.758169	**Srp54b**	1.788349	0.001679	0.005046	3.454193
**Psmb6**	1.905323	5.25E-16	1.87E-14	3.745928	**Cd33**	1.785225	4.55E-16	1.64E-14	3.446722
**Irak3**	1.904618	2.66E-15	7.71E-14	3.744097	**Hip1r**	1.780351	0.000249	0.000855	3.435098
**Etnk1**	1.900533	9.80E-17	4.17E-15	3.733511	**Col4a2**	1.778045	2.44E-06	1.16E-05	3.429612
**Klra2**	1.897923	1.58E-05	6.53E-05	3.726764	**Nme6**	1.776677	1.42E-10	1.46E-09	3.42636
**Rcl1**	1.896199	1.81E-14	4.32E-13	3.722312	**Hsp90aa1**	1.775146	1.03E-12	1.68E-11	3.422726
**Ralgps1**	1.89434	1.44E-09	1.21E-08	3.717518	**Dnajc1**	1.773759	8.16E-16	2.73E-14	3.419437
**Mmp28**	1.894005	0.000215	0.000747	3.716656	**Flywch2**	1.773363	1.54E-05	6.41E-05	3.4185
**Vasn**	1.892768	1.17E-10	1.21E-09	3.713471	**Gabpb1**	1.771223	7.84E-15	2.02E-13	3.413431
**Pik3ip1**	1.885949	2.50E-05	0.0001	3.69596	**Lzts2**	1.771018	2.70E-12	4.00E-11	3.412947
**Ccdc144b**	1.883259	0.002745	0.007962	3.689075	**Trpm4**	1.768073	1.25E-12	2.01E-11	3.405987
**Il17ra**	1.880644	1.61E-08	1.11E-07	3.682394	**Grina**	1.764522	1.61E-16	6.47E-15	3.397615
**Stk40**	1.877164	6.34E-16	2.20E-14	3.673522	**Tmem140**	1.762779	6.65E-15	1.74E-13	3.393513
**Cyp1b1**	1.876031	3.09E-08	2.03E-07	3.670639	**Il2rg**	1.761735	2.79E-15	8.04E-14	3.391058
**Gas7**	1.875863	1.81E-16	7.20E-15	3.670212	**Dyrk2**	1.757515	5.03E-15	1.36E-13	3.381152
**Zfp719**	1.874664	1.82E-12	2.79E-11	3.667161	**Camkk1**	1.755658	0.000801	0.002529	3.376803
**Plk3**	1.873062	1.14E-07	6.77E-07	3.663093	**Hspa9**	1.754201	2.81E-15	8.08E-14	3.373394

Gene	log2 (FC)	P-value	FDR	FC	Gene	log2 (FC)	P-value	FDR	FC
**Max**	1.752095	8.50E-16	2.83E-14	3.368473	**P4ha2**	1.619358	5.85E-07	3.09E-06	3.072382
**Fbxw17**	1.750757	3.49E-10	3.33E-09	3.365351	**Phldb1**	1.617008	9.84E-11	1.04E-09	3.067383
**Alas1**	1.749748	6.75E-15	1.77E-13	3.362998	**Them4**	1.615026	4.86E-11	5.51E-10	3.063172
**Kcna3**	1.749529	0.000205	0.000714	3.362487	**Olfr571**	1.60997	3.77E-06	1.74E-05	3.052454
**Ascc3**	1.749023	9.82E-17	4.18E-15	3.36131	**Steap2**	1.609622	0.000583	0.00188	3.051718
**Kars**	1.746488	1.05E-16	4.41E-15	3.355407	**Mllt3**	1.607167	7.09E-12	9.51E-11	3.04653
**Gsdmd**	1.742669	1.43E-15	4.51E-14	3.346537	**Stk38l**	1.606528	4.50E-09	3.43E-08	3.045182
**Rrs1**	1.738238	1.85E-14	4.38E-13	3.336276	**Irf8**	1.604493	1.22E-12	1.96E-11	3.040888
**Top1mt**	1.737121	5.20E-10	4.78E-09	3.333691	**Psmc2**	1.60246	5.96E-15	1.57E-13	3.036606
**Rhbdf1**	1.736935	0.002238	0.006587	3.333263	**Cables2**	1.601252	2.72E-13	5.01E-12	3.034065
**Tmem229b**	1.730109	3.11E-14	7.00E-13	3.317528	**Psma7**	1.600112	7.43E-14	1.55E-12	3.031669
**Ube2m**	1.728917	8.43E-16	2.81E-14	3.314789	**Katna1**	1.599426	5.31E-13	9.22E-12	3.030227
**Tmem171**	1.72763	1.47E-05	6.15E-05	3.311834	**Tlk2**	1.598839	8.47E-13	1.42E-11	3.028995
**Gpd2**	1.727242	4.47E-16	1.62E-14	3.310942	**Klf4**	1.598415	6.39E-11	7.04E-10	3.028105
**Crem**	1.721809	1.21E-12	1.95E-11	3.298498	**Cybc1**	1.597965	1.14E-16	4.73E-15	3.027161
**Apool**	1.721784	8.42E-14	1.73E-12	3.29844	**Mafk**	1.596759	1.37E-15	4.34E-14	3.024631
**Dcaf1**	1.720344	3.77E-15	1.04E-13	3.295149	**Cox18**	1.596702	6.54E-08	4.06E-07	3.024512
**Sdc4**	1.719407	5.91E-13	1.02E-11	3.293009	**Rffl**	1.596389	5.09E-14	1.10E-12	3.023854
**Coa5**	1.718273	4.26E-16	1.55E-14	3.290422	**Larp1**	1.596373	8.13E-15	2.09E-13	3.023822
**Fhl3**	1.718165	6.75E-12	9.08E-11	3.290176	**Gsto1**	1.596327	7.42E-13	1.25E-11	3.023726
**Lmtk2**	1.716778	1.49E-09	1.25E-08	3.287014	**Pgap2**	1.592618	4.74E-13	8.36E-12	3.015962
**Nlgn2**	1.716775	4.23E-06	1.93E-05	3.287009	**Lgals8**	1.590866	4.11E-16	1.51E-14	3.012302
**Acsl5**	1.71553	7.08E-17	3.15E-15	3.284173	**Mob3b**	1.588828	3.85E-10	3.65E-09	3.008048
**Asah2**	1.714804	9.55E-14	1.93E-12	3.28252	**Cdh5**	1.588768	0.003252	0.009316	3.007924
**Ampd3**	1.713947	4.39E-13	7.79E-12	3.280571	**B2m**	1.588493	7.78E-14	1.62E-12	3.00735
**Elp5**	1.713588	8.36E-14	1.73E-12	3.279755	**Dhps**	1.585045	3.52E-14	7.83E-13	3.000172
**Pbxip1**	1.711163	2.19E-15	6.50E-14	3.274246	**Psma4**	1.58328	4.46E-15	1.21E-13	2.996503
**Gabbr1**	1.710298	9.53E-05	0.000349	3.272283	**Kif1a**	1.581832	8.86E-05	0.000326	2.993498
**Angptl6**	1.708916	4.96E-09	3.76E-08	3.269152	**Cav1**	1.580665	1.03E-07	6.18E-07	2.991076
**Ramp3**	1.708886	0.001879	0.005604	3.269084	**Ilrun**	1.580466	4.92E-17	2.29E-15	2.990664
**Mthfr**	1.708625	6.30E-11	6.96E-10	3.268492	**Rassf1**	1.578107	1.20E-12	1.93E-11	2.985779
**Mdm2**	1.708216	5.94E-15	1.57E-13	3.267566	**Rasgef1b**	1.577874	4.36E-15	1.19E-13	2.985297
**Mocs2**	1.703885	3.45E-15	9.69E-14	3.25777	**Cmklr1**	1.577853	1.20E-11	1.55E-10	2.985252
**Dst**	1.692229	7.84E-09	5.69E-08	3.231555	**Plek**	1.577322	9.15E-15	2.33E-13	2.984153
**Amn1**	1.684504	3.68E-13	6.62E-12	3.214299	**Bak1**	1.5741	2.76E-15	7.98E-14	2.977498
**Fcf1**	1.681823	4.45E-13	7.88E-12	3.208331	**Tmco3**	1.570991	2.45E-15	7.18E-14	2.971088
**Mrpl53**	1.681195	4.72E-12	6.64E-11	3.206935	**St6galnac4**	1.569154	1.22E-15	3.95E-14	2.967306
**Adgre1**	1.679633	2.18E-16	8.45E-15	3.203465	**Macroh2a1**	1.567104	1.37E-15	4.34E-14	2.963094
**Akna**	1.676191	4.90E-14	1.07E-12	3.195831	**Rab38**	1.567028	0.0006	0.00193	2.962938
**Asf1a**	1.676151	4.23E-13	7.54E-12	3.195741	**Rela**	1.566474	2.75E-15	7.96E-14	2.961799
**Ubb**	1.673096	5.53E-15	1.47E-13	3.188981	**Phlda1**	1.566112	2.17E-05	8.82E-05	2.961056
**Ube2e2**	1.671092	4.66E-07	2.50E-06	3.184557	**Tbk1**	1.564439	1.64E-16	6.59E-15	2.957625
**Psmd11**	1.669669	9.78E-16	3.22E-14	3.181416	**Fam72a**	1.56342	0.001138	0.00351	2.955535
**Dbn1**	1.66559	0.000895	0.002806	3.172433	**Ifnar2**	1.563148	3.80E-15	1.05E-13	2.95498
**Psma2**	1.664728	2.09E-13	3.93E-12	3.170538	**Psmd4**	1.560384	3.56E-15	9.95E-14	2.949323
**Asns**	1.664572	2.87E-16	1.09E-14	3.170195	**Tfpi2**	1.558218	0.000894	0.002802	2.944898
**Ccdc88b**	1.660622	1.27E-15	4.09E-14	3.161528	**Inpp5b**	1.557321	3.54E-15	9.89E-14	2.943069
**Zfp593**	1.658004	1.29E-11	1.64E-10	3.155796	**Tmem128**	1.554484	2.77E-13	5.08E-12	2.937287
**Aars**	1.65739	1.80E-16	7.18E-15	3.154454	**Thbs1**	1.553114	2.97E-10	2.87E-09	2.934498
**Mgst2**	1.653992	0.000508	0.001655	3.147033	**Themis2**	1.551905	4.97E-11	5.61E-10	2.932041
**Ubr4**	1.652667	1.09E-13	2.16E-12	3.144142	**Camsap1**	1.551267	1.35E-08	9.44E-08	2.930743
**Pola2**	1.651777	1.70E-12	2.64E-11	3.142203	**Itpr2**	1.548896	9.48E-12	1.24E-10	2.925931
**Mid1**	1.645609	8.48E-09	6.12E-08	3.128799	**Slc39a14**	1.548187	3.51E-12	5.07E-11	2.924494
**Arl5c**	1.645248	5.73E-12	7.87E-11	3.128015	**Slc12a4**	1.546931	6.39E-16	2.21E-14	2.921949
**P2ry14**	1.642237	1.48E-12	2.33E-11	3.121496	**Peli1**	1.546878	9.27E-14	1.88E-12	2.921842
**Plk2**	1.640947	2.46E-14	5.67E-13	3.118705	**Hspa1l**	1.546022	1.05E-05	4.47E-05	2.920108
**Ugcg**	1.640749	1.32E-15	4.21E-14	3.118276	**Chpf**	1.545571	8.49E-09	6.13E-08	2.919196
**Lipg**	1.638095	0.001621	0.004886	3.112545	**Nfu1**	1.544504	3.68E-15	1.03E-13	2.917037
**Ttc39c**	1.637812	1.94E-10	1.94E-09	3.111934	**Ears2**	1.544473	1.16E-07	6.91E-07	2.916974
**Stat3**	1.637265	1.04E-16	4.38E-15	3.110755	**Ccr5**	1.542997	3.58E-11	4.17E-10	2.913992
**Prrx1**	1.633724	0.000864	0.002714	3.10313	**Slx4ip**	1.541489	6.32E-11	6.98E-10	2.910948
**Txnl1**	1.63243	9.87E-16	3.23E-14	3.100347	**Ccnyl1**	1.537965	2.31E-12	3.46E-11	2.903845
**Olfr735**	1.63073	1.62E-07	9.38E-07	3.096697	**Slc30a6**	1.536853	1.55E-10	1.58E-09	2.901608
**Jam2**	1.628656	0.000236	0.000812	3.092247	**Gca**	1.536209	1.74E-12	2.69E-11	2.900313
**Slc7a11**	1.626579	7.96E-11	8.61E-10	3.087799	**Trim13**	1.531832	2.13E-12	3.22E-11	2.891528

Gene	log2 (FC)	P-value	FDR	FC	Gene	log2 (FC)	P-value	FDR	FC
**Ankib1**	1.5304	1.42E-14	3.47E-13	2.888659	**Hspa8**	1.421567	2.26E-13	4.21E-12	2.678763
**Tmbim6**	1.529352	8.16E-16	2.73E-14	2.886561	**Fam177a**	1.420259	6.09E-13	1.04E-11	2.676336
**Pced1b**	1.524724	2.40E-06	1.14E-05	2.877318	**Ube2f**	1.4198	1.00E-13	2.01E-12	2.675483
**Rbm7**	1.522133	2.99E-15	8.57E-14	2.872155	**Cenpj**	1.417759	1.93E-10	1.93E-09	2.671701
**Eno2**	1.519413	4.26E-12	6.05E-11	2.866745	**Gnb4**	1.416829	4.07E-05	0.000158	2.669981
**Lrrc51**	1.516106	9.82E-05	0.000359	2.860179	**Yjefn3**	1.416398	0.001577	0.004761	2.669183
**Gja1**	1.513333	9.23E-06	3.98E-05	2.854687	**Noc4l**	1.413473	7.05E-12	9.46E-11	2.663776
**Sirt6**	1.51081	9.10E-12	1.20E-10	2.849699	**Pi4k2b**	1.413016	9.06E-14	1.84E-12	2.662933
**1810055G02Rik**	1.509071	1.12E-07	6.66E-07	2.846266	**Tdrd7**	1.41231	1.62E-14	3.93E-13	2.661629
**Cct3**	1.508706	2.21E-14	5.17E-13	2.845548	**Ntmt1**	1.407949	1.80E-09	1.49E-08	2.653597
**Tent5a**	1.508656	2.40E-15	7.05E-14	2.845449	**Uchl1**	1.407947	0.00131	0.003998	2.653593
**Aldh1b1**	1.503068	2.47E-08	1.65E-07	2.834449	**Atp6v0b**	1.407605	1.68E-13	3.23E-12	2.652964
**Tnfrsf26**	1.501268	1.71E-10	1.74E-09	2.830914	**Tmem209**	1.407222	1.18E-12	1.91E-11	2.65226
**Fkbp5**	1.501221	1.10E-08	7.80E-08	2.830821	**Psmd12**	1.406748	1.86E-13	3.55E-12	2.651389
**Vmn2r108**	1.498838	0.000633	0.002029	2.826149	**Hsp90ab1**	1.404593	2.60E-14	5.95E-13	2.647431
**Prr5l**	1.497581	1.12E-09	9.64E-09	2.823688	**Timm17a**	1.403494	3.02E-12	4.42E-11	2.645416
**Tmbim1**	1.497551	8.58E-16	2.85E-14	2.82363	**Slc3a2**	1.403251	3.29E-15	9.31E-14	2.644969
**Faap24**	1.495469	3.43E-11	4.01E-10	2.819558	**Adap2**	1.399866	6.33E-10	5.73E-09	2.638771
**Trim26**	1.491587	2.44E-14	5.63E-13	2.811982	**Eif4e**	1.399336	9.79E-14	1.97E-12	2.637801
**Bst1**	1.491284	1.40E-12	2.21E-11	2.811391	**Lonp1**	1.39894	1.84E-14	4.37E-13	2.637078
**Rrp9**	1.491095	5.19E-13	9.04E-12	2.811023	**Sh3bp4**	1.398065	1.70E-09	1.41E-08	2.635478
**Mrps10**	1.489449	3.56E-12	5.13E-11	2.807818	**Rnf145**	1.39703	8.48E-13	1.42E-11	2.633589
**Nfil3**	1.488883	1.21E-10	1.26E-09	2.806716	**Mrpl52**	1.395983	1.34E-11	1.70E-10	2.631678
**Wnt9a**	1.488714	8.95E-05	0.00033	2.806387	**Ptges2**	1.394928	1.10E-12	1.79E-11	2.629754
**Diablo**	1.488433	3.83E-13	6.89E-12	2.805841	**Ankrd24**	1.393925	1.11E-07	6.62E-07	2.627927
**Plpp5**	1.487311	5.96E-08	3.73E-07	2.80366	**Rgs14**	1.392772	1.93E-09	1.58E-08	2.625828
**Arv1**	1.486638	8.01E-08	4.89E-07	2.802351	**Zc3hav1**	1.392202	2.01E-15	6.01E-14	2.62479
**Gtpbp6**	1.484804	8.77E-13	1.46E-11	2.798791	**Atp13a1**	1.391659	1.98E-13	3.74E-12	2.623802
**St7**	1.483548	4.88E-08	3.10E-07	2.796356	**Tyw5**	1.390329	3.49E-08	2.28E-07	2.621385
**Vav1**	1.479421	4.82E-15	1.30E-13	2.788367	**Eme2**	1.3883	1.84E-09	1.51E-08	2.6177
**Mecp2**	1.478857	3.81E-13	6.86E-12	2.787278	**Rars**	1.387346	6.09E-15	1.60E-13	2.61597
**Prkrip1**	1.474828	1.18E-10	1.23E-09	2.779504	**Nectin2**	1.386978	1.66E-07	9.60E-07	2.615302
**Pdlim7**	1.465993	1.05E-06	5.25E-06	2.762535	**Dnaja1**	1.385002	4.07E-15	1.12E-13	2.611723
**Ascc2**	1.464583	3.46E-14	7.70E-13	2.759837	**Psmc5**	1.38485	1.37E-14	3.38E-13	2.611448
**Polr3d**	1.464569	6.82E-13	1.16E-11	2.759809	**Sh3bgrl**	1.382924	1.71E-10	1.73E-09	2.607964
**Eif2b2**	1.464033	4.03E-14	8.88E-13	2.758785	**H2-DMa**	1.38033	2.78E-09	2.21E-08	2.60328
**Stk39**	1.462766	5.11E-06	2.31E-05	2.756364	**Cox17**	1.375473	1.48E-11	1.86E-10	2.59453
**Orai2**	1.461087	1.75E-12	2.69E-11	2.753157	**Phlpp1**	1.375412	6.44E-12	8.72E-11	2.594419
**B9d2**	1.460601	2.69E-12	3.99E-11	2.75223	**Cks2**	1.373667	1.01E-06	5.07E-06	2.591283
**Sell**	1.460144	0.000301	0.001019	2.751357	**Tasor2**	1.372484	4.54E-12	6.44E-11	2.58916
**Trib1**	1.460103	4.61E-06	2.09E-05	2.751279	**Fbxo7**	1.37216	2.71E-13	5.00E-12	2.588579
**Stau2**	1.459321	3.74E-11	4.35E-10	2.749789	**Rnf139**	1.371531	7.11E-14	1.49E-12	2.58745
**Olfr539**	1.459303	1.51E-10	1.54E-09	2.749754	**Rfc3**	1.370566	5.38E-10	4.93E-09	2.58572
**Mmp9**	1.458192	6.88E-06	3.03E-05	2.747638	**Psmd7**	1.369416	4.53E-15	1.23E-13	2.58366
**Ints12**	1.456663	1.90E-13	3.61E-12	2.744727	**Tdg**	1.368932	3.42E-13	6.20E-12	2.582792
**Zyx**	1.456277	5.42E-09	4.07E-08	2.743994	**Wdr74**	1.368662	4.75E-12	6.67E-11	2.58231
**Dbnl**	1.455012	6.65E-14	1.40E-12	2.741588	**Itprip**	1.367723	3.48E-11	4.06E-10	2.580629
**Timm10**	1.450984	1.62E-11	2.01E-10	2.733944	**Casp3**	1.365532	6.53E-11	7.18E-10	2.576713
**Fam241a**	1.450169	6.19E-15	1.63E-13	2.732401	**Col20a1**	1.364408	0.000564	0.001823	2.574706
**Smg7**	1.448778	1.09E-12	1.78E-11	2.729767	**Mrpl32**	1.361647	2.20E-10	2.18E-09	2.569783
**Ubtd2**	1.445549	9.71E-06	4.17E-05	2.723665	**Mill2**	1.361465	1.31E-06	6.48E-06	2.569461
**Acot7**	1.445281	2.40E-11	2.90E-10	2.723159	**Tm9sf4**	1.361392	3.08E-15	8.78E-14	2.56933
**Esd**	1.445153	1.64E-13	3.16E-12	2.722916	**Flnb**	1.35898	9.33E-12	1.22E-10	2.565037
**Fzr1**	1.444569	8.70E-10	7.67E-09	2.721815	**Ddhd1**	1.356928	2.38E-13	4.44E-12	2.561393
**Chmp4b**	1.436421	4.97E-14	1.08E-12	2.706487	**Ube2s**	1.356721	8.40E-14	1.73E-12	2.561024
**Cnih4**	1.436055	2.24E-12	3.37E-11	2.7058	**Ppie**	1.355984	5.66E-12	7.81E-11	2.559717
**Ddx24**	1.430695	9.80E-16	3.22E-14	2.695765	**Ctsc**	1.354972	5.07E-13	8.87E-12	2.557921
**Traf6**	1.430496	4.97E-11	5.62E-10	2.695393	**Adprh**	1.354824	8.43E-14	1.73E-12	2.557659
**Pxk**	1.429705	4.09E-13	7.30E-12	2.693916	**Fgl2**	1.354243	1.32E-08	9.23E-08	2.556629
**Plscr2**	1.428793	0.000521	0.001693	2.692214	**Znrd2**	1.353672	3.95E-09	3.05E-08	2.555618
**Pomp**	1.427902	2.39E-13	4.44E-12	2.690551	**Ssr2**	1.353585	1.41E-12	2.24E-11	2.555463
**Rnf11**	1.427282	3.61E-14	8.03E-13	2.689395	**Sra1**	1.352985	4.89E-10	4.52E-09	2.554401
**Rrp7a**	1.426063	2.24E-15	6.60E-14	2.687125	**Psat1**	1.352738	2.84E-15	8.16E-14	2.553964
**Zcchc2**	1.424699	4.75E-15	1.29E-13	2.684586	**Vmn1r233**	1.35212	2.01E-05	8.19E-05	2.552869
**Adgra2**	1.423189	0.000804	0.002538	2.681776	**Dusp1**	1.351309	1.88E-09	1.55E-08	2.551435

Gene	log2 (FC)	P-value	FDR	FC	Gene	log2 (FC)	P-value	FDR	FC
**St3gal3**	1.350055	1.33E-11	1.68E-10	2.549219	**Rhbdd1**	1.281416	2.84E-14	6.46E-13	2.430774
**Ube2e1**	1.348873	3.85E-12	5.51E-11	2.547132	**Pwp1**	1.281289	1.60E-12	2.49E-11	2.43056
**Mt2**	1.348573	2.82E-08	1.87E-07	2.5466	**Furin**	1.281091	8.62E-11	9.27E-10	2.430227
**Ribc1**	1.347842	0.003451	0.009848	2.545312	**Rpf2**	1.280737	6.12E-11	6.79E-10	2.429631
**Med13**	1.345388	1.22E-13	2.41E-12	2.540986	**Sav1**	1.280592	1.61E-13	3.10E-12	2.429386
**Cemip2**	1.345248	3.56E-12	5.13E-11	2.540739	**Dpf2**	1.279038	1.06E-10	1.12E-09	2.426771
**Vps37c**	1.34471	1.65E-12	2.56E-11	2.539791	**Kansl3**	1.278896	1.04E-12	1.70E-11	2.426531
**Olfr509**	1.344533	0.000197	0.000687	2.53948	**Srsf7**	1.278783	5.43E-12	7.52E-11	2.426342
**Map4**	1.3417	9.04E-14	1.84E-12	2.534497	**Psmb4**	1.278227	1.68E-12	2.61E-11	2.425407
**Nup43**	1.33989	1.40E-06	6.89E-06	2.531321	**Chil1**	1.274085	0.00222	0.006538	2.418454
**Taf7**	1.339596	1.93E-10	1.94E-09	2.530805	**Tmem219**	1.273869	1.42E-11	1.79E-10	2.418091
**Pkdcc**	1.339447	2.45E-10	2.41E-09	2.530542	**Lmbr1l**	1.272575	2.35E-10	2.31E-09	2.415924
**Keap1**	1.3386	6.86E-13	1.16E-11	2.529057	**Psmc4**	1.270796	1.21E-13	2.39E-12	2.412946
**Vps54**	1.338583	5.72E-14	1.23E-12	2.529027	**Man2a1**	1.270697	1.89E-13	3.58E-12	2.41278
**Lims1**	1.336828	2.88E-14	6.54E-13	2.525953	**Cct7**	1.270551	2.72E-13	5.02E-12	2.412536
**Pno1**	1.336587	2.53E-12	3.77E-11	2.525531	**Gpbp1**	1.268427	1.16E-13	2.29E-12	2.408988
**Slc2a1**	1.336428	8.15E-15	2.10E-13	2.525253	**Ppp1r11**	1.265766	5.84E-12	7.99E-11	2.404549
**1810037I17Rik**	1.336329	4.72E-10	4.38E-09	2.52508	**Rwdd1**	1.26444	5.64E-12	7.78E-11	2.40234
**Shf**	1.336198	1.20E-05	5.08E-05	2.524851	**Prelid3b**	1.262564	2.13E-13	4.00E-12	2.399217
**Klhl38**	1.335974	0.000103	0.000375	2.524459	**Tmem221**	1.261805	0.000266	0.000908	2.397955
**Bcam**	1.33554	1.23E-06	6.13E-06	2.523699	**Mtmr14**	1.260107	1.02E-13	2.05E-12	2.395135
**Opa3**	1.335101	1.21E-13	2.38E-12	2.522932	**Gng5**	1.258311	1.60E-11	1.99E-10	2.392156
**Lnpk**	1.334746	5.60E-11	6.26E-10	2.522311	**Mif**	1.257345	1.31E-12	2.09E-11	2.390554
**Osmr**	1.33465	0.000321	0.001081	2.522143	**Tut7**	1.257096	2.39E-14	5.53E-13	2.390142
**Nubp1**	1.333795	2.76E-14	6.28E-13	2.520649	**Rrad**	1.257012	1.18E-05	5.00E-05	2.390002
**Chordc1**	1.331296	6.87E-14	1.44E-12	2.516286	**Zfp446**	1.256112	5.79E-06	2.59E-05	2.388512
**Rgcc**	1.3312	1.32E-08	9.27E-08	2.51612	**Sap30**	1.253949	8.75E-12	1.16E-10	2.384934
**Grk4**	1.329671	1.26E-07	7.44E-07	2.513453	**Cul2**	1.253019	1.72E-09	1.43E-08	2.383397
**Lztfl1**	1.327909	4.02E-12	5.74E-11	2.510385	**Msn**	1.250315	7.46E-14	1.55E-12	2.378933
**Wdr59**	1.326764	8.33E-12	1.10E-10	2.508394	**Surf4**	1.249525	7.00E-15	1.83E-13	2.377631
**Dclre1c**	1.324614	1.04E-11	1.36E-10	2.504659	**9930111J21Rik1**	1.24843	9.17E-10	8.05E-09	2.375827
**Ift57**	1.323893	4.67E-10	4.34E-09	2.503407	**Rxylt1**	1.248101	3.74E-12	5.37E-11	2.375286
**Snrnp35**	1.323081	5.06E-11	5.71E-10	2.501999	**Samsn1**	1.245137	1.44E-10	1.48E-09	2.37041
**Cpd**	1.322999	2.36E-13	4.40E-12	2.501857	**Ppp3cc**	1.244982	8.05E-07	4.13E-06	2.370156
**Slc7a1**	1.320817	4.63E-13	8.18E-12	2.498075	**Gtf3c5**	1.244955	1.53E-11	1.91E-10	2.370111
**Olfr46**	1.32077	3.84E-07	2.09E-06	2.497994	**Cd200r4**	1.24475	2.40E-12	3.58E-11	2.369774
**Ddit4**	1.320664	1.64E-07	9.49E-07	2.49781	**Nmd3**	1.244713	7.39E-10	6.58E-09	2.369714
**Spsb1**	1.318687	9.23E-06	3.98E-05	2.49439	**Snw1**	1.244622	3.29E-13	5.98E-12	2.369564
**Trim25**	1.317426	1.96E-15	5.89E-14	2.49221	**Ciapin1**	1.243853	2.13E-12	3.21E-11	2.368303
**Hat1**	1.314669	3.08E-13	5.64E-12	2.487452	**Cers6**	1.241623	9.04E-14	1.84E-12	2.364645
**Kri1**	1.313999	6.62E-14	1.39E-12	2.486298	**Pnpla1**	1.241098	0.000423	0.001391	2.363784
**Slirp**	1.313894	1.96E-10	1.96E-09	2.486117	**AI837181**	1.239695	3.89E-13	6.97E-12	2.361486
**Ms4a8a**	1.311565	8.59E-06	3.72E-05	2.482107	**Ist1**	1.239053	8.22E-14	1.70E-12	2.360436
**Dtx2**	1.310162	1.24E-11	1.59E-10	2.479693	**Gng12**	1.238859	6.36E-14	1.34E-12	2.360117
**Mpzl3**	1.306371	7.13E-05	0.000266	2.473186	**Tgif1**	1.238601	1.26E-14	3.13E-13	2.359695
**Tbc1d9**	1.304864	4.02E-13	7.19E-12	2.470604	**Smg5**	1.237752	9.52E-14	1.92E-12	2.358307
**Tor1aip1**	1.301881	6.34E-14	1.34E-12	2.465501	**Rps5**	1.237447	6.20E-11	6.87E-10	2.357809
**Csf2rb**	1.300943	4.13E-14	9.07E-13	2.4639	**Cnot4**	1.236359	4.61E-12	6.53E-11	2.356032
**Mthfd2**	1.299297	6.20E-14	1.32E-12	2.461089	**Hectd4**	1.235706	2.07E-09	1.69E-08	2.354966
**Fignl2**	1.298211	5.02E-11	5.67E-10	2.459237	**Zranb2**	1.235501	8.36E-11	9.02E-10	2.354631
**Mtdh**	1.297637	4.18E-15	1.15E-13	2.458259	**Urm1**	1.234714	2.89E-12	4.24E-11	2.353347
**Tbc1d13**	1.296046	5.35E-12	7.42E-11	2.45555	**Gfpt1**	1.234119	2.72E-14	6.21E-13	2.352377
**Hax1**	1.29564	2.79E-12	4.13E-11	2.454858	**Itgav**	1.233473	2.31E-08	1.55E-07	2.351324
**Ift22**	1.294424	5.23E-11	5.89E-10	2.452791	**Bclaf3**	1.233435	2.11E-08	1.43E-07	2.351261
**Thyn1**	1.293097	3.41E-10	3.26E-09	2.450535	**Ciao2b**	1.231693	6.93E-13	1.17E-11	2.348425
**Hspd1**	1.292569	5.62E-13	9.73E-12	2.449638	**Mrpl54**	1.231413	2.18E-10	2.16E-09	2.347969
**Jdp2**	1.292394	6.36E-10	5.74E-09	2.449342	**Rapgef2**	1.230502	7.22E-11	7.88E-10	2.346486
**Tmem199**	1.290803	6.38E-13	1.09E-11	2.446641	**3830406C13Rik**	1.229954	1.30E-11	1.66E-10	2.345595
**Mocs1**	1.288059	9.95E-13	1.63E-11	2.441993	**Usp42**	1.229945	9.80E-10	8.54E-09	2.34558
**Psmb7**	1.287715	1.47E-13	2.84E-12	2.441411	**Mettl1**	1.228963	5.72E-09	4.29E-08	2.343984
**Psmb3**	1.287028	9.03E-14	1.84E-12	2.440248	**Spata5**	1.228463	5.84E-10	5.31E-09	2.343172
**Srpr**	1.286229	2.52E-14	5.79E-13	2.438897	**Psmd3**	1.228238	8.13E-15	2.09E-13	2.342807
**Timm23**	1.285858	5.94E-13	1.02E-11	2.438271	**Psme2b**	1.227355	4.95E-08	3.14E-07	2.341373
**Tal1**	1.285838	4.96E-07	2.65E-06	2.438237	**Psma6**	1.225195	6.61E-13	1.13E-11	2.33787
**Eif3c**	1.283808	1.27E-13	2.49E-12	2.434808	**Yrdc**	1.223448	2.84E-10	2.76E-09	2.335041

Gene	log2 (FC)	P-value	FDR	FC	Gene	log2 (FC)	P-value	FDR	FC
**Plagl2**	1.222645	1.98E-12	3.02E-11	2.333741	**Reps1**	1.159638	6.56E-10	5.90E-09	2.234013
**Fcer1g**	1.221314	1.09E-12	1.78E-11	2.331589	**Eif2s1**	1.157963	1.48E-12	2.33E-11	2.231421
**Mrpl13**	1.219885	7.18E-11	7.84E-10	2.329281	**Papss2**	1.157218	3.95E-08	2.55E-07	2.230269
**Pole4**	1.219244	4.66E-12	6.56E-11	2.328247	**Slc30a4**	1.155815	2.90E-07	1.61E-06	2.228101
**Tmsb10**	1.218544	2.47E-10	2.43E-09	2.327118	**Nbas**	1.155702	1.84E-11	2.26E-10	2.227928
**Tra2a**	1.218536	1.41E-09	1.19E-08	2.327104	**Mrpl24**	1.155337	4.63E-12	6.54E-11	2.227364
**Prkce**	1.218484	1.54E-06	7.52E-06	2.327021	**Ndufs4**	1.151685	3.41E-09	2.67E-08	2.221732
**Tmem120b**	1.218044	1.33E-09	1.13E-08	2.326311	**Pdzd11**	1.148342	9.72E-11	1.03E-09	2.21659
**Ak2**	1.217098	1.30E-12	2.07E-11	2.324786	**Zcrb1**	1.148114	8.92E-11	9.54E-10	2.21624
**Trim5**	1.216531	1.69E-09	1.40E-08	2.323872	**Morc3**	1.148066	2.97E-13	5.43E-12	2.216166
**Fbrs**	1.216212	3.46E-10	3.31E-09	2.323358	**Ktn1**	1.148005	5.68E-10	5.17E-09	2.216072
**Rpl24**	1.215421	2.54E-11	3.06E-10	2.322085	**Amz2**	1.147638	9.42E-13	1.56E-11	2.215508
**4930453N24Rik**	1.215234	8.65E-12	1.14E-10	2.321785	**Mrpl35**	1.144702	1.49E-10	1.53E-09	2.211005
**Mcm9**	1.211731	2.76E-11	3.30E-10	2.316153	**Ranbp1**	1.143411	4.26E-11	4.90E-10	2.209026
**Mfhas1**	1.209801	3.02E-09	2.39E-08	2.313057	**Eif2s2**	1.143251	4.45E-12	6.31E-11	2.208782
**Uri1**	1.209636	7.17E-12	9.60E-11	2.312792	**Cr1l**	1.142079	6.68E-13	1.14E-11	2.206988
**Metap1**	1.209342	1.55E-12	2.43E-11	2.312322	**Col4a1**	1.141057	0.000162	0.000574	2.205426
**Cd164**	1.208652	9.77E-13	1.61E-11	2.311215	**Ctsz**	1.140765	1.79E-12	2.76E-11	2.204979
**Ubxn2a**	1.208169	5.78E-09	4.32E-08	2.310442	**Slc18a1**	1.140551	3.32E-06	1.54E-05	2.204652
**Hmmr**	1.206489	0.000156	0.000552	2.307753	**Isca2**	1.138969	1.49E-10	1.53E-09	2.202236
**Vta1**	1.205907	6.49E-10	5.85E-09	2.306822	**Rasa4**	1.138232	1.29E-11	1.64E-10	2.201111
**Arid5b**	1.205062	8.77E-11	9.40E-10	2.305472	**Lyrm1**	1.138128	2.16E-08	1.46E-07	2.200953
**Dusp6**	1.203441	3.59E-08	2.33E-07	2.302883	**Slc20a1**	1.136196	3.65E-11	4.24E-10	2.198006
**Nemp1**	1.203187	3.68E-08	2.40E-07	2.302477	**Acot9**	1.136041	4.17E-12	5.93E-11	2.19777
**Psmd14**	1.20259	4.08E-13	7.29E-12	2.301525	**Ifi207**	1.135706	8.89E-14	1.82E-12	2.197261
**Polr2c**	1.201998	5.96E-13	1.03E-11	2.300581	**Zdhhc5**	1.135195	3.27E-13	5.96E-12	2.196482
**Tmem173**	1.200893	2.02E-09	1.65E-08	2.298819	**Scnm1**	1.135165	4.66E-09	3.55E-08	2.196436
**Aldh1a2**	1.199431	1.44E-08	1.01E-07	2.296491	**Zwint**	1.134513	1.88E-13	3.58E-12	2.195444
**Nutf2**	1.194131	9.49E-05	0.000348	2.28807	**Psmb2**	1.134472	1.62E-12	2.52E-11	2.195381
**9930111J21Rik2**	1.193187	1.91E-12	2.92E-11	2.286572	**Aen**	1.134245	5.09E-11	5.73E-10	2.195036
**Rbis**	1.193143	3.16E-10	3.04E-09	2.286503	**Trip10**	1.132607	1.04E-10	1.10E-09	2.192547
**Dnajc21**	1.192594	3.51E-11	4.10E-10	2.285634	**Mrps18a**	1.130383	3.09E-12	4.51E-11	2.189169
**Ptprj**	1.19253	5.13E-12	7.17E-11	2.285531	**Chmp2a**	1.128536	1.02E-11	1.33E-10	2.186368
**Zbtb3**	1.192353	2.30E-06	1.10E-05	2.285252	**Rbbp8**	1.12744	9.60E-11	1.02E-09	2.184707
**Usp12**	1.19034	6.07E-14	1.29E-12	2.282064	**Eif3j2**	1.125725	2.58E-09	2.07E-08	2.182112
**Irak2**	1.189369	2.62E-14	5.99E-13	2.28053	**Cwf19l1**	1.125673	0.000107	0.000391	2.182032
**Mlx**	1.188727	1.04E-11	1.36E-10	2.279514	**Ppp2r2a**	1.125419	9.06E-11	9.68E-10	2.181648
**Gphn**	1.188423	1.46E-10	1.50E-09	2.279034	**Akirin1**	1.125145	2.84E-12	4.18E-11	2.181235
**Tbrg1**	1.186124	2.21E-13	4.13E-12	2.275406	**Mat2a**	1.123907	6.75E-13	1.15E-11	2.179363
**Shisa5**	1.185105	1.09E-14	2.73E-13	2.273799	**2200002D01Rik**	1.120805	0.002984	0.0086	2.174683
**Eif5**	1.183358	2.13E-12	3.21E-11	2.271048	**Pfdn6**	1.119896	5.80E-11	6.47E-10	2.173313
**Atp11b**	1.182639	2.89E-13	5.31E-12	2.269915	**Itfg2**	1.117436	1.21E-07	7.16E-07	2.169611
**Nif3l1**	1.181097	6.52E-11	7.17E-10	2.267491	**Stk19**	1.117349	1.29E-09	1.10E-08	2.16948
**Calcrl**	1.179194	1.45E-11	1.82E-10	2.264502	**Spp1**	1.117205	4.97E-12	6.96E-11	2.169263
**Lrrc32**	1.178986	4.18E-06	1.91E-05	2.264176	**Zfp821**	1.115305	1.50E-08	1.04E-07	2.166408
**Dtnb**	1.178649	0.000219	0.00076	2.263648	**Leng1**	1.114505	1.39E-10	1.44E-09	2.165207
**Fyb**	1.178519	2.99E-11	3.54E-10	2.263444	**Acaa1a**	1.114339	1.95E-13	3.70E-12	2.164958
**Adamts9**	1.177614	0.000671	0.002143	2.262024	**Ubc**	1.114185	1.56E-11	1.94E-10	2.164727
**Gpr176**	1.175076	7.03E-08	4.33E-07	2.258048	**Mrpl39**	1.113418	4.45E-11	5.11E-10	2.163577
**Polr2g**	1.173633	2.85E-12	4.19E-11	2.255791	**Vdac3**	1.110623	3.63E-13	6.55E-12	2.159389
**Nfkbil1**	1.172923	3.25E-09	2.56E-08	2.254681	**Pdss1**	1.108873	0.000202	0.000705	2.156772
**Nploc4**	1.172531	1.89E-13	3.59E-12	2.254067	**Aptx**	1.108429	7.81E-08	4.77E-07	2.156107
**Zfand3**	1.167883	1.96E-10	1.96E-09	2.246817	**Manbal**	1.107214	1.55E-11	1.93E-10	2.154292
**Hsph1**	1.166608	1.22E-11	1.57E-10	2.244832	**Igsf6**	1.105102	2.13E-12	3.22E-11	2.15114
**Psmb5**	1.166466	1.10E-12	1.79E-11	2.244612	**Dync1h1**	1.10432	1.07E-10	1.12E-09	2.149975
**Smg9**	1.166184	2.92E-11	3.47E-10	2.244173	**Rbpms**	1.104262	1.16E-09	9.99E-09	2.149889
**Ccdc71l**	1.166121	1.01E-13	2.03E-12	2.244075	**Abcc4**	1.103938	6.95E-08	4.29E-07	2.149405
**Aff1**	1.16598	1.32E-12	2.10E-11	2.243856	**Cep85l**	1.102888	1.35E-08	9.44E-08	2.147842
**Zfp991**	1.165194	1.24E-11	1.58E-10	2.242634	**Gm45902**	1.102574	0.000709	0.002258	2.147375
**Ercc8**	1.162074	0.000267	0.000911	2.237788	**Tent2**	1.099736	5.49E-13	9.51E-12	2.143155
**Ebna1bp2**	1.161873	1.43E-12	2.25E-11	2.237477	**Ninj1**	1.09766	6.95E-12	9.33E-11	2.140073
**H60b**	1.161155	0.001861	0.005557	2.236364	**Psmc6**	1.096688	1.41E-12	2.23E-11	2.138632
**Atp6v1e1**	1.161094	6.76E-13	1.15E-11	2.236269	**Ndel1**	1.096024	3.07E-13	5.63E-12	2.137648
**Id2**	1.160396	5.27E-13	9.18E-12	2.235187	**Sbds**	1.095763	2.88E-12	4.23E-11	2.137261
**Gna13**	1.160359	7.34E-14	1.53E-12	2.23513	**Seh1l**	1.095391	7.21E-12	9.64E-11	2.13671

Gene	log2 (FC)	P-value	FDR	FC	Gene	log2 (FC)	P-value	FDR	FC
**Nub1**	1.094791	9.77E-14	1.97E-12	2.135822	**Twsg1**	1.04955	2.10E-11	2.56E-10	2.069884
**Tet2**	1.094137	3.74E-08	2.43E-07	2.134854	**Tefm**	1.049305	1.99E-06	9.59E-06	2.069533
**Usp16**	1.093933	1.58E-11	1.97E-10	2.134552	**Nop58**	1.047791	3.73E-09	2.89E-08	2.067362
**Agfg1**	1.093207	6.33E-14	1.34E-12	2.133477	**Sesn2**	1.047782	1.46E-11	1.84E-10	2.067349
**Ptk2**	1.092702	7.98E-10	7.06E-09	2.132731	**Tbrg4**	1.047712	6.45E-09	4.77E-08	2.067248
**B3gnt2**	1.092566	1.17E-12	1.90E-11	2.132531	**Pgam5**	1.04692	1.44E-11	1.81E-10	2.066114
**Scpep1**	1.092362	3.95E-13	7.08E-12	2.132228	**Eif3d**	1.046439	8.21E-13	1.38E-11	2.065425
**Dennd5b**	1.091523	0.000884	0.002774	2.130989	**Foxred1**	1.045135	3.48E-09	2.72E-08	2.063559
**Ctps**	1.09123	2.15E-06	1.03E-05	2.130556	**Rad23b**	1.044491	5.78E-14	1.24E-12	2.062639
**Zfp455**	1.09113	6.26E-08	3.90E-07	2.130408	**Ypel5**	1.044347	1.28E-12	2.05E-11	2.062433
**Mrpl47**	1.091043	1.88E-08	1.28E-07	2.130279	**Mrpl30**	1.043559	2.35E-10	2.32E-09	2.061307
**Nop56**	1.090129	4.19E-10	3.94E-09	2.12893	**Csrp1**	1.042813	3.73E-08	2.42E-07	2.060241
**Prdm1**	1.090123	2.21E-09	1.79E-08	2.128922	**Dus4l**	1.041987	0.000545	0.001765	2.059061
**Aifm2**	1.089881	1.07E-09	9.27E-09	2.128565	**Ppif**	1.041975	7.33E-08	4.49E-07	2.059045
**Ticam2**	1.089431	2.97E-14	6.71E-13	2.127901	**Atrnl1**	1.041604	2.24E-09	1.81E-08	2.058515
**Tfam**	1.089256	3.80E-10	3.61E-09	2.127643	**Ccdc93**	1.041341	2.80E-11	3.35E-10	2.058139
**Krtcap2**	1.086864	1.42E-10	1.46E-09	2.124118	**Ctsh**	1.040666	1.03E-09	8.96E-09	2.057178
**Plcd1**	1.08607	9.42E-05	0.000346	2.122949	**Alg9**	1.04058	4.12E-08	2.65E-07	2.057055
**Treml2**	1.084911	5.14E-06	2.32E-05	2.121244	**Rap1b**	1.040048	1.56E-12	2.44E-11	2.056296
**Stxbp1**	1.084103	1.99E-07	1.14E-06	2.120057	**Sgpl1**	1.039772	1.13E-12	1.83E-11	2.055902
**Eif2b3**	1.083758	2.24E-08	1.51E-07	2.119549	**Dpep2**	1.03858	3.35E-12	4.85E-11	2.054204
**Mrps30**	1.082206	4.28E-10	4.02E-09	2.117272	**Amotl2**	1.038269	0.000529	0.001718	2.053762
**Smurf1**	1.082095	1.26E-11	1.60E-10	2.117108	**Ccdc59**	1.037937	1.42E-10	1.47E-09	2.053289
**Rbm43**	1.081833	5.16E-12	7.20E-11	2.116723	**Fgfrl1**	1.037874	0.000593	0.001909	2.053199
**Tcof1**	1.081566	4.70E-11	5.36E-10	2.116333	**Zc3h7a**	1.03731	8.86E-13	1.47E-11	2.052397
**Rap2c**	1.081341	9.73E-13	1.60E-11	2.116002	**Snrnp27**	1.036511	1.14E-10	1.19E-09	2.051261
**Appl1**	1.081032	3.00E-12	4.40E-11	2.11555	**Pop4**	1.036452	9.39E-11	9.99E-10	2.051177
**Maip1**	1.079249	7.47E-10	6.64E-09	2.112936	**Rhou**	1.03637	4.77E-11	5.41E-10	2.051061
**Sertad1**	1.078715	5.87E-09	4.38E-08	2.112153	**Trit1**	1.035707	2.46E-06	1.16E-05	2.050118
**Ralgapa2**	1.07842	3.90E-10	3.69E-09	2.111723	**Dimt1**	1.034835	3.96E-08	2.56E-07	2.048879
**Med21**	1.076874	1.05E-08	7.51E-08	2.109461	**Dda1**	1.034667	9.51E-13	1.57E-11	2.048641
**Rhoq**	1.075327	8.79E-13	1.46E-11	2.1072	**Zfp653**	1.033937	3.65E-09	2.83E-08	2.047604
**Yars2**	1.075298	6.27E-09	4.65E-08	2.107157	**Sbno2**	1.03258	1.71E-11	2.11E-10	2.045679
**Jmjd6**	1.074677	4.31E-10	4.04E-09	2.10625	**Zfyve27**	1.032403	4.11E-11	4.75E-10	2.045429
**Pogk**	1.074588	4.64E-10	4.31E-09	2.106121	**Yju2**	1.032181	9.91E-08	5.96E-07	2.045114
**Rab5c**	1.073988	1.42E-12	2.25E-11	2.105245	**Hsd17b12**	1.032051	8.44E-13	1.41E-11	2.044929
**Mfsd8**	1.073932	6.47E-09	4.78E-08	2.105163	**Plk4**	1.031623	3.48E-05	0.000137	2.044323
**Triobp**	1.07308	5.26E-12	7.31E-11	2.10392	**Cdc42bpg**	1.030376	1.93E-05	7.89E-05	2.042556
**Sgcb**	1.07251	1.74E-10	1.77E-09	2.103088	**Eif1a**	1.029954	9.88E-12	1.29E-10	2.041959
**Cldnd1**	1.072292	8.58E-11	9.23E-10	2.102772	**Adrm1**	1.028931	7.65E-13	1.29E-11	2.040512
**Snrpa1**	1.072034	1.90E-09	1.56E-08	2.102395	**Lrrc59**	1.028721	3.51E-13	6.36E-12	2.040214
**Chac2**	1.071922	2.43E-06	1.15E-05	2.102232	**Prdm4**	1.028371	4.67E-11	5.33E-10	2.039719
**F11r**	1.071741	0.000341	0.001143	2.101969	**Usp53**	1.028061	2.38E-06	1.13E-05	2.039282
**Casp8**	1.069621	1.54E-11	1.93E-10	2.098882	**Ppp2r2d**	1.026604	3.31E-11	3.88E-10	2.037224
**H2-D1**	1.068611	1.33E-11	1.68E-10	2.097413	**Kcnh7**	1.025317	1.02E-05	4.38E-05	2.035407
**Chchd4**	1.068354	1.79E-09	1.48E-08	2.097039	**Susd6**	1.023354	2.71E-12	4.00E-11	2.032638
**Pfkfb3**	1.066427	1.91E-08	1.30E-07	2.094241	**Uxt**	1.021057	9.99E-08	6.01E-07	2.029406
**Mrps22**	1.0663	2.58E-09	2.06E-08	2.094056	**Rilpl1**	1.020478	4.99E-05	0.000191	2.028591
**Npc2**	1.065863	1.47E-11	1.85E-10	2.093422	**Znhit1**	1.020336	9.28E-11	9.89E-10	2.028391
**Atg10**	1.065074	9.53E-09	6.83E-08	2.092278	**Gm9803**	1.019992	1.01E-05	4.31E-05	2.027908
**Zswim4**	1.06505	1.09E-10	1.14E-09	2.092242	**Flt3l**	1.019951	5.99E-07	3.15E-06	2.02785
**Cdv3**	1.064493	1.55E-12	2.43E-11	2.091435	**Tlcd2**	1.019119	0.002371	0.006951	2.026681
**Kcmf1**	1.063557	1.44E-13	2.80E-12	2.090078	**Moap1**	1.017748	7.67E-06	3.35E-05	2.024756
**Suco**	1.061662	2.54E-10	2.48E-09	2.087334	**Kansl2**	1.016107	2.93E-09	2.32E-08	2.022454
**Laptm4a**	1.060885	1.13E-11	1.46E-10	2.086212	**Emc7**	1.015574	8.74E-11	9.38E-10	2.021707
**Ptges3**	1.058669	6.09E-12	8.30E-11	2.083009	**Lin9**	1.01498	0.000352	0.001177	2.020875
**Dclre1b**	1.056831	3.52E-09	2.74E-08	2.080356	**Tmsb4x**	1.013964	5.66E-09	4.24E-08	2.019453
**Atf3**	1.056748	4.22E-11	4.86E-10	2.080237	**Mreg**	1.013632	2.24E-08	1.51E-07	2.018988
**Mcoln2**	1.054901	4.13E-12	5.89E-11	2.077576	**Abhd16a**	1.013143	1.27E-09	1.09E-08	2.018303
**Fnbp1l**	1.054088	8.13E-07	4.16E-06	2.076405	**Cops5**	1.01188	5.93E-12	8.10E-11	2.016537
**Zfp993**	1.052406	1.27E-08	8.91E-08	2.073985	**Daam1**	1.011353	1.98E-10	1.98E-09	2.0158
**Tmem138**	1.051882	7.16E-05	0.000267	2.073233	**Rps6ka2**	1.011157	2.34E-11	2.84E-10	2.015527
**Tspan31**	1.050767	1.07E-12	1.74E-11	2.071631	**Fubp1**	1.010577	2.20E-13	4.12E-12	2.014717
**Psmc1**	1.050433	9.61E-13	1.58E-11	2.071151	**Pprc1**	1.009959	5.86E-10	5.32E-09	2.013855
**Rps19bp1**	1.05016	4.53E-10	4.23E-09	2.07076	**Txnl4b**	1.009297	1.03E-08	7.35E-08	2.01293

Gene	log2 (FC)	P-value	FDR	FC	Gene	log2 (FC)	P-value	FDR	FC
**Mettl22**	1.009183	6.00E-07	3.16E-06	2.012771	**Tiparp**	1.004493	7.22E-12	9.66E-11	2.006238
**Rnf149**	1.008833	3.17E-12	4.62E-11	2.012282	**Klf6**	1.004443	5.33E-13	9.26E-12	2.006169
**Pde5a**	1.008611	5.57E-06	2.50E-05	2.011972	**Rab22a**	1.003558	1.18E-12	1.91E-11	2.004938
**Rps6ka4**	1.008219	2.05E-12	3.11E-11	2.011427	**Ncf1**	1.001163	3.01E-11	3.56E-10	2.001613
**9130401M01Rik**	1.007552	1.37E-11	1.73E-10	2.010497	**Glce**	1.001079	3.27E-09	2.57E-08	2.001496
**Bloc1s4**	1.007386	1.73E-10	1.76E-09	2.010266	**C1s1**	1.000895	0.000367	0.001221	2.001241
**Dxo**	1.007187	5.73E-09	4.29E-08	2.009988	**Txndc9**	1.000717	2.32E-10	2.29E-09	2.000994
**Rcsd1**	1.00633	1.67E-09	1.39E-08	2.008795	**Rabl3**	1.000061	1.07E-05	4.55E-05	2.000085
**Dock10**	1.006082	7.58E-11	8.23E-10	2.008449					

FC: Fold change; FDR: False discovery rate
